# Adherence to prescription guidelines and achievement of treatment goals among persons with coronary heart disease in Tromsø 7

**DOI:** 10.1186/s12872-021-01866-1

**Published:** 2021-01-21

**Authors:** Elisabeth Pedersen, Beate Hennie Garcia, Kjell H. Halvorsen, Anne Elise Eggen, Henrik Schirmer, Marit Waaseth

**Affiliations:** 1 Department of Pharmacy, UiT The Arctic University of Norway, Tromsø, Norway; 2 Department of Community Medicine, UiT The Arctic University of Norway, Tromsø, Norway; 3 Department of Cardiology, Akershus University Hospital, Lørenskog, Norway; 4 Institute of Clinical Medicine Campus Ahus, University of Oslo, Oslo, Norway

**Keywords:** Coronary heart disease, Prescription guidelines, Blood pressure, Antihypertensive agents, Lipid-lowering drugs, Low-density lipoprotein (LDL)-cholesterol

## Abstract

**Background:**

Adherence to clinical practice guidelines for coronary heart disease (CHD) reduces morbidity, mortality and treatment costs. We aimed to describe and compare adherence to prescription guidelines for persons with CHD, and explore its association with treatment goal achievement.

**Method:**

We included all participants reporting myocardial infarction, angina, percutaneous coronary intervention and/or coronary artery bypass surgery in the seventh wave of the Tromsø Study (2015–2016, n = 1483). Medication use and treatment goal measures (blood pressure, low-density lipoprotein (LDL)-cholesterol and HbA1c) were compared to clinical practice guidelines on secondary CHD prevention. Propensity score matched logistic regression was used to assess the association between the use of antihypertensive drugs and achievement of treatment goal for blood pressure, and the use of lipid-lowering drugs (LLDs) and achievement of treatment goal for LDL-cholesterol.

**Results:**

The prevalence of pharmacological CHD treatment was 76% for LLDs, 72% for antihypertensive drugs and 66% for acetylsalicylic acid. The blood pressure goal (< 140/90 mmHg, < 140/80 mmHg if diabetic) was achieved by 58% and the LDL-cholesterol goal (< 1.8 mmol/l or < 70 mg/dL) by 9%. There was a strong association between using LLDs and achieving the treatment goal for LDL-cholesterol (OR 14.0, 95% CI 3.6–54.7), but not between using antihypertensive drugs and blood pressure goal achievement (OR 1.4, 95% CI 0.7–2.7).

**Conclusion:**

Treatment goal achievement of LDL-cholesterol and blood pressure was low, despite the relatively high use of LLDs and antihypertensive drugs. Further research is needed to find the proper actions to increase achievement of the treatment goals.

## Background

Coronary heart disease (CHD) is one of the leading causes of deaths worldwide and a common cause of hospital admissions [1, 2]. The major modifiable risk factors are high blood pressure and cholesterol levels, tobacco smoking, diabetes mellitus, low physical activity, obesity, and unhealthy diet [3]. Over the recent decades, the world has witnessed a substantial reduction in CHD morbidity and mortality which is partially attributed to strategies based on lowering of blood pressure and cholesterol, as well as successful acute treatment [4, 5].

Clinical practice guidelines for CHD promote risk factor reduction, both in terms of lifestyle changes and medication use. Lipid-lowering drugs (LLDs), antihypertensive drugs and acetylsalicylic acid (ASA) comprise the recommended secondary prevention after both myocardial infarction (MI) and coronary artery intervention like percutaneous coronary intervention (PCI) or coronary artery bypass surgery (CABG) [6]. Adherence to these prescription guidelines has been shown to prevent premature mortality, reduce morbidity and healthcare costs, and improve the patient’s quality of life [6].

The European survey of cardiovascular disease prevention and diabetes (EUROASPIRE) is the largest European CHD survey, and it has evaluated the implementation of clinical guidelines in CHD patients five times since 1995–1996 [7]. The most recent EUROASPIRE survey showed that > 80% of CHD patients use antihypertensive drugs and LLDs. The survey also showed that achievement of the recommended treatment goals is low, where 58% of the patients reach the treatment goal for blood pressure and 29% the treatment goal for low-density lipoprotein (LDL)-cholesterol. Similar results have also been found in a Norwegian study, where 93% used both antihypertensive drugs and LLDs, while 54% reached the treatment goal for blood pressure and 43% reached the treatment goal for LDL-cholesterol [8].

Studies have shown an increase in treatment goal achievement in line with a decrease in blood pressure and cholesterol in the general population [9–13], but mainly describe adherence to clinical prescription guidelines and treatment goal achievement on an aggregated and not an individual level. Associations between treatment goal achievement and adherence to guidelines concerning prescription have also not been explored.

The aim of this study was to describe and compare adherence to prescription guidelines for persons with CHD and explore its association with treatment goal achievement for blood pressure and LDL-cholesterol.

## Methods

### Study setting and study population

The Tromsø Study is a Norwegian population-based epidemiological health study that has been conducted seven times from 1974 to 2016 [14]. The population of the Tromsø Study consists of inhabitants in the municipality of Tromsø in North Norway, a university town with approximately 70 000 inhabitants, and it is considered representative for a white, urban Northern European population [15].

The current study is a cross-sectional study applying data collected from participants in the seventh wave of the Tromsø Study (Tromsø 7). Tromsø 7 was conducted in 2015 and 2016 and invited all inhabitants in the municipality aged 40 years or older (n = 32,591) to participate. Response rate was 65% (n = 21,083). Participants answered several questionnaires, donated blood samples and went through a range of anthropometric measurements (height, weight, body circumferences and clinical examinations). Links to the questionnaires can be found at the Tromsø Study’s webpage [16]. We included persons who self-reported CHD in the mandatory questionnaire, i.e. previous MI, present or previous angina pectoris (AP) and/or previous PCI or CABG. Participants with self-reported diabetes or reporting use of antidiabetic drugs and those with self-reported hypertension were defined as subgroups in some analyses.

**Fig. 1:**
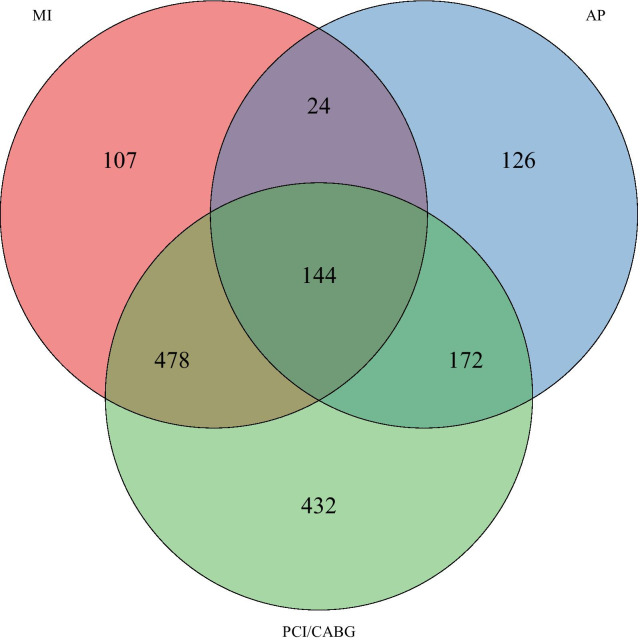
Distribution of participants across coronary heart disease groups in Tromsø 7. AP, angina pectoris; CABG, coronary artery bypass surgery; MI, myocardial infarction; PCI, percutaneous coronary intervention

We included a total of 1483 (7.0%) participants with CHD; 753 with previous MI, 466 with AP and 1226 with previous PCI and/or CABG, some of them indicating more than one disease (Fig. 1). We divided our study population into three subgroups; previous MI (n = 753), PCI or CABG but no previous MI (n = 604) and only AP with no previous MI, PCI or CABG (n = 126). Of the 1483 participants with CHD, 214 (14%) had diabetes, and 827 (56%) reported having hypertension.

### Data extraction

We extracted information about blood pressure measurements from clinical examinations, LDL-cholesterol and HbA1c from blood samples and self-reported data from questionnaires. The questionnaire data included information about present and previous diseases, medication use, health concerns, use of health services, diet, physical activity, smoking status, alcohol consumption and socio-demography.

Prevalent users of LLDs, antihypertensive drugs and antidiabetic drugs were defined by two approaches; (1) by including those who replied “currently” when asked “*Do you use, or have you used cholesterol lowering drugs/blood pressure lowering drugs/insulin or tablets for diabetes?*” (answering options were “currently”, “previously, not now” and “never used”) or (2) mentioning the brand name of medications within these drug classes when asked to write down the brand names for all medications used regularly during the previous four weeks. Prevalent ASA use was defined as answering “yes” when asked *“If you have used analgesics and anti-inflammatory medication regularly in the past year—did you use “Baby” or low dose acetylsalicylic acid (ASA) Acetylsalisylsyre® Albyl-E® Asasantin Retard® (75/160 mg per tablet)?”* (answering options were “yes” and “no”), or mentioning a brand name for ASA when asked to write down brand names for all the medications used regularly during the previous four weeks.

Brand names were recoded by trained personnel using the anatomical therapeutic chemical (ATC) classification system and categorised into medication groups. LLDs included statins and other LLDs. Antihypertensive drugs included angiotensin converting enzyme (ACE) inhibitors, angiotensin receptor blockers (ARBs), beta-blockers, calcium channel blockers (CCBs), thiazides, other diuretics and other antihypertensives (Additional file 1: Table S1).

In Tromsø 7, blood pressure was measured with an automated digital device (Dinamap ProCare 300 monitor, GE Healthcare, Norway) [9]. Three consecutive measurements were taken. Blood pressure was defined as the mean of the last two measurements. If only the third measurement was missing (n = 2), the second measurement was used. When both the second and third measurement, but not the first, was missing (n = 1), the first measurement was used. LDL-cholesterol was collected and analyzed by trained personnel using enzymatic colorimetric methods with commercial kits on a Cobas 8000 c702 (Roche Diagnostics GmbH, Mannheim, Germany) from non-fasting venous blood samples. The analysis was performed at the Department of Laboratory Medicine, University Hospital of North Norway, Tromsø, Norway (ISO certification NS-EN ISO 15189:2012) [10].

Treatment goal achievement and medication use were assessed based on the European Guidelines on cardiovascular disease prevention in clinical practice from 2012 (Table 1) [6]. At the time of data collection, there were no Norwegian clinical guidelines for secondary prevention of CHD.

**Table 1:** Recommendations in guidelines on cardiovascular disease prevention by the European Society of Cardiology in 2012 [6]

Medication prescription
Acetylsalicylic acid
Lipid-lowering drugs
Statins
Antihypertensive drugs (if hypertension)
ACE inhibitor/ARB (first choice for diabetics)
Beta-blockers
Calcium channel blockers
Diuretics

Treatment goals
Blood pressure
< 140/90 mmHg (< 140/80 mmHg if diabetic)
LDL-cholesterol
< 1.8 mmol/l (< 70 mg/dL)
HbA1c (if diabetic)
< 7%

ACE, angiotensin converting enzyme; ARB, angiotensin receptor blocker; HbA1c, glycated haemoglobin; LDL, low-density lipoprotein

### Statistical method

Descriptive statistics are presented as frequencies with proportions (%) (categorical variables) and means with standard deviation (SD) (continuous variables).

Chi square tests were used to examine the relationship between achievement of treatment goals for blood pressure and LDL-cholesterol and use of LLDs, antihypertensive drugs and ASA, and between achievement of treatment goals and disease group. Significance level was set to 5%.

Logistic regression was used to explore the association between use of antihypertensive drugs and achievement of treatment goal for blood pressure, as well as the association between use of LLDs and achievement of treatment goal for LDL-cholesterol. Participants with missing measurements for blood pressure (n = 3) and LDL-cholesterol (n = 11) were excluded from the respective analyses. Propensity score matching was used to control for confounding from covariates including age, sex, body mass index (BMI), relevant comorbidities, diet, physical activity, use of health services, alcohol consumption and smoking (including use of smokeless tobacco) (for more information about the variables included, see Additional file 1: Table S2). The matching method used was nearest neighbour matching, and the procedure was performed with replacement and a caliper of 0.2.

Due to the high proportion of missing values in some of the covariates, imputation was needed to perform the analyses. If a factor was described by more than one variable (e.g. use of health services, tobacco, alcohol), these variables were combined. For instance, a participant reporting current smoking on at least one question regarding smoking habits would be categorised as a smoker. Multiple imputation by chained equations was then performed using the R packages mice and MatchIt.mice (see Additional file 1: Table S3 for variables included). Predictive mean matching was used to impute numeric variables, logistic regression for binary categorical variables, proportional odds model for ordered categorical variables and polytomous logistic regression for unordered categorical variables. Ten imputated datasets where created with 50 iterations. The analyses were then performed with the within approach, which means that the propensity score matching and logistic regression was performed in each imputed dataset and the results subsequently pooled together to an overall result. We used the non-imputed dataset for the descriptive analyses and chi square tests, and the imputed datasets for the regression analyses.

All descriptive statistical analyses were performed using SPSS version 25.0 (IBM Corp, 2017). Chi square tests, multiple imputation, propensity score matching and logistic regression were conducted using R (R Core Team (2019). R: A language and environment for statistical computing. R Foundation for Statistical Computing, Vienna, Austria. URL https://www.R-project.org/).

### Ethics

The study was approved by the Norwegian Data Protection Authority and the Regional Committee for Medical and Health Research Ethics of North Norway. All participants in the Tromsø Study have given written informed consent for their data to be used in research.

## Results

The basic characteristics of the study population and across the three CHD disease groups are shown in Table 2.

**Table 2:** Characteristics of the study population

	Total CHD population (n = 1483)		MI (n = 753)		PCI and/or CABG, but no MI (n = 604)		AP, but no MI, PCI or CABG (n = 126)	
Sex, n (%)								
Women	446	(30.1)	174	(23.1)	203	(33.6)	69	(54.8)
Age, mean (sd)	68.7	(10.8)	69.2	(10.1)	69.2	(10.6)	63.0	(13.7)
Smoking, n (%)								
Daily smoking	182	(12.3)	96	(12.7)	67	(11.1)	19	(15.1)
Smoked previously	860	(58.0)	472	(62.7)	334	(55.3)	54	(42.9)
Self-reported health, n (%)								
Excellent/good	705	(47.5)	343	(45.6)	311	(51.5)	51	(40.5)
Neither good nor bad	602	(40.6)	318	(42.2)	232	(38.4)	52	(41.3)
Bad/very bad	153	(10.3)	79	(10.5)	53	(8.8)	21	(16.7)
Comorbidities*, n (%)								
Hypertension	827	(55.8)	433	(57.5)	328	(54.3)	66	(52.4)
Heart failure	231	(15.6)	126	(16.7)	94	(15.6)	11	(8.7)
Atrial fibrillation	283	(19.1)	138	(18.3)	111	(18.4)	34	(27.0)
Stroke	123	(8.3)	65	(8.6)	49	(8.1)	9	(7.1)
Diabetes	214	(14.4)	123	(16.3)	80	(13.2)	11	(8.7)
Renal disease	114	(7.7)	68	(9.0)	35	(5.6)	11	(8.7)
Cancer	189	(12.7)	101	(13.4)	77	(12.7)	11	(8.7)
Medications, mean number of products (sd)	4.0	(2.9)	4.3	(3.0)	3.8	(2.8)	3.0	(2.9)
Clinical measurements, mean (sd)								
Systolic blood pressure, mmHg	136	(20.9)	135	(21.7)	137	(19.9)	133	(20.3)
Diastolic blood pressure, mmHg	74	(9.9)	75	(10.0)	74	(9.6)	76	(10.1)
Total cholesterol, mmol/l^†^	4.6	(1.1)	4.5	(1.1)	4.7	(1.1)	5.6	(1.3)
LDL-cholesterol, mmol/l^†^	2.9	(1.0)	2.7	(1.0)	2.9	(1.0)	3.7	(1.2)
HDL-cholesterol, mmol/l^†^	1.4	(0.4)	1.4	(0.4)	1.5	(0.5)	1.5	(0.4)
Triglycerides, mmol/l^‡^	1.6	(1.0)	1.7	(1.0)	1.5	(0.8)	1.8	(0.9)
HbA1c, %	6.1	(0.9)	6.1	(1.0)	6.0	(0.8)	5.8	(0.5)
Glucose, mmol/l^§^	6.0	(2.1)	6.2	(2.3)	6.0	(1.9)	5.6	(1.3)
BMI, kg/m^2^	28.4	(4.5)	28.5	(4.4)	28.1	(4.4)	28.7	(5.2)

AP, angina pectoris; BMI, body mass index; CABG, coronary artery bypass graft; CHD, coronary heart disease; HDL, high-density lipoprotein; LDL, low-density lipoprotein; MI, myocardial infarction; PCI, percutaneous coronary intervention; sd, standard deviation

^*^Self-reported relevant comorbidities for coronary heart disease, present or previous diseases. For diabetes: present disease or use of any antidiabetic drug

^†^To convert to mg/dL, multiply with 38.67

^‡^To convert to mg/dL, multiply with 88.57

^§^To convert to mg/dL, multiply with 18.02

**Fig. 2:**
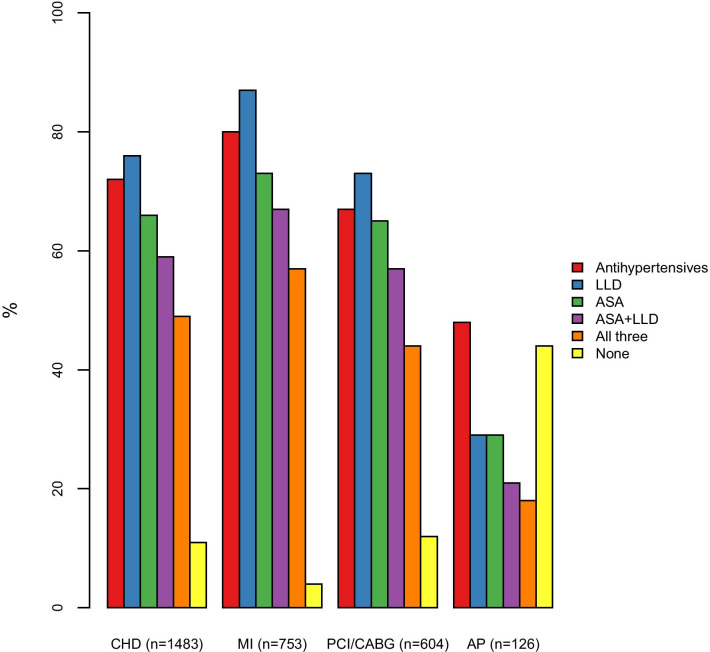
Proportion of participants using medications for CHD in total and across the CHD disease groups. AP, angina pectoris; ASA, acetylsalicylic acid; CABG, coronary artery bypass surgery; CHD, coronary heart disease; LLD, lipid-lowering drug; MI, myocardial infarction; PCI, percutaneous coronary intervention

Use of medications for CHD was highest among participants with previous MI and lowest among those with AP only, see Fig. 2. Of those with hypertension (n = 827), 92% used antihypertensive drugs. Among users of antihypertensive drugs, the drug classes included beta-blockers (63%), ACE-inhibitors or ARBs (49%), CCBs (22%), thiazides (16%), other (17%) and unknown (9%) antihypertensive drugs (for ATC-classification of medication groups, see Additional file 1: Table S1). Fifty-one percent used two or more medications from different antihypertensive drug classes. The most frequent class of LLD was statins (79% of LLD users), while 20% did not report which LLD they used. As 98% of those using LLDs in Norway use statins [17], these were assumed to be statin users. Among the LLD-users, 3% used another LLD in combination with a statin, while 1% used another LLD, but not a statin.

**Fig. 3:**
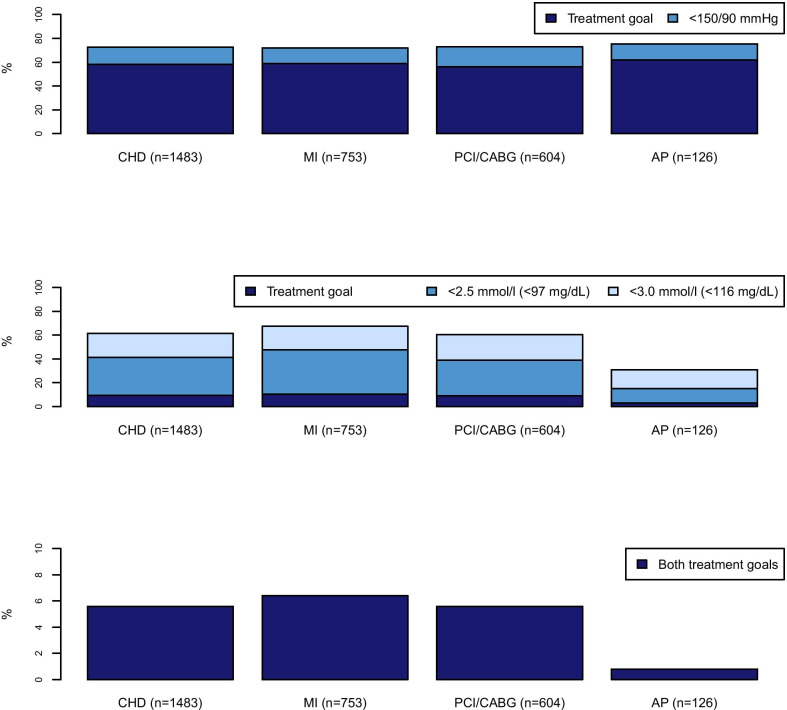
Proportion of participants achieving the treatment goals, in total and across the CHD disease groups. **a** Blood pressure, treatment goal < 140/90 mmHg (< 140/80 mmHg if diabetic). The panel also shows the proportion with blood pressure close to treatment goal. **b** Low-density lipoprotein (LDL)-cholesterol, treatment goal < 1.8 mmol/l (< 70 mg/dL). The panel also shows the proportion with LDL-cholesterol < 2.5 mmol/l (< 97 mg/dL, treatment goal for persons at high risk of CHD) and < 3.0 mmol/l (< 116 mg/dL, treatment goal for persons at moderate risk of CHD). **c** both treatment goals (y-axis 0–10%). AP, angina pectoris; CABG, coronary artery bypass surgery; CHD, coronary heart disease; MI, myocardial infarction; PCI, percutaneous coronary intervention

Blood pressure goal achievement (< 140/90 mmHg, < 140/80 mmHg in persons with diabetes) was highest among those with AP only and lowest among those without MI but previous PCI or CABG, see Fig. 3a. Among those reporting having hypertension, 49% reached the treatment goal for blood pressure. For comparison, Fig. 3a also includes the proportion having a blood pressure < 150/90 mmHg.

LDL-cholesterol goal achievement (< 1.8 mmol/l or < 70 mg/dL) was highest among those with previous MI and lowest among those with AP only, see Fig. 3b. For comparison, Fig. 3b also includes the proportions having LDL-cholesterol < 2.5 mmol/L (< 97 mg/dL) and < 3.0 mmol/L (< 116 mg/dL), the recommended treatment goals for persons at high and moderate risk of CHD respectively.

Thirty-eight percent of the study population did not reach any of the two treatment goals and 6% reached both (Fig. 3c). In the study population, 5% were completely in accordance with the guidelines, i.e. using ASA and LLDs and achieving both treatment goals. The proportion reaching both treatment goals was 9% (62) among those who used all three classes of drugs and 3% (21) among those who did not use all three (*p* < 0.001). Regarding CHD disease group, the proportion reaching both treatment goals was 6% (48) among those with a previous MI, 6% (34) among those with PCI and/or CABG and 1% (1) among those with AP (*p* = 0.04). Characteristics of participants achieving and not achieving the recommended treatment goals for LDL-cholesterol and blood pressure among those using LLDs and antihypertensive drugs are shown in Table 3.

**Table 3:** Characteristics of participants achieving and not achieving treatment goals among LLD and antihypertensive drug users

	Users of LLDs (n = 1133)				Users of antihypertensive drugs among participants with hypertension (n = 763)			
	Achieving LDL-goal (n = 136)		Not achieving LDL-goal (n = 991)		Achieving BP-goal (n = 379)		Not achieving BP-goal (n = 382)	
Sex, n (%)								
Women	29	(21.3)	262	(26.4)	123	(32.5)	132	(34.6)
Age, mean (sd)	69.7	(9.5)	69.4	(9.8)	68.0	(9.7)	71.6	(9.6)
Smoking, n (%)								
Daily smoking	15	(11.0)	114	(11.5)	52	(13.7)	29	(7.5)
Smoked previously	79	(58.1)	617	(62.3)	218	(57.5)	236	(61.8)
Self-reported health, n (%)								
Excellent/good	56	(41.2)	478	(48.2)	157	(41.4)	161	(42.1)
Neither good nor bad	68	(50.0)	403	(40.7)	175	(46.2)	177	(46.3)
Bad/very bad	9	(6.6)	99	(10.0)	44	(11.6)	40	(10.4)
Comorbidities*, n (%)								
Heart failure	20	(14.7)	176	(17.8)	72	(19.0)	65	(17.0)
Atrial fibrillation	23	(16.9)	181	(18.3)	84	(22.1)	76	(19.9)
Stroke	12	(8.8)	90	(9.1)	45	(11.9)	46	(12.0)
Diabetes	28	(20.6)	147	(14.8)	72	(19.0)	74	(19.4)
Renal disease	7	(5.2)	71	(7.1)	25	(6.6)	40	(10.5)
Cancer	18	(13.2)	124	(12.5)	44	(11.6)	60	(15.8)
Medications, mean number of products (sd)	5.3	(2.9)	4.4	(2.8)	5.0	(2.9)	5.0	(2.9)
Clinical measurements, mean (sd)								
Systolic blood pressure, mmHg	133	(18.5)	136	(21.0)	123	(11.7)	155	(15.4)
Diastolic blood pressure, mmHg	73	(9.2)	74	(9.8)	71	(8.5)	79	(9.6)
Total cholesterol, mmol/l^†^	3.3	(0.6)	4.5	(0.9)	4.4	(1.0)	4.6	(1.1)
LDL-cholesterol, mmol/l^†^	1.5	(0.2)	2.7	(0.8)	2.7	(0.9)	2.8	(1.0)
HDL-cholesterol, mmol/l^†^	1.5	(0.6)	1.4	(0.4)	1.4	(0.4)	1.4	(0.4)
Triglycerides, mmol/l^‡^	1.5	(1.6)	1.6	(0.9)	1.7	(0.9)	1.7	(1.1)
HbA1c, %	6.3	(1.0)	6.1	(0.9)	6.1	(1.0)	6.1	(1.0)
Glucose, mmol/l^§^	6.5	(2.7)	6.0	(2.1)	6.2	(2.3)	6.2	(2.2)
BMI, kg/m^2^	28.4	(4.6)	28.4	(4.3)	29.2	(4.6)	28.9	(4.4)

Percentages are calculated for columns

AP, angina pectoris; BMI, body mass index; BP, blood pressure; CABG, coronary artery bypass graft; CHD, coronary heart disease; HDL, high-density lipoprotein; LDL, low-density lipoprotein; LLD, lipid-lowering drugs; MI, myocardial infarction; PCI, percutaneous coronary intervention; sd, standard deviation

^*^Self-reported relevant comorbidities for coronary heart disease, present or previous diseases

^†^To convert to mg/dL, multiply with 38.67

^‡^To convert to mg/dL, multiply with 88.57

^§^To convert to mg/dL, multiply with 18.02

Among the participants with diabetes (n = 214), 50% achieved the treatment goal for blood pressure (< 140/80 mmHg) and 14% for LDL-cholesterol (< 1.8 mmol/L or < 70 mg/dL). Treatment goal for HbA1c (< 7%) was reached by 43% (for results on treatment goal achievement for HbA1c in the different CHD disease groups, see Additional file 1: Table S4). All three treatment goals were reached by 4%.

Logistic regression with propensity score matching showed that use of LLDs was significantly associated with treatment goal achievement for LDL-cholesterol, while the use of antihypertensive drugs among participants with hypertension was not associated with treatment goal achievement for blood pressure, see Table 4. Results from the propensity score matching can be found in Additional file 1: Tables S5 and S6.

**Table 4:** Pooled results from the logistic regression analyses of the propensity score matched multiple imputed datasets

Exposure variable	Outcome variable	Odds ratio	95% confidence interval
Use of lipid-lowering drugs	Achievement of treatment goal for LDL-cholesterol	14.0	3.6–54.7
Use of antihypertensive drugs	Achievement of treatment goal for blood pressure	1.3	0.7–2.6

Number of cases varied between datasets and can be found in Additional file 1: Tables S5 and S6

## Discussion

We have identified that a relatively high proportion of persons with CHD adhere to the recommended prescription guidelines. However, fewer of the participants in our study use LLDs, antihypertensive drugs and ASA compared to what has been found in other studies [7, 8, 18, 19]. The newest EUROASPIRE survey from 2019 found a proportion of use of 84% for LLDs, 95% for antihypertensive drugs and 93% for antiplatelet drugs [7]. The results from The NORwegian CORonary (NOR-COR) Prevention Study, STabilization of Atherosclerotic plaque By Initiation of darapLadIb TherapY (STABILITY) study and the prospeCtive observational LongitudinAl RegIstry oF patients with stable coronary arterY disease (CLARIFY) study are also similar to that of the EUROASPIRE [8, 18, 19]. Unlike our study, most of these studies attain their information about medication use from medical journals and the studies are usually conducted within a limited time frame after discharge from the hospital. As far as we know, ours is the first study to focus on persons with CHD in a general population, independent of time since diagnosis, and to investigate the participants’ own self-reported use of medications.

Compared with other studies, the use of ASA and other antiplatelet drugs in our study is especially low [7, 8, 18, 19]. The guidelines recommend use of ASA as secondary prevention for those who have had an MI, PCI or CABG, i.e., not including persons with only AP. In the current study, 70% within this subpopulation (n = 1357) used ASA, which is lower than what has been reported previously [7, 8]. Including other antiplatelet drugs increased the proportion of users to 71%, while the proportion of users of any antithrombotic drug (antiplatelets or anticoagulants) was 78%. Although there will always be some who cannot use antithrombotics, this user prevalence is lower than expected.

Despite high prevalence of use of antihypertensive drugs and LLDs, achievement of treatment goals was low, especially for LDL-cholesterol. This is also comparable to what has been found in other studies, though the level of achievement for LDL-cholesterol was lower in our study [7, 8, 20, 21]. Since our study population is defined as participants already having heart disease, they are considered to have a very high cardiovascular (CVD) risk, and the guidelines therefore recommend a treatment goal for LDL-cholesterol at < 1.8 mmol/L (< 70 mg/dL) or a reduction of ≥ 50% for LDL-cholesterol when the target cannot be reached [6]. As this is a cross-sectional study, we do not know the participants’ cholesterol levels at treatment initiation and are therefore not able to determine whether they have had a 50% reduction in LDL-cholesterol. However, even when applying a threshold of < 3 mmol/L (< 116 mg/dL), only 62% achieve the treatment goal (Fig. 3b). This indicates that many participants were far from reaching the recommended treatment goal.

The proportion of participants using antihypertensive drugs and achieving the treatment goals for blood pressure was comparable to what has been found in other studies [7–9, 18, 19]. These studies do not however explore the relationship between the two. We did not find a statistically significant relationship between using antihypertensive drugs and achieving the treatment goal for blood pressure. One plausible explanation for this is that participants who had been prescribed antihypertensive drugs probably had a higher baseline blood pressure than those who were not prescribed antihypertensive drugs. If so, some of the participants using antihypertensive drugs may have experienced a reduction in blood pressure, though not enough to reach the recommended treatment goal. Non-adherence could also be a potential explanation why we do not detect a statistically significant difference in achievement of treatment goal between participants using and not using antihypertensive drugs. Another possibility is that our population is too small, as so few persons with hypertension were not using antihypertensive drugs. This affects the propensity score matching, and makes it difficult to achieve comparable groups that are similar enough on all variables used in the propensity score. Further studies using a larger hypertensive population is therefore needed to confirm these results.

Of the three CHD disease groups, persons with previous MI, PCI or CABG have a higher risk of new major coronary events and require a closer follow-up than persons with only self-reported AP. We found that among participants within the PCI or CABG group, fewer persons reached the treatment goals for both blood pressure and LDL-cholesterol and fewer of these persons used LLDs, antihypertensive drugs and ASA compared to those with previous MI (Figs. 2 and 3). This suggests that these persons need closer follow-up.

### Strengths and limitations

We have used data from the Tromsø Study, a reliable population-based data source where measurements of blood pressure and cholesterol were performed by trained personnel and with standardized procedures and instruments. The Tromsø Study has a high attendance rate and is considered representative for an urban, white Northern European population [15].

Measurements of blood pressure and LDL-cholesterol were done objectively, which is also a strength of the study. So is the use of multiple imputation to avoid bias due to missing values and propensity score matching to control for confounding. Propensity score matching appropriately balances the covariates between treated and untreated participants and makes it possible to include more covariates than in a conventional multivariable logistic regression.

The major limitation in this study is that we do not have any information about blood pressure and LDL-cholesterol at treatment initiation, which restricts us to investigating the participants’ blood pressure and LDL-cholesterol at the time of their attendance in Tromsø 7.

We also lack information about the participants’ medication adherence; hence we do not know if the participants actually take their medication as prescribed. Non-adherence is likely to reduce their achievement of treatment goals.

Another limitation of the study is that most variables are self-reported, including CHD diagnosis and use of medications. We may have underestimated adherence to treatment guidelines through inclusion of some participants that are not actual CHD patients, or exclusion of participants who did not recall a previous CHD event. Such misclassification is less likely for life threatening conditions like MI, and although there may be some who reported MI when they have had a PCI/CABG (or vice versa), the extent would be limited and should not noteworthy alter the study results. Self-reported medication use could make the results susceptible to recall bias. For medications used for chronic conditions such as CHD, self-reported use of medications have shown good to very good agreement with prescription data [22], suggesting that recall bias should be a minor problem in our study.

A disadvantage with the statistical methods is that propensity score matching does not use all the observations. This is especially a problem when the groups of treated and untreated are very unevenly distributed, as in our study population (76% use LLDs and 92% of those with hypertension use antihypertensive drugs). To include as many observations as possible we performed matching with replacement. This procedure may introduce bias as several participants among medication users can be matched with the same non-user, and some may not be matched to anyone at all. However, a re-analysis without replacement gave very similar result, suggesting our results are valid (for results from this sensitivity analysis, see Additional file 1: Table S7). As propensity score matching only controls for measured confounders, our results might still be affected by unmeasured variables, which are only controllable through randomization.

## Conclusion

Despite high adherence to prescription guidelines and a strong association between use of LLDs and treatment goal achievement, the proportion who reaches the treatment goals is low among persons with CHD in a general population. Further research should include longitudinal studies to explore dosage regimens and medication adherence among persons with CHD over time.

## Supplementary Information

**Additional file 1: Table S1.** Overview of ATC-codes included in the three medication categories recommended for CHD based on the European Society of Cardiology: Guidelines on cardiovascular disease prevention in clinical practice (version 2012) [6]. **Table S2.** Variables included as covariates in propensity score. **Table S3.** Variables included in multiple imputation. **Table S4.** Achievement of treatment goal for HbA1c for participants with diabetes in the different CHD disease groups. **Table S5.** Results from propensity score matching of the ten imputed datasets for the logistic regression analysis of the association between use of lipid-lowering drugs and achieving the treatment goal for LDL-cholesterol. **Table S6.** Results from propensity score matching of the ten imputed datasets for the logistic regression analysis of the association between use of antihypertensive drugs and achieving the treatment goal for blood pressure among those with self-reported hypertension. **Table S7.** Pooled results from the sensitivity analysis for the logistic regression analyses of the multiple imputed datasets, using propensity score matching without replacement.

## Data Availability

The owner of the data is the Tromsø Study. We have permission to analyse the data according to the protocol submitted to the Tromsø Study; however, we do not have permission to share the data. Other interested researchers can request the data in the same manner if they comply with the requirements of the institution (https://en.uit.no/forskning/forskningsgrupper/sub?p_document_id=453582&sub_id=66 9706).

## Abbreviations

ACE: Angiotensin converting enzyme
AP: Angina pectoris
ARB: Angiotensin receptor blocker
ASA: Acetylsalicylic acid
ATC: Anatomical therapeutic chemical
BMI: Body mass index
CABG: Coronary artery bypass surgery
CCB: Calcium channel blocker
CHD: Coronary heart disease
CI: Confidence interval
CVD: Cardiovascular disease
EUROASPIRE: European survey of cardiovascular disease prevention and diabetes
HbA1c: Glycated haemoglobin
HDL: High-density lipoprotein
LDL: Low-density lipoprotein
LLD: Lipid-lowering drug
MI: Myocardial infarction
OR: Odds ratio
PCI: Percutaneous coronary intervention
SD: Standard deviation
SPSS: Statistical Package for the Social Sciences
