# Correction to: Adherence to prescription guidelines and achievement of treatment goals among persons with coronary heart disease in Tromsø 7

**DOI:** 10.1186/s12872-021-02214-z

**Published:** 2021-08-24

**Authors:** Elisabeth Pedersen, Beate Hennie Garcia, Kjell H. Halvorsen, Anne Elise Eggen, Henrik Schirmer, Marit Waaseth

**Affiliations:** 1 Department of Pharmacy, UiT The Arctic University of Norway, Tromsø, Norway; 2 Department of Community Medicine, UiT The Arctic University of Norway, Tromsø, Norway; 3 Department of Cardiology, Akershus University Hospital, Lørenskog, Norway; 4 Institute of Clinical Medicine Campus Ahus, University of Oslo, Oslo, Norway

## Correction to: BMC Cardiovasc Disord (2021) 21:44 https://doi.org/10.1186/s12872-021-01866-1

Following publication of the original article [1], the authors would like to correct some information in the fourth paragraph under ‘Data extraction’ in the methods section. We have found that the LDL-cholesterol concentrations included in the study were measured directly, not calculated from total cholesterol.

The information originally read:

Serum total cholesterol was analysed by CHOD-PAP enzymatic colorimetric methods with commercial kits (Roche Diagnostics GmbH, Mannheim, Germany) from non-fasting blood samples. The analysis was performed at the Department of Laboratory Medicine, University Hospital of North Norway, Tromsø, Norway. LDL-cholesterol concentration was then calculated according to Friedewald’s formula: LDL-cholesterol = total cholesterol − high-density lipoprotein cholesterol − (0.45 × triglycerides).

The information should read:

LDL-cholesterol was collected and analyzed by trained personnel using enzymatic colorimetric methods with commercial kits on a Cobas 8000 c702 (Roche Diagnostics GmbH, Mannheim, Germany) from non-fasting venous blood samples. The analysis was performed at the Department of Laboratory Medicine, University Hospital of North Norway, Tromsø, Norway (ISO certification NS-EN ISO 15189:2012).

The original article [1] has been corrected.
