# Supplementary material for: Adherence to prescription guidelines and achievement of treatment goals among persons with coronary heart disease in Tromsø 7
Source: BMC Cardiovasc Disord. 2021 Jan 21;21:44. doi: 10.1186/s12872-021-01866-1 (PMC7819182; doi:10.1186/s12872-021-01866-1)
Supplement: Supplementary file 1 — Additional file 1: Table S1. Overview of ATC-codes included in the three medication categories recommended for CHD based on the European Society of Cardiology: Guidelines on cardiovascular disease prevention in clinical practice (version 2012) [6]. Table S2. Variables included as covariates in propensity score. Table S3. Variables included in multiple imputation. Table S4. Achievement of treatment goal for HbA1c for participants with diabetes in the different CHD disease groups. Table S5. Results from propensity score matching of the ten imputed datasets for the logistic regression analysis of the association between use of lipid-lowering drugs and achieving the treatment goal for LDL-cholesterol. Table S6. Results from propensity score matching of the ten imputed datasets for the logistic regression analysis of the association between use of antihypertensive drugs and achieving the treatment goal for blood pressure among those with self-reported hypertension. Table S7. Pooled results from the sensitivity analysis for the logistic regression analyses of the multiple imputed datasets, using propensity score matching without replacement. [file 12872_2021_1866_MOESM1_ESM.docx]

Additional file: Table S1. Overview of ATC-codes included in the three medication categories recommended for CHD based on the European Society of Cardiology: Guidelines on cardiovascular disease prevention in clinical practice (version 2012) (6)

| Medication | | ATC-codes |
| --- | --- | --- |
| Acetylsalicylic acid^1^ | | |
|  | Acetylsalicylic acid | B01AC06 |
| Lipid lowering drugs | | |
|  | Statins | C10AA, C10BA |
|  | Other lipid lowering drugs | C10AC, C10AX |
|  | Unknown lipid lowering drugs^2^ |  |
| Antihypertensive drugs | | |
|  | ACE inhibitors and ARBs | C09 |
|  | Beta-blockers | C07 |
|  | Calcium channel blockers | C08, C09BB, C09DB, C09DX01, C09DX03 |
|  | Thiazides | C03A, C03EA, C07B, C09BA, C09DA, C09DX01, C09DX03 |
|  | Other antihypertensives^2^ | C02, C03C, C03D, C03EA, C03X |
|  | Unknown antihypertensives^2^ |  |
| ^1^ Also includes “yes” to question “If you have used analgesics and anti-inflammatory medication regularly in the past year - did you use “Baby” or low dose acetylsalicylic acid (ASA) Acetylsalisylsyre® Albyl-E® Asasantin Retard® (75/160 mg per tablet)?”  ^2^ When, instead of brand name, the participants in free text reported using medication interpretable as “blood pressure lowering”, “diuretics” or “cholesterol lowering”, it was registered under the respective medication category. | | |

Additional file: Table S2. Variables included as covariates in propensity score

| Variable name | Variable explanation | Coding |
| --- | --- | --- |
| AGE | Number of years at attendance date | Number |
| SEX_T7 | Sex | 1 : Male  0 : Female |
| BMI | Body mass index | Number |
| HEART_FAILURE_T7 | Have you ever had, or do you have heart failure? | 0 : No  1 : Yes, now  2 : Yes, previously |
| ATRIAL_FIBRILLATION_T7 | Have you ever had, or do you have atrial fibrillation? | 0 : No  1 : Yes, now  2 : Yes, previously |
| STROKE_T7 | Do you have, or have you had a cerebral stroke/brain haemorrhage? | 0 : No  2 : Yes, previously |
| KIDNEY_DISEASE_T7 | Do you have, or have you had a kidney disease, not including urinary tract infection (UTI)? | 0 : No  1 : Yes, now  2 : Yes, previously |
| CANCER_T7 | Have you ever had, or do you have cancer? | 0 : No  1 : Yes, now  2 : Yes, previously |
| DIABETES_impu_new^1^ | Do you have, or have you had diabetes? | 0 : No  1 : Yes, now  2 : Yes, previously |
| Cons_GP_Times_impu^1^ | If you have visited a general practitioner (GP) the past year, how many visits have you made? | Number |
| Cons_Emergency_Times_impu^1^ | If you have visited an emergency room the past year, how many visits have you made? | Number |
| Cons_Hospital_Times_impu^1^ | If you have been admitted to a hospital the past year, how many times? | Number |
| Cons_Specialist_Times_impu^1^ | If you have visited another medical specialist than a general practitioner (GP) or a psychologist or psychiatrist (not at a hospital) the past year, how many visits have you made? | Number |
| Cons_Clinic_Times_impu^1^ | Have you during the past year visited a hospital out-patient clinic, other than psychiatric department? | Number |
| Alcohol_frequency_impu^1^ | How often do you usually drink alcohol? | 1 : Never  2 : Monthly or less frequently  3 : 2-4 times a month  4 : 2-3 times a week  5 : 4 or more times a week |
| Alcohol_units_impu^1^ | How many units of alcohol (1 beer, glass of wine or drink) do you usually drink when you drink alcohol? | 1 : 1-2  2 : 3-4  3 : 5-6  4 : 7-9  5 : 10 or more |
| Alcohol_units_6_impu^1^ | How often do you drink 6 units alcohol or more in one occasion? | 1 : Never  2 : Less frequently than monthly  3 : Monthly  4 : Weekly  5 : Daily or almost daily |
| Smoke_impu^1^ | Do you/did you smoke daily/sometimes? | 1 : Now, daily  2 : Now, sometimes  3 : Previously  4 : Never |
| Snuff_chewing_tobacco_impu^1^ | Have you used or do you use snuff or chewing tobacco daily/sometimes? | 1 : Now, daily  2 : Now, sometimes  3 : Previously  4 : Never |
| Cod_liver_oil_omega3_impu^1^ | Do you use cod liver oil or cod liver oil capsules or Omega 3 capsules (fish oil, seal oil)? | 0 : No  1 : Sometimes  2 : Daily during the winter season  3 : Daily |
| FRUIT_UNITS_T7 | How many units of fruit or vegetables do you eat per day (average). (E.g. an apple, bowl of salad)? | Number |
| RED_MEAT_T7 | How often do you usually eat red meat (all products from beef, mutton, pork)? | 1 : 0-1 times per month  2 : 2-3 times per month  3 : 1-3 times per week  4 : 4-6 times per week  5 : Once a day or more |
| FRUITS_VEG_BERRY_T7 | How often do you usually eat fruits, vegetables and berries? | 1 : 0-1 times per month  2 : 2-3 times per month  3 : 1-3 times per week  4 : 4-6 times per week  5 : Once a day or more |
| LEAN_FISH_T7 | How often do you usually eat lean fish (cod, saithe)? | 1 : 0-1 times per month  2 : 2-3 times per month  3 : 1-3 times per week  4 : 4-6 times per week  5 : Once a day or more |
| FAT_FISH_T7 | How often do you usually eat fat fish (salmon, trout, redfish, mackerel, herring, halibut)? | 1 : 0-1 times per month  2 : 2-3 times per month  3 : 1-3 times per week  4 : 4-6 times per week  5 : Once a day or more |
| PHYS_ACTIVITY_LEISURE_T7 | Describe your exercise and physical exertion in leisure time over the last year. If your activity varies throughout the year, give an average. | 1 : Reading, watching TV/screen or other sedentary activity?  2 : Walking, cycling, or other forms of exercise at least 4 hours a week? (including walking or cycling to place of work, Sunday-walking, etc.)  3 : Participation in recreational sports, heavy gardening, snow shoveling etc at least 4 hours a week.  4 : Participation in hard training or sports competitions, regularly several times a week? |
| EXERCISE_T7 | How often do you exercise (i.e walking, skiing, swimming or training/sports)? | 1 : Never  2 : Less than once a week  3 : Once a week  4 : 2-3 times a week |
| ^1^ Combination of several variables from Tromsø 7 | | |

Additional file: Table S3. Variables included in multiple imputation

| Variable name | Variable explanation | Coding | Missing before imputation in CHD population (n=1483), n (%) | | Missing before imputation in hypertensive CHD population (n=827), n (%) | |
| --- | --- | --- | --- | --- | --- | --- |
| SEX_T7 | Sex | 1 : Male  0 : Female | 0 | (0.0) | 0 | (0.0) |
| TIME_LAST_MEAL_T7 | Time since last meal | 0 : < 1 hour  1 : 1-1.59 hours  2 : 2-2.59 hours  3 : 3-3.59 hours  4 : 4-4.59 hours  5 : 5-5.59 hours  6 : 6-6.59 hours  7 : 7-7.59 hours  8 : 8-8.59 hours  9 : 9+ hours | 2 | (0.1) | 1 | (0.1) |
| S_LDL_T7^1^ | Serum low density lipoprotein cholesterol (mmol/l) | Number | 11 | (0.7) | 6 | (0.7) |
| S_HDL_T7^1^ | Serum High density lipoprotein cholesterol (mmol/l) | Number | 11 | (0.7) | 6 | (0.7) |
| S_CHOLESTEROL_T7^1^ | Serum Total cholesterol (mmol/l) | Number | 11 | (0.7) | 6 | (0.7) |
| S_TRIGLYCERIDES_T7 | Serum Triglycerides (mmol/l) | Number | 11 | (0.7) | 6 | (0.7) |
| S_GLUCOSE_T7 | Serum glucose (mmol/l) | Number | 9 | (0.6) | 4 | (0.5) |
| S_CREATININ_T7 | Serum creatinin (µmol/L) | Number | 9 | (0.6) | 4 | (0.5) |
| B_HBA1C_T7 | HBA1C (%) | Number | 22 | (1.5) | 11 | (1.3) |
| HEALTH_T7 | How do you in general consider your own health to be? | 5 : Excellent  4 : Good  3 : Neither good nor bad  2 : Bad  1 : Very bad | 23 | (1.6) | 7 | (0.8) |
| HEALTH_COMPARED_T7 | How is your health now compared to others of your age? | 5 : Much better  4 : A little better  3 : About the same  2 : A little worse  1 : Much worse | 34 | (2.3) | 16 | (1.9) |
| HIGH_BLOOD_PRESSURE_T7 | Have you ever had, or do you have high blood pressure? | 0 : No  1 : Yes, now  2 : Yes, previously | 76 | (5.1) | 0 | (0.0) |
| HEART_ATTACK_T7 | Do you have, or have you had a heart attack? | 0 : No  2 : Yes, previously | 97 | (6.5) | 48 | (5.8) |
| HEART_FAILURE_T7 | Have you ever had, or do you have heart failure? | 0 : No  1 : Yes, now  2 : Yes, previously | 182 | (12.3) | 95 | (11.5) |
| ATRIAL_FIBRILLATION_T7 | Have you ever had, or do you have atrial fibrillation? | 0 : No  1 : Yes, now  2 : Yes, previously | 157 | (10.6) | 82 | (9.9) |
| ANGINA_T7 | Do you have, or have you had angina pectoris (heart cramp)? | 0 : No  1 : Yes, now  2 : Yes, previously | 130 | (8.8) | 66 | (8.0) |
| STROKE_T7 | Do you have, or have you had a cerebral stroke/brain haemorrhage? | 0 : No  2 : Yes, previously | 112 | (7.6) | 50 | (6.0) |
| KIDNEY_DISEASE_T7 | Do you have, or have you had a kidney disease, not including urinary tract infection (UTI)? | 0 : No  1 : Yes, now  2 : Yes, previously | 122 | (8.2) | 59 | (7.1) |
| BRONCHITIS_T7 | Have you ever had, or do you have chronic bronchitis/emphysema/COPD? | 0 : No  1 : Yes, now  2 : Yes, previously | 106 | (7.1) | 54 | (6.5) |
| ASTHMA_T7 | Do you have, or have you had asthma? | 0 : No  1 : Yes, now  2 : Yes, previously | 98 | (6.6) | 49 | (5.9) |
| CANCER_T7 | Have you ever had, or do you have cancer? | 0 : No  1 : Yes, now  2 : Yes, previously | 94 | (6.3) | 45 | (5.4) |
| RHEUMATOID_ARTHRITIS_T7 | Have you ever had, or do you have rheumatoid arthritis? | 0 : No  1 : Yes, now  2 : Yes, previously | 125 | (8.4) | 68 | (8.2) |
| ARTHROSIS_T7 | Have you ever had, or do you have arthrosis? | 0 : No  1 : Yes, now  2 : Yes, previously | 124 | (8.4) | 61 | (7.4) |
| MIGRAINE_T7 | Have you ever had, or do you still have migraine? | 0 : No  1 : Yes, now  2 : Yes, previously | 103 | (6.9) | 55 | (6.7) |
| PSYCHOLOGICAL_PROBLEMS_T7 | Have you ever had, or do you have psychological problems for which you have sought help? | 0 : No  1 : Yes, now  2 : Yes, previously | 115 | (7.8) | 57 | (6.9) |
| CHRONIC_PAIN_T7 | Do you have persistent or constantly recurring pain that has lasted for 3 months or more? | 0 : No  1 : Yes | 282 | (19.0) | 157 | (19.0) |
| CONSULTATION_PSYC_T7 | Have you during the past year visited a psychologist or psychiatrist? | 0 : No  1 : Yes | 105 | (7.1) | 45 | (5.4) |
| CONSULTATION_DENTIST_T7 | Have you during the past year visited a dentist/dental service? | 0 : No  1 : Yes | 49 | (3.3) | 20 | (2.4) |
| CONSULTATION_PHARMACY_T7 | Have you during the past year visited a pharmacy (to buy/get advice about medicines/treatment)? | 0 : No  1 : Yes | 87 | (5.9) | 38 | (4.6) |
| CONS_PHYSIOTHERAPIST_T7 | Have you during the past year visited a physiotherapist? | 0 : No  1 : Yes | 82 | (5.5) | 36 | (4.4) |
| CONSULTATION_CHIROPRACTOR_T7 | Have you during the past year visited a chiropractor? | 0 : No  1 : Yes | 89 | (6.0) | 39 | (4.7) |
| COMM_HEALTH_INTERNET_T7 | Have you during the past year communicated with any of the services above by using the Internet? | 0 : No  1 : Yes | 86 | (5.8) | 37 | (4.5) |
| CONSULTATION_HOSPITAL_PSYC_T7 | Have you during the past year visited a hospital out-patient clinic, psychiatric department? | 0 : No  1 : Yes | 193 | (13.0) | 97 | (11.7) |
| BP_TREATMENT_T7 | Do you use, or have you used blood pressure lowering drugs? | 1 : Currently  2 : Previously, not now  3 : Never used | 72 | (4.9) | 11 | (1.3) |
| DIURETICS_T7 | Do you use, or have you used diuretics? | 1 : Currently  2 : Previously, not now  3 : Never used | 145 | (9.8) | 78 | (9.4) |
| HEART_DISEASE_MEDICINE_T7 | Do you use, or have you used drugs for heart disease (for example anticoagulants, antiarrhythmics, nitroglycerin)? | 1 : Currently  2 : Previously, not now  3 : Never used | 86 | (5.8) | 37 | (4.5) |
| INSULIN_T7 | Do you use, or have you used insulin? | 1 : Currently  2 : Previously, not now  3 : Never used | 142 | (9.6) | 68 | (8.2) |
| DIABETES_TABLETS_T7 | Do you use, or have you used tablets for diabetes? | 1 : Currently  2 : Previously, not now  3 : Never used | 111 | (7.5) | 57 | (6.9) |
| THYROXINE_T7 | Do you use, or have you used drugs for hypothyroidism (Levaxin or thyroxine)? | 1 : Currently  2 : Previously, not now  3 : Never used | 122 | (8.2) | 62 | (7.5) |
| PAINKILLERS_PRESC_4WEEKS_T7 | How often have you used painkillers with prescription during the last 4 weeks? | 1 : Not used  2 : Less frequently than every week  3 : Every week, but not daily  4 : Daily | 103 | (6.9) | 54 | (6.5) |
| PAINKILLERS_NOPRESC_4WEEKS_T7 | How often have you used painkillers without prescription during the last 4 weeks? | 1 : Not used  2 : Less frequently than every week  3 : Every week, but not daily  4 : Daily | 100 | (6.7) | 54 | (6.5) |
| ACID_SUPRESSIVES_4WEEKS_T7 | How often have you used acid suppressive medication during the last 4 weeks? | 1 : Not used  2 : Less frequently than every week  3 : Every week, but not daily  4 : Daily | 121 | (8.2) | 62 | (7.5) |
| SLEEPING_PILLS_4WEEKS_T7 | How often have you used sleeping pills during the last 4 weeks? | 1 : Not used  2 : Less frequently than every week  3 : Every week, but not daily  4 : Daily | 112 | (7.6) | 56 | (6.8) |
| TRANQUILIZERS_4WEEKS_T7 | How often have you used tranquilizers during the last 4 weeks? | 1 : Not used  2 : Less frequently than every week  3 : Every week, but not daily  4 : Daily | 140 | (9.4) | 74 | (8.9) |
| ANTIDEPRESSANTS_4WEEKS_T7 | How often have you used antidepressants during the last 4 weeks? | 1 : Not used  2 : Less frequently than every week  3 : Every week, but not daily  4 : Daily | 161 | (10.9) | 91 | (11.0) |
| FRUIT_UNITS_T7 | How many units of fruit or vegetables do you eat per day (average). (E.g. an apple, bowl of salad)? | Number | 76 | (5.1) | 41 | (5.0) |
| RED_MEAT_T7 | How often do you usually eat red meat (all products from beef, mutton, pork)? | 1 : 0-1 times per month  2 : 2-3 times per month  3 : 1-3 times per week  4 : 4-6 times per week  5 : Once a day or more | 42 | (2.8) | 20 | (2.4) |
| FRUITS_VEG_BERRY_T7 | How often do you usually eat fruits, vegetables and berries? | 1 : 0-1 times per month  2 : 2-3 times per month  3 : 1-3 times per week  4 : 4-6 times per week  5 : Once a day or more | 31 | (2.1) | 13 | (1.6) |
| LEAN_FISH_T7 | How often do you usually eat lean fish (cod, saithe)? | 1 : 0-1 times per month  2 : 2-3 times per month  3 : 1-3 times per week  4 : 4-6 times per week  5 : Once a day or more | 32 | (2.2) | 17 | (2.1) |
| FAT_FISH_T7 | How often do you usually eat fat fish (salmon, trout, redfish, mackerel, herring, halibut)? | 1 : 0-1 times per month  2 : 2-3 times per month  3 : 1-3 times per week  4 : 4-6 times per week  5 : Once a day or more | 37 | (2.5) | 18 | (2.2) |
| PHYS_ACTIVITY_LEISURE_T7 | Describe your exercise and physical exertion in leisure time over the last year. If your activity varies throughout the year, give an average. | 1 : Reading, watching TV/screen or other sedentary activity?  2 : Walking, cycling, or other forms of exercise at least 4 hours a week? (including walking or cycling to place of work, Sunday-walking, etc.)  3 : Participation in recreational sports, heavy gardening, snow shoveling etc at least 4 hours a week.  4 : Participation in hard training or sports competitions, regularly several times a week? | 112 | (7.6) | 54 | (6.5) |
| HOURS_SITTING_WEEKDAY_T7 | During the last week, how much time did you spend sitting on a typical weekday? E.g. at at desk, while visiting friends, while watching TV/screen (including both work and leisure time) | Number | 326 | (22.0) | 181 | (21.9) |
| HOURS_SITTING_WEEKEND_T7 | During the last week, how much time did you spend sitting on a typical weekend day? E.g. at a desk, while visiting friends, while watching TV/screen | Number | 376 | (25.4) | 211 | (25.5) |
| EDUCATION_T7 | What is the highest levels of education you have completed? | 1 : Primary/partly secondary education. (Up to 10 years of schooling)  2 : Upper secondary education: (a minimum of 3 years)  3 : Tertiary education, short: College/university less than 4 years  4 : Tertiary education, long: College/university 4 years or more | 64 | (4.3) | 31 | (3.7) |
| LIVE_WITH_SPOUSE_T7 | Do you live with a spouse/partner? | 0 : No  1 : Yes | 105 | (7.1) | 53 | (6.4) |
| LIVE_WITH_O18_T7 | Do you live with persons older than 18 years of age other than your spouse/partner? | 0 : No  1 : Yes | 500 | (33.7) | 266 | (32.2) |
| LIVE_WITH_Y18_T7 | Do you live with persons younger than 18 years of age? | 0 : No  1 : Yes | 541 | (36.5) | 295 | (35.7) |
| SUPPORT_FRIENDS_T7 | Do you have enough friends who can give you help and support when you need it? | 0 : No  1 : Yes | 50 | (3.4) | 23 | (2.8) |
| HEALTH_SCALE_T7 | We would like to know how good or bad your health is today. This scale is numbered from 0-100. 100 means the best health you can imagine. 0 means the worst health you can imagine. Please insert a number between 0 and 100 here. | Number | 40 | (2.7) | 20 | (2.4) |
| BIOLOGICAL_CHILD_NUMBER_T7 | How many children do you have? Number of biological children. | Number | 73 | (4.9) | 37 | (4.5) |
| MOTHER_ALIVE_T7 | Is your mother alive? | 0 : No  1 : Yes | 29 | (2.0) | 11 | (1.3) |
| MOTHER_AGE_DEAD_T7 | If your mother is dead, how old was she when she died? | Number | 320 | (21.6) | 160 | (19.3) |
| FATHER_ALIVE_T7 | Is your father alive? | 0 : No  1 : Yes | 36 | (2.4) | 17 | (2.1) |
| FATHER_AGE_DEAD_T7 | If your father is dead, how old was he when he died? | Number | 205 | (13.8) | 97 | (11.7) |
| ECONOMY_T7 | How would you evaluate your finances? | 1 : Very good  2 : Good  3 : Average  4 : Difficult  5 : Very difficult | 30 | (2.0) | 15 | (1.8) |
| OCCUPATION_T7 | What is your main occupation/activity? | 1 : Works full time  2 : Works part time  3 : Unemployed  4 : Housekeeping  5 : Retired  6 : Student/military service  7 : Disabilty benefit recipient/work assessment allowance  8 : Family income supplement | 29 | (2.0) | 14 | (1.7) |
| OCCUPATION_STATUS_T7 | I consider my occupation to have the following social status in the society: (if you are not currently employed, think about your latest occupation) | 1 : Very high social status  2 : Fairly high social status  3 : Neither high nor low status  4 : Fairly low status  5 : Very low status | 71 | (4.8) | 38 | (4.6) |
| BYPASS_T7 | Have you had coronary artery bypass surgery? | 0 : No  1 : Yes | 149 | (10.0) | 84 | (10.2) |
| PCI_T7 | Have you had percutaneous coronary intervention? | 0 : No  1 : Yes | 64 | (4.3) | 31 | (3.7) |
| CLAUDICATIO_T7 | Have you had claudicatio intermittens? | 0 : No  1 : Yes | 139 | (9.4) | 72 | (8.7) |
| MEMORY_DECLINED_T7 | Have your memory declined? | 0 : No  1 : Yes | 37 | (2.5) | 20 | (2.4) |
| MEMORY_PROBLEM_DAILY_T7 | If you have answered yes to one of the four first questions above (Have your memory declined? Do you often forget where you have placed your things? Do you have difficulties finding word in a casual conversation? Have you problems performing daily tasks you used to master? Have you been examined for memory problems?) Is this a problem in your daily life? | 0 : No  1 : Yes | 514 | (34.7) | 268 | (32.4) |
| ANALGESICS_ANTIINFLAM_T7 | Have you used analgesics and anti-inflammatory medication regularly in the past year? These include both over-the-counter and prescription only medicines. | 0 : No  1 : Yes | 37 | (2.5) | 22 | (2.7) |
| REG_MEDICINES_4WEEKS_T7 | Have you used medicines (non-prescription and prescription) regularly during the last 4 weeks? Do not include dietary supplements (vitamins, minerals, omega-3, herbs or other natural remedies) | 0 : No  1 : Yes | 43 | (2.9) | 25 | (3.0) |
| EXERCISE_T7 | How often do you exercise (i.e walking, skiing, swimming or training/sports)? | 1 : Never  2 : Less than once a week  3 : Once a week  4 : 2-3 times a week | 34 | (2.3) | 19 | (2.3) |
| EXERCISE_LEVEL_T7 | If you exercise - how hard do you exercise? | 1 : Easy - you do not become shortwinded or sweaty  2 : You become shortwinded and sweaty  3 : Hard - you become exhausted | 205 | (13.8) | 115 | (13.9) |
| EXERCISE_DURATION_T7 | For how long time do you exercise? (give an average) | 1 : Less than 15 minutes  2 : 15-29 minutes  3 : 30-60 minutes  4 : More than 1 hour | 199 | (13.4) | 116 | (14.0) |
| ALCOHOL_STOP_DRINKING_T7 | How often during the last year have you: Not been able to stop drinking alcohol when first started? | 1 : Never  2 : Less than monthly  3 : Monthly  4 : Weekly  5 : Daily or almost daily | 257 | (17.3) | 142 | (17.2) |
| ALCOHOL_FAILED_EXPECTED_T7 | How often during the last year have you: Failed to do what was normally expected from you because of drinking? | 1 : Never  2 : Less than monthly  3 : Monthly  4 : Weekly  5 : Daily or almost daily | 263 | (17.7) | 145 | (17.5) |
| ALCOHOL_MORNING_T7 | How often during the last year have you: Needed alcohol in the morning to get yourself going after a heavy drinking session? | 1 : Never  2 : Less than monthly  3 : Monthly  4 : Weekly  5 : Daily or almost daily | 260 | (17.5) | 143 | (17.3) |
| ALCOHOL_REMORSE_T7 | How often during the last year have you: Had a feeling of guilt or remorse after drinking? | 1 : Never  2 : Less than monthly  3 : Monthly  4 : Weekly  5 : Daily or almost daily | 262 | (17.7) | 144 | (17.4) |
| ALCOHOL_NOT_REMEMBER_T7 | How often during the last year have you: Been unable to remember what happened the night before because you had been drinking? | 1 : Never  2 : Less than monthly  3 : Monthly  4 : Weekly  5 : Daily or almost daily | 270 | (18.2) | 148 | (17.9) |
| GET_DRUNK_FREQUENCY_T7 | Approximately how often during the past 12 months have you drunk so much that you felt highly intoxicated (drunk)? | 1 : Never  2 : Less than monthly  3 : Monthly  4 : Weekly  5 : Daily or almost daily | 263 | (17.7) | 145 | (17.5) |
| ALCOHOL_INJURED_T7 | How often during the last year have you or someone else been injured because of your drinking? | 1 : Never  2 : Yes, but not during the last year  3 : Yes, during the last year | 49 | (3.3) | 22 | (2.7) |
| ALCOHOL_CONCERNED_T7 | How often during the last year has a relative or friend or a doctor or another health worker been concerned about your drinking or suggested you cut down? | 1 : Never  2 : Yes, but not during the last year  3 : Yes, during the last year | 68 | (4.6) | 31 | (3.7) |
| SMOKE_START_AGE_T7 | If you currently smoke, or have smoked before, how old were you when you began smoking daily? | Number | 498 | (33.6) | 295 | (35.7) |
| SMOKE_YEARS_T7 | If you currently smoke, or have smoked before, how many years in all have you smoked daily? | Number | 514 | (34.7) | 304 | (36.8) |
| SMOKE_STOP_TIME_T7 | If you previously smoked daily, how long is it since you stopped (years)? | Number | 692 | (46.7) | 383 | (46.3) |
| AGE | Number of years at attendance date | Number | 0 | (0.0) | 0 | (0.0) |
| SYSBP_mean^2,3^ | Mean systolic blood pressure | Number | 5 | (0.3) | 3 | (0.4) |
| DIABP_mean^2,3^ | Mean diastolic blood pressure | Number | 5 | (0.3) | 3 | (0.4) |
| BP_med^1^ | Use of antihypertensive drugs | 0 : No  1 : Yes | 0 | (0.0) | 0 | (0.0) |
| Chol_med^2^ | Use of lipid lowering drugs | 0 : No  1 : Yes | 0 | (0.0) | 0 | (0.0) |
| CHD_BP_treatmentgoal^1^ | Mean blood pressure <140/90 mmHg | 0 : No  1 : Yes | 5 | (0.3) | 3 | (0.4) |
| CHD_LDL_treatmentgoal^2^ | LDL-cholesterol <1.8 mmol/l | 0 : No  1 : Yes | 11 | (0.7) | 6 | (0.7) |
| BMI | Body mass index | Number | 8 | (0.5) | 2 | (0.2) |
| Smoke_impu^3^ | Do you/did you smoke daily/sometimes? | 1 : Now, daily  2 : Now, sometimes  3 : Previously  4 : Never | 0 | (0.0) | 0 | (0.0) |
| Snuff_chewing_tobacco_impu^3^ | Have you used or do you use snuff or chewing tobacco daily/sometimes? | 1 : Now, daily  2 : Now, sometimes  3 : Previously  4 : Never | 3 | (0.2) | 2 | (0.2) |
| Cod_liver_oil_omega3_impu^3^ | Do you use cod liver oil or cod liver oil capsules or Omega 3 capsules (fish oil, seal oil)? | 0 : No  1 : Sometimes  2 : Daily during the winter season  3 : Daily | 38 | (2.6) | 21 | (2.5) |
| Alcohol_frequency_impu^3^ | How often do you usually drink alcohol? | 1 : Never  2 : Monthly or less frequently  3 : 2-4 times a month  4 : 2-3 times a week  5 : 4 or more times a week | 1 | (0.1) | 1 | (0.1) |
| Alcohol_units_impu^3^ | How many units of alcohol (1 beer, glass of wine or drink) do you usually drink when you drink alcohol? | 1 : 1-2  2 : 3-4  3 : 5-6  4 : 7-9  5 : 10 or more | 6 | (0.4) | 5 | (0.6) |
| Alcohol_units_6_impu^3^ | How often do you drink 6 units alcohol or more in one occasion? | 1 : Never  2 : Less frequently than monthly  3 : Monthly  4 : Weekly  5 : Daily or almost daily | 20 | (1.3) | 8 | (1.0) |
| Number_X_medicines | Number of medicines listed when asked to write down the medicines used regularly the last 4 weeks | Number | 0 | (0.0) | 0 | (0.0) |
| Cons_GP_Times_impu^3^ | If you have visited a general practitioner (GP) the past year, how many visits have you made? | Number | 188 | (12.7) | 96 | (11.6) |
| Cons_Emergency_Times_impu^3^ | If you have visited an emergency room the past year, how many visits have you made? | Number | 123 | (8.3) | 58 | (7.0) |
| Cons_Hospital_Times_impu^3^ | If you have been admitted to a hospital the past year, how many times? | Number | 64 | (4.3) | 29 | (3.5) |
| Cons_Spesialist_Times_impu^3^ | If you have visited another medical specialist than a general practitioner (GP) or a psychologist or psychiatrist (not at a hospital) the past year, how many visits have you made? | Number | 148 | (10.0) | 69 | (8.3) |
| Cons_Clinic_Times_impu^3^ | Have you during the past year visited a hospital out-patient clinic, other than psychiatric department? | Number | 139 | (9.4) | 64 | (7.7) |
| DIABETES_impu_new^3^ | Do you have, or have you had diabetes? | 0 : No  1 : Yes, now  2 : Yes, previously | 98 | (6.6) | 52 | (6.3) |
| ^1^Only used for analysis of blood pressure  ^2^Only used for analysis of cholesterol  ^3^Combination of several variables from Tromsø 7 | | |  |  |  |  |

Additional file: Table S4. Achievement of treatment goal for HbA1c for participants with diabetes in the different CHD disease groups

|  | Diabetic population, n | Achieved treatment goal for HbA1c,  n (%) | |
| --- | --- | --- | --- |
| Coronary heart disease | 214 | 91 | (42.5) |
| Myocardial infarction | 123 | 50 | (40.7) |
| Percutaneous coronary intervention/ Coronary artery bypass surgery | 80 | 34 | (42.5) |
| Angina pectoris | 11 | 7 | (63.6) |
|  | | | |

Additional file: Table S5. Results from propensity score matching of the ten imputed datasets for the logistic regression analysis of the association between use of lipid-lowering drugs and achieving the treatment goal for LDL-cholesterol

| **Dataset 1** | | | | | | | |
| --- | --- | --- | --- | --- | --- | --- | --- |
|  | | **Before matching** | | | **After matching** | | |
|  | | Not LLD user | LLD user | SMD | Not LLD user | LLD user | SMD |
| n | | 345 | 1127 |  | 252 | 1126 |  |
| AGE, mean (SD) | | 66.34 (13.45) | 69.41 (9.72) | 0.261 | 67.89 (13.15) | 69.40 (9.72) | 0.130 |
| SEX_T7, n (%) | |  |  | 0.390 |  |  | 0.209 |
|  | 1 | 193 (55.9) | 836 (74.2) |  | 163 (64.7) | 836 (74.2) |  |
| BMI, mean (SD) | | 28.26 (4.92) | 28.41 (4.35) | 0.033 | 28.36 (4.50) | 28.41 (4.35) | 0.011 |
| HEART_FAILURE_T7, n (%) | |  |  | 0.197 |  |  | 0.145 |
|  | 0 | 283 (82.0) | 841 (74.6) |  | 202 (80.2) | 840 (74.6) |  |
|  | 1 | 30 (8.7) | 114 (10.1) |  | 23 (9.1) | 114 (10.1) |  |
|  | 2 | 32 (9.3) | 172 (15.3) |  | 27 (10.7) | 172 (15.3) |  |
| ATRIAL_FIBRILLATION_T7, n (%) | |  |  | 0.142 |  |  | 0.067 |
|  | 0 | 236 (68.4) | 842 (74.7) |  | 181 (71.8) | 842 (74.8) |  |
|  | 1 | 65 (18.8) | 164 (14.6) |  | 41 (16.3) | 163 (14.5) |  |
|  | 2 | 44 (12.8) | 121 (10.7) |  | 30 (11.9) | 121 (10.7) |  |
| STROKE_T7, n (%) | |  |  | 0.074 |  |  | 0.038 |
|  | 2 | 41 (11.9) | 162 (14.4) |  | 33 (13.1) | 162 (14.4) |  |
| KIDNEY_DISEASE_T7, n (%) | |  |  | 0.137 |  |  | 0.128 |
|  | 0 | 282 (81.7) | 977 (86.7) |  | 207 (82.1) | 977 (86.8)" |  |
|  | 1 | 35 (10.1) | 87 (7.7) |  | 26 (10.3) | 86 (7.6) |  |
|  | 2 | 28 (8.1) | 63 (5.6) |  | 19 (7.5) | 63 (5.6) |  |
| CANCER_T7, n (%) | |  |  | 0.022 |  |  | 0.050 |
|  | 0 | 279 (80.9) | 920 (81.6) |  | 201 (79.8) | 919 (81.6) |  |
|  | 1 | 24 (7.0) | 73 (6.5) |  | 17 (6.7) | 73 (6.5) |  |
|  | 2 | 42 (12.2) | 134 (11.9) |  | 34 (13.5) | 134 (11.9) |  |
| DIABETES_impu_new, n (%) | |  |  | 0.096 |  |  | 0.064 |
|  | 0 | 274 (79.4) | 889 (78.9) |  | 200 (79.4) | 888 (78.9) |  |
|  | 1 | 51 (14.8) | 192 (17.0) |  | 39 (15.5) | 192 (17.1) |  |
|  | 2 | 20 (5.8) | 46 (4.1) |  | 13 (5.2) | 46 (4.1) |  |
| Cons_GP_Times_impu, mean (SD) | | 4.44 (6.03) | 4.25 (5.31) | 0.035 | 4.33 (5.90) | 4.19 (5.01) | 0.025 |
| Cons_Emergency_Times_impu, mean (SD) | | 0.44 (0.88) | 0.31 (0.74) | 0.165 | 0.37 (0.76) | 0.31 (0.74) | 0.083 |
| Cons_Hospital_Times_impu, mean (SD) | | 0.43 (1.26) | 0.43 (0.90) | 0.009 | 0.43 (1.35) | 0.42 (0.90) | 0.005 |
| Cons_Specialist_Times_impu, mean (SD) | | 0.63 (1.85) | 0.47 (2.49) | 0.073 | 0.53 (1.67) | 0.47 (2.49) | 0.026 |
| Cons_Clinic_Times_impu, mean (SD) | | 1.74 (7.48) | 1.14 (4.74) | 0.095 | 1.60 (7.14) | 1.14 (4.75) | 0.075 |
| Alcohol_frequency_impu, n (%) | |  |  | 0.150 |  |  | 0.142 |
|  | 1 | 34 (9.9) | 121 (10.7) |  | 23 (9.1) | 121 (10.7) |  |
|  | 2 | 85 (24.6) | 209 (18.5) |  | 60 (23.8) | 208 (18.5) |  |
|  | 3 | 129 (37.4) | 462 (41.0) |  | 94 (37.3) | 462 (41.0) |  |
|  | 4 | 69 (20.0) | 243 (21.6) |  | 53 (21.0) | 243 (21.6) |  |
|  | 5 | 28 (8.1) | 92 (8.2) |  | 22 (8.7) | 92 (8.2) |  |
| Alcohol_units_impu, n (%) | |  |  | 0.069 |  |  | 0.071 |
|  | 0 | 36 (10.4) | 119 (10.6) |  | 23 (9.1) | 119 (10.6) |  |
|  | 1 | 174 (50.4) | 542 (48.1) |  | 126 (50.0) | 541 (48.0) |  |
|  | 2 | 102 (29.6) | 367 (32.6) |  | 78 (31.0) | 367 (32.6) |  |
|  | 3 | 33 (9.6) | 99 (8.8) |  | 25 (9.9) | 99 (8.8) |  |
| Alcohol_units_6_impu, n (%) | |  |  | 0.117 |  |  | 0.146 |
|  | 1 | 188 (54.5) | 662 (58.7) |  | 133 (52.8) | 662 (58.8) |  |
|  | 2 | 116 (33.6) | 364 (32.3) |  | 89 (35.3) | 363 (32.2) |  |
|  | 3 | 32 (9.3) | 77 (6.8) |  | 23 (9.1) | 77 (6.8) |  |
|  | 4 | 7 (2.0) | 21 (1.9) |  | 5 (2.0) | 21 (1.9) |  |
|  | 5 | 2 (0.6) | 3 (0.3) |  | 2 (0.8) | 3 (0.3) |  |
| Smoke_impu, n (%) | |  |  | 0.305 |  |  | 0.245 |
|  | 1 | 57 (16.5) | 139 (12.3) |  | 43 (17.1) | 139 (12.3) |  |
|  | 2 | 12 (3.5) | 19 (1.7) |  | 7 (2.8) | 19 (1.7) |  |
|  | 3 | 167 (48.4) | 711 (63.1) |  | 129 (51.2) | 710 (63.1) |  |
|  | 4 | 109 (31.6) | 258 (22.9) |  | 73 (29.0) | 258 (22.9) |  |
| Snuff_chewing_tobacco_impu, n (%) | |  |  | 0.037 |  |  | 0.053 |
|  | 1 | 22 (6.4) | 67 (5.9) |  | 17 (6.7) | 67 (6.0) |  |
|  | 2 | 1 (0.3) | 2 (0.2) |  | 1 (0.4) | 2 (0.2) |  |
|  | 3 | 19 (5.5) | 57 (5.1) |  | 13 (5.2) | 57 (5.1) |  |
|  | 4 | 303 (87.8) | 1001 (88.8) |  | 221 (87.7) | 1000 (88.8) |  |
| Cod_liver_oil_omega3_impu, n (%) | |  |  | 0.100 |  |  | 0.089 |
|  | 0 | 261 (75.7) | 824 (73.1) |  | 189 (75.0) | 823 (73.1) |  |
|  | 1 | 44 (12.8) | 144 (12.8) |  | 34 (13.5) | 144 (12.8) |  |
|  | 2 | 15 (4.3) | 46 (4.1) |  | 10 (4.0) | 46 (4.1) |  |
|  | 3 | 25 (7.2) | 113 (10.0) |  | 19 (7.5) | 113 (10.0) |  |
| FRUIT_UNITS_T7, mean (SD) | | 1.72 (1.35) | 1.96 (1.71) | 0.157 | 1.73 (1.39) | 1.92 (1.28) | 0.143 |
| RED_MEAT_T7, n (%) | |  |  | 0.066 |  |  | 0.095 |
|  | 1 | 24 (7.0) | 74 (6.6) |  | 14 (5.6) | 74 (6.6) |  |
|  | 2 | 76 (22.0) | 250 (22.2) |  | 54 (21.4) | 250 (22.2) |  |
|  | 3 | 216 (62.6) | 723 (64.2) |  | 167 (66.3) | 722 (64.1) |  |
|  | 4 | 26 (7.5) | 68 (6.0) |  | 16 (6.3) | 68 (6.0) |  |
|  | 5 | 3 (0.9) | 12 (1.1) |  | 1 (0.4) | 12 (1.1) |  |
| FRUITS_VEG_BERRY_T7, n (%) | |  |  | 0.095 |  |  | 0.110 |
|  | 1 | 4 (1.2) | 12 (1.1) |  | 1 (0.4) | 12 (1.1) |  |
|  | 2 | 15 (4.3) | 45 (4.0) |  | 10 (4.0) | 45 (4.0) |  |
|  | 3 | 66 (19.1) | 259 (23.0) |  | 54 (21.4) | 259 (23.0) |  |
|  | 4 | 101 (29.3) | 313 (27.8) |  | 79 (31.3) | 312 (27.7) |  |
|  | 5 | 159 (46.1) | 498 (44.2)" |  | 108 (42.9) | 498 (44.2) |  |
| LEAN_FISH_T7, n (%) | |  |  | 0.231 |  |  | 0.102 |
|  | 1 | 24 (7.0) | 28 (2.5) |  | 10 (4.0) | 28 (2.5) |  |
|  | 2 | 50 (14.5) | 153 (13.6) |  | 38 (15.1) | 153 (13.6) |  |
|  | 3 | 224 (64.9) | 801 (71.1) |  | 175 (69.4) | 800 (71.0) |  |
|  | 4 | 41 (11.9) | 134 (11.9) |  | 27 (10.7) | 134 (11.9) |  |
|  | 5 | 6 (1.7) | 11 (1.0) |  | 2 (0.8) | 11 (1.0) |  |
| FAT_FISH_T7, n (%) | |  |  | 0.151 |  |  | 0.098 |
|  | 1 | 49 (14.2) | 113 (10.0) |  | 31 (12.3) | 112 (9.9) |  |
|  | 2 | 116 (33.6) | 392 (34.8) |  | 81 (32.1) | 392 (34.8) |  |
|  | 3 | 153 (44.3) | 550 (48.8) |  | 125 (49.6) | 550 (48.8) |  |
|  | 4 | 19 (5.5) | 54 (4.8) |  | 10 (4.0) | 54 (4.8) |  |
|  | 5 | 8 (2.3) | 18 (1.6) |  | 5 (2.0) | 18 (1.6) |  |
| PHYS_ACTIVITY_LEISURE_T7, n (%) | |  |  | 0.108 |  |  | 0.048 |
|  | 1 | 86 (24.9) | 232 (20.6) |  | 55 (21.8) | 231 (20.5) |  |
|  | 2 | 179 (51.9) | 625 (55.5) |  | 140 (55.6) | 625 (55.5) |  |
|  | 3 | 72 (20.9) | 247 (21.9) |  | 53 (21.0) | 247 (21.9) |  |
|  | 4 | 8 (2.3) | 23 (2.0) |  | 4 (1.6) | 23 (2.0) |  |
| EXERCISE_T7, n (%) | |  |  | 0.175 |  |  | 0.144 |
|  | 1 | 45 (13.0) | 107 (9.5) |  | 29 (11.5) | 107 (9.5) |  |
|  | 2 | 54 (15.7) | 142 (12.6) |  | 39 (15.5) | 142 (12.6) |  |
|  | 3 | 51 (14.8) | 155 (13.8) |  | 38 (15.1) | 155 (13.8) |  |
|  | 4 | 111 (32.2) | 434 (38.5) |  | 82 (32.5) | 433 (38.5) |  |
|  | 5 | 84 (24.3) | 289 (25.6) |  | 64 (25.4) | 289 (25.7) |  |
| **Dataset 2** | | | | | | | |
|  | | **Before matching** | | | **After matching** | | |
|  | | Not LLD user | LLD user | SMD | Not LLD user | LLD user | SMD |
| n | | 345 | 1127 |  | 261 | 1124 |  |
| AGE, mean (SD) | | 66.34 (13.45) | 69.41 (9.72) | 0.261 | 67.75 (12.84) | 69.38 (9.72) | 0.143 |
| SEX_T7, n (%) | |  |  | 0.390 |  |  | 0.247 |
|  | 1 | 193 (55.9) | 836 (74.2) |  | 164 (62.8) | 834 (74.2) |  |
| BMI, mean (SD) | | 28.27 (4.92) | 28.43 (4.41) | 0.036 | 28.52 (4.91) | 28.42 (4.40) | 0.021 |
| HEART_FAILURE_T7, n (%) | |  |  | 0.182 |  |  | 0.147 |
|  | 0 | 282 (81.7) | 838 (74.4) |  | 210 (80.5) | 837 (74.5) |  |
|  | 1 | 25 (7.2) | 104 (9.2) |  | 20 (7.7) | 103 (9.2) |  |
|  | 2 | 38 (11.0) | 185 (16.4) |  | 31 (11.9) | 184 (16.4) |  |
| ATRIAL_FIBRILLATION_T7, n (%) | |  |  | 0.140 |  |  | 0.081 |
|  | 0 | 236 (68.4) | 840 (74.5) |  | 187 (71.6) | 839 (74.6) |  |
|  | 1 | 65 (18.8) | 162 (14.4) |  | 45 (17.2) | 161 (14.3) |  |
|  | 2 | 44 (12.8) | 125 (11.1) |  | 29 (11.1) | 124 (11.0)" |  |
| STROKE_T7, n (%) | |  |  | 0.069 |  |  | 0.062 |
|  | 2 | 43 (12.5) | 167 (14.8) |  | 33 (12.6) | 166 (14.8) |  |
| KIDNEY_DISEASE_T7, n (%) | |  |  | 0.173 |  |  | 0.155 |
|  | 0 | 278 (80.6) | 977 (86.7) |  | 213 (81.6) | 976 (86.8) |  |
|  | 1 | 40 (11.6) | 99 (8.8) |  | 28 (10.7) | 97 (8.6) |  |
|  | 2 | 27 (7.8) | 51 (4.5) |  | 20 (7.7) | 51 (4.5) |  |
| CANCER_T7, n (%) | |  |  | 0.039 |  |  | 0.083 |
|  | 0 | 277 (80.3) | 922 (81.8) |  | 206 (78.9) | 919 (81.8) |  |
|  | 1 | 28 (8.1) | 84 (7.5) |  | 20 (7.7) | 84 (7.5) |  |
|  | 2 | 40 (11.6) | 121 (10.7) |  | 35 (13.4) | 121 (10.8) |  |
| DIABETES_impu_new, n (%) | |  |  | 0.065 |  |  | 0.087 |
|  | 0 | 273 (79.1) | 887 (78.7) |  | 209 (80.1) | 886 (78.8) |  |
|  | 1 | 54 (15.7) | 194 (17.2) |  | 38 (14.6) | 192 (17.1) |  |
|  | 2 | 18 (5.2) | 46 (4.1) |  | 14 (5.4) | 46 (4.1) |  |
| Cons_GP_Times_impu, mean (SD) | | 3.99 (4.80) | 4.15 (5.05) | 0.034 | 3.98 (4.32) | 4.09 (4.73) | 0.026 |
| Cons_Emergency_Times_impu, mean (SD) | | 0.41 (0.89) | 0.29 (0.71) | 0.149 | 0.37 (0.79) | 0.29 (0.71) | 0.105 |
| Cons_Hospital_Times_impu, mean (SD) | | 0.42 (1.24) | 0.43 (0.90) | 0.010 | 0.41 (1.33) | 0.43 (0.90) | 0.012 |
| Cons_Specialist_Times_impu, mean (SD) | | 0.49 (1.39) | 0.46 (2.52) | 0.016 | 0.44 (1.18) | 0.46 (2.52) | 0.010 |
| Cons_Clinic_Times_impu, mean (SD) | | 1.45 (4.89) | 1.22 (4.78) | 0.048 | 1.32 (2.84) | 1.22 (4.78) | 0.025 |
| Alcohol_frequency_impu, n (%) | |  |  | 0.152 |  |  | 0.151 |
|  | 1 | 34 (9.9) | 121 (10.7) |  | 28 (10.7) | 121 (10.8) |  |
|  | 2 | 85 (24.6) | 209 (18.5) |  | 60 (23.0) | 208 (18.5) |  |
|  | 3 | 128 (37.1) | 462 (41.0) |  | 96 (36.8) | 461 (41.0) |  |
|  | 4 | 69 (20.0) | 243 (21.6) |  | 50 (19.2) | 243 (21.6) |  |
|  | 5 | 29 (8.4) | 92 (8.2) |  | 27 (10.3) | 91 (8.1) |  |
| Alcohol_units_impu, n (%) | |  |  | 0.074 |  |  | 0.078 |
|  | 0 | 36 (10.4) | 119 (10.6) |  | 30 (11.5) | 119 (10.6) |  |
|  | 1 | 176 (51.0) | 542 (48.1) |  | 130 (49.8) | 539 (48.0) |  |
|  | 2 | 101 (29.3) | 367 (32.6) |  | 76 (29.1) | 367 (32.7) |  |
|  | 3 | 32 (9.3) | 99 (8.8) |  | 25 (9.6) | 99 (8.8) |  |
| Alcohol_units_6_impu, n (%) | |  |  | 0.107 |  |  | 0.095 |
|  | 1 | 188 (54.5) | 662 (58.7) |  | 147 (56.3) | 661 (58.8) |  |
|  | 2 | 120 (34.8) | 362 (32.1) |  | 86 (33.0) | 360 (32.0) |  |
|  | 3 | 28 (8.1) | 75 (6.7) |  | 20 (7.7) | 75 (6.7) |  |
|  | 4 | 6 (1.7) | 23 (2.0) |  | 5 (1.9) | 23 (2.0) |  |
|  | 5 | 3 (0.9) | 5 (0.4) |  | 3 (1.1) | 5 (0.4) |  |
| Smoke_impu, n (%) | |  |  | 0.305 |  |  | 0.243 |
|  | 1 | 57 (16.5) | 139 (12.3) |  | 45 (17.2) | 139 (12.4) |  |
|  | 2 | 12 (3.5) | 19 (1.7) |  | 7 (2.7) | 19 (1.7) |  |
|  | 3 | 167 (48.4) | 711 (63.1) |  | 134 (51.3) | 709 (63.1) |  |
|  | 4 | 109 (31.6) | 258 (22.9 |  | 75 (28.7) | 257 (22.9) |  |
| Snuff_chewing_tobacco_impu, n (%) | |  |  | 0.053 |  |  | 0.062 |
|  | 1 | 22 (6.4) | 67 (5.9) |  | 16 (6.1) | 67 (6.0) |  |
|  | 2 | 1 (0.3) | 1 (0.1) |  | 1 (0.4) | 1 (0.1) |  |
|  | 3 | 19 (5.5) | 58 (5.1) |  | 14 (5.4) | 58 (5.2) |  |
|  | 4 | 303 (87.8) | 1001 (88.8) |  | 230 (88.1) | 998 (88.8) |  |
| Cod_liver_oil_omega3_impu, n (%) | |  |  | 0.099 |  |  | 0.045 |
|  | 0 | 261 (75.7) | 825 (73.2) |  | 195 (74.7) | 823 (73.2) |  |
|  | 1 | 44 (12.8) | 142 (12.6) |  | 33 (12.6) | 142 (12.6) |  |
|  | 2 | 15 (4.3) | 47 (4.2) |  | 10 (3.8) | 47 (4.2) |  |
|  | 3 | 25 (7.2) | 113 (10.0) |  | 23 (8.8) | 112 (10.0) |  |
| FRUIT_UNITS_T7, mean (SD) | | 1.80 (1.49) | 1.98 (1.72) | 0.112 | 1.82 (1.54) | 1.94 (1.26) | 0.085 |
| RED_MEAT_T7, n (%) | |  |  | 0.088 |  |  | 0.122 |
|  | 1 | 24 (7.0) | 72 (6.4) |  | 18 (6.9) | 72 (6.4) |  |
|  | 2 | 73 (21.2) | 253 (22.4) |  | 53 (20.3) | 253 (22.5) |  |
|  | 3 | 218 (63.2) | 721 (64.0) |  | 169 (64.8) | 719 (64.0) |  |
|  | 4 | 27 (7.8) | 67 (5.9) |  | 20 (7.7) | 67 (6.0) |  |
|  | 5 | 3 (0.9) | 14 (1.2) |  | 1 (0.4) | 13 (1.2) |  |
| FRUITS_VEG_BERRY_T7, n (%) | |  |  | 0.097 |  |  | 0.080 |
|  | 1 | 3 (0.9) | 12 (1.1) |  | 2 (0.8) | 12 (1.1) |  |
|  | 2 | 14 (4.1) | 49 (4.3) |  | 9 (3.4) | 49 (4.4) |  |
|  | 3 | 65 (18.8) | 253 (22.4) |  | 54 (20.7) | 253 (22.5) |  |
|  | 4 | 103 (29.9) | 313 (27.8) |  | 73 (28.0) | 312 (27.8) |  |
|  | 5 | 160 (46.4) | 500 (44.4) |  | 123 (47.1) | 498 (44.3) |  |
| LEAN_FISH_T7, n (%) | |  |  | 0.223 |  |  | 0.124 |
|  | 1 | 23 (6.7) | 28 (2.5) |  | 12 (4.6) | 28 (2.5) |  |
|  | 2 | 50 (14.5) | 151 (13.4) |  | 37 (14.2) | 151 (13.4) |  |
|  | 3 | 229 (66.4) | 802 (71.2) |  | 180 (69.0) | 800 (71.2) |  |
|  | 4 | 36 (10.4) | 133 (11.8) |  | 30 (11.5) | 132 (11.7) |  |
|  | 5 | 7 (2.0) | 13 (1.2) |  | 2 (0.8) | 13 (1.2) |  |
| FAT_FISH_T7, n (%) | |  |  | 0.156 |  |  | 0.065 |
|  | 1 | 51 (14.8) | 113 (10.0) |  | 29 (11.1) | 112 (10.0) |  |
|  | 2 | 116 (33.6) | 393 (34.9) |  | 88 (33.7) | 393 (35.0) |  |
|  | 3 | 156 (45.2) | 551 (48.9) |  | 130 (49.8) | 549 (48.8) |  |
|  | 4 | 15 (4.3) | 54 (4.8) |  | 10 (3.8) | 54 (4.8) |  |
|  | 5 | 7 (2.0) | 16 (1.4) |  | 4 (1.5) | 16 (1.4) |  |
| PHYS_ACTIVITY_LEISURE_T7, n (%) | |  |  | 0.069 |  |  | 0.050 |
|  | 1 | 79 (22.9) | 241 (21.4) |  | 57 (21.8) | 238 (21.2) |  |
|  | 2 | 182 (52.8) | 623 (55.3) |  | 139 (53.3) | 623 (55.4) |  |
|  | 3 | 75 (21.7) | 242 (21.5) |  | 59 (22.6) | 242 (21.5) |  |
|  | 4 | 9 (2.6) | 21 (1.9) |  | 6 (2.3) | 21 (1.9) |  |
| EXERCISE_T7, n (%) | |  |  | 0.176 |  |  | 0.127 |
|  | 1 | 43 (12.5) | 102 (9.1) |  | 26 (10.0) | 102 (9.1) |  |
|  | 2 | 54 (15.7) | 143 (12.7) |  | 41 (15.7) | 142 (12.6) |  |
|  | 3 | 51 (14.8) | 153 (13.6) |  | 36 (13.8) | 153 (13.6) |  |
|  | 4 | 111 (32.2) | 437 (38.8) |  | 87 (33.3) | 435 (38.7) |  |
|  | 5 | 86 (24.9) | 292 (25.9) |  | 71 (27.2) | 292 (26.0) |  |
| **Dataset 3** | | | | | | | |
|  | | **Before matching** | | | **After matching** | | |
|  | | Not LLD user | LLD user | SMD | Not LLD user | LLD user | SMD |
| n | | 345 | 1127 |  | 259 | 1127 |  |
| AGE, mean (SD) | | 66.34 (13.45) | 69.41 (9.72) | 0.261 | 68.00 (12.72) | 69.41 (9.72 | 0.124 |
| SEX_T7, n (%) | |  |  | 0.390 |  |  | 0.212 |
|  | 1 | 193 (55.9) | 836 (74.2) |  | 167 (64.5) | 836 (74.2) |  |
| BMI, mean (SD) | | 28.26 (4.94) | 28.41 (4.35) | 0.031 | 28.49 (4.70) | 28.41 (4.35) | 0.018 |
| HEART_FAILURE_T7, n (%) | |  |  | 0.191 |  |  | 0.190 |
|  | 0 | 279 (80.9) | 844 (74.9) |  | 213 (82.2) | 844 (74.9) |  |
|  | 1 | 33 (9.6) | 104 (9.2) |  | 20 (7.7) | 104 (9.2) |  |
|  | 2 | 33 (9.6) | 179 (15.9) |  | 26 (10.0) | 179 (15.9) |  |
| ATRIAL_FIBRILLATION_T7, n (%) | |  |  | 0.153 |  |  | 0.093 |
|  | 0 | 238 (69.0) | 844 (74.9) |  | 187 (72.2) | 844 (74.9) |  |
|  | 1 | 69 (20.0) | 162 (14.4) |  | 46 (17.8) | 162 (14.4) |  |
|  | 2 | 38 (11.0) | 121 (10.7) |  | 26 (10.0) | 121 (10.7) |  |
| STROKE_T7, n (%) | |  |  | 0.065 |  |  | 0.048 |
|  | 2 | 42 (12.2) | 162 (14.4) |  | 33 (12.7) | 162 (14.4) |  |
| KIDNEY_DISEASE_T7, n (%) | |  |  | 0.143 |  |  | 0.090 |
|  | 0 | 282 (81.7) | 976 (86.6) |  | 216 (83.4) | 976 (86.6) |  |
|  | 1 | 36 (10.4) | 96 (8.5) |  | 27 (10.4) | 96 (8.5) |  |
|  | 2 | 27 (7.8) | 55 (4.9) |  | 16 (6.2) | 55 (4.9) |  |
| CANCER_T7, n (%) | |  |  | 0.028 |  |  | 0.053 |
|  | 0 | 280 (81.2) | 919 (81.5) |  | 206 (79.5) | 919 (81.5) |  |
|  | 1 | 24 (7.0) | 83 (7.4) |  | 22 (8.5) | 83 (7.4) |  |
|  | 2 | 41 (11.9) | 125 (11.1) |  | 31 (12.0) | 125 (11.1) |  |
| DIABETES_impu_new, n (%) | |  |  | 0.114 |  |  | 0.106 |
|  | 0 | 274 (79.4) | 887 (78.7) |  | 208 (80.3) | 887 (78.7) |  |
|  | 1 | 52 (15.1) | 200 (17.7) |  | 38 (14.7) | 200 (17.7) |  |
|  | 2 | 19 (5.5) | 40 (3.5) |  | 13 (5.0) | 40 (3.5) |  |
| Cons_GP_Times_impu, mean (SD) | | 4.33 (6.06) | 4.14 (4.77) | 0.036 | 3.99 (5.30) | 4.14 (4.77) | 0.028 |
| Cons_Emergency_Times_impu, mean (SD) | | 0.39 (0.81) | 0.30 (0.73) | 0.116 | 0.31 (0.72) | 0.30 (0.73) | 0.008 |
| Cons_Hospital_Times_impu, mean (SD) | | 0.48 (1.43) | 0.43 (0.91) | 0.040 | 0.42 (1.26) | 0.43 (0.91) | 0.012 |
| Cons_Specialist_Times_impu, mean (SD) | | 0.61 (1.78) | 0.48 (2.56) | 0.059 | 0.62 (1.74) | 0.48 (2.56) | 0.061 |
| Cons_Clinic_Times_impu, mean (SD) | | 1.12 (2.48) | 1.07 (4.17) | 0.012 | 1.18 (2.61) | 1.07 (4.17) | 0.030 |
| Alcohol_frequency_impu, n (%) | |  |  | 0.152 |  |  | 0.146 |
|  | 1 | 34 (9.9) | 121 (10.7) |  | 23 (8.9) | 121 (10.7) |  |
|  | 2 | 85 (24.6) | 209 (18.5) |  | 62 (23.9) | 209 (18.5) |  |
|  | 3 | 128 (37.1) | 462 (41.0) |  | 101 (39.0) | 462 (41.0) |  |
|  | 4 | 69 (20.0) | 243 (21.6) |  | 50 (19.3) | 243 (21.6) |  |
|  | 5 | 29 (8.4) | 92 (8.2) |  | 23 (8.9) | 92 (8.2) |  |
| Alcohol_units_impu, n (%) | |  |  | 0.074 |  |  | 0.078 |
|  | 0 | 36 (10.4) | 119 (10.6) |  | 24 (9.3) | 119 (10.6) |  |
|  | 1 | 175 (50.7) | 541 (48.0) |  | 132 (51.0) | 541 (48.0) |  |
|  | 2 | 101 (29.3) | 367 (32.6) |  | 78 (30.1) | 367 (32.6) |  |
|  | 3 | 33 (9.6) | 100 (8.9) |  | 25 (9.7) | 100 (8.9) |  |
| Alcohol_units_6_impu, n (%) | |  |  | 0.129 |  |  | 0.126 |
|  | 1 | 187 (54.2) | 664 (58.9) |  | 139 (53.7) | 664 (58.9) |  |
|  | 2 | 121 (35.1) | 363 (32.2) |  | 93 (35.9) | 363 (32.2) |  |
|  | 3 | 28 (8.1) | 74 (6.6) |  | 20 (7.7) | 74 (6.6) |  |
|  | 4 | 6 (1.7) | 23 (2.0) |  | 5 (1.9) | 23 (2.0) |  |
|  | 5 | 3 (0.9) | 3 (0.3) |  | 2 (0.8) | 3 (0.3) |  |
| Smoke_impu, n (%) | |  |  | 0.305 |  |  | 0.195 |
|  | 1 | 57 (16.5) | 139 (12.3) |  | 37 (14.3) | 139 (12.3) |  |
|  | 2 | 12 (3.5) | 19 (1.7) |  | 8 (3.1) | 19 (1.7) |  |
|  | 3 | 167 (48.4) | 711 (63.1) |  | 140 (54.1) | 711 (63.1) |  |
|  | 4 | 109 (31.6) | 258 (22.9) |  | 74 (28.6) | 258 (22.9) |  |
| Snuff_chewing_tobacco_impu, n (%) | |  |  | 0.054 |  |  | 0.072 |
|  | 1 | 22 (6.4) | 67 (5.9) |  | 16 (6.2) | 67 (5.9) |  |
|  | 2 | 1 (0.3) | 1 (0.1) |  | 1 (0.4) | 1 (0.1) |  |
|  | 3 | 19 (5.5) | 57 (5.1) |  | 11 (4.2) | 57 (5.1) |  |
|  | 4 | 303 (87.8) | 1002 (88.9) |  | 231 (89.2) | 1002 (88.9) |  |
| Cod_liver_oil_omega3_impu, n (%) | |  |  | 0.109 |  |  | 0.106 |
|  | 0 | 259 (75.1) | 821 (72.8) |  | 196 (75.7) | 821 (72.8) |  |
|  | 1 | 46 (13.3) | 142 (12.6) |  | 34 (13.1) | 142 (12.6) |  |
|  | 2 | 14 (4.1) | 44 (3.9) |  | 9 (3.5) | 44 (3.9) |  |
|  | 3 | 26 (7.5) | 120 (10.6) |  | 20 (7.7) | 120 (10.6) |  |
| FRUIT_UNITS_T7, mean (SD) | | 1.72 (1.33) | 1.97 (1.71) | 0.164 | 1.76 (1.42) | 1.97 (1.71) | 0.131 |
| RED_MEAT_T7, n (%) | |  |  | 0.079 |  |  | 0.075 |
|  | 1 | 24 (7.0) | 75 (6.7) |  | 19 (7.3) | 75 (6.7) |  |
|  | 2 | 79 (22.9) | 256 (22.7) |  | 65 (25.1) | 256 (22.7) |  |
|  | 3 | 213 (61.7) | 718 (63.7) |  | 159 (61.4) | 718 (63.7) |  |
|  | 4 | 26 (7.5) | 65 (5.8) |  | 14 (5.4) | 65 (5.8) |  |
|  | 5 | 3 (0.9) | 13 (1.2) |  | 2 (0.8) | 13 (1.2) |  |
| FRUITS_VEG_BERRY_T7, n (%) | |  |  | 0.105 |  |  | 0.062 |
|  | 1 | 3 (0.9) | 12 (1.1) |  | 2 (0.8) | 12 (1.1) |  |
|  | 2 | 13 (3.8) | 45 (4.0) |  | 9 (3.5) | 45 (4.0) |  |
|  | 3 | 65 (18.8) | 258 (22.9) |  | 56 (21.6) | 258 (22.9) |  |
|  | 4 | 101 (29.3) | 312 (27.7) |  | 77 (29.7) | 312 (27.7) |  |
|  | 5 | 163 (47.2) | 500 (44.4) |  | 115 (44.4) | 500 (44.4) |  |
| LEAN_FISH_T7, n (%) | |  |  | 0.218 |  |  | 0.095 |
|  | 1 | 23 (6.7) | 28 (2.5) |  | 9 (3.5) | 28 (2.5) |  |
|  | 2 | 55 (15.9) | 155 (13.8) |  | 42 (16.2) | 155 (13.8) |  |
|  | 3 | 227 (65.8) | 799 (70.9) |  | 176 (68.0) | 799 (70.9) |  |
|  | 4 | 36 (10.4) | 133 (11.8) |  | 29 (11.2) | 133 (11.8) |  |
|  | 5 | 4 (1.2) | 12 (1.1) |  | 3 (1.2) | 12 (1.1) |  |
| FAT_FISH_T7, n (%) | |  |  | 0.145 |  |  | 0.135 |
|  | 1 | 52 (15.1) | 117 (10.4) |  | 38 (14.7) | 117 (10.4) |  |
|  | 2 | 116 (33.6) | 390 (34.6) |  | 86 (33.2) | 390 (34.6) |  |
|  | 3 | 157 (45.5) | 548 (48.6) |  | 121 (46.7) | 548 (48.6) |  |
|  | 4 | 14 (4.1) | 54 (4.8) |  | 10 (3.9) | 54 (4.8) |  |
|  | 5 | 6 (1.7) | 18 (1.6) |  | 4 (1.5) | 18 (1.6) |  |
| PHYS_ACTIVITY_LEISURE_T7, n (%) | |  |  | 0.078 |  |  | 0.081 |
|  | 1 | 83 (24.1) | 236 (20.9) |  | 63 (24.3) | 236 (20.9) |  |
|  | 2 | 180 (52.2) | 622 (55.2) |  | 136 (52.5) | 622 (55.2) |  |
|  | 3 | 75 (21.7) | 246 (21.8) |  | 55 (21.2) | 246 (21.8) |  |
|  | 4 | 7 (2.0) | 23 (2.0) |  | 5 (1.9) | 23 (2.0) |  |
| EXERCISE_T7, n (%) | |  |  | 0.179 |  |  | 0.177 |
|  | 1 | 45 (13.0) | 107 (9.5) |  | 31 (12.0) | 107 (9.5) |  |
|  | 2 | 53 (15.4) | 141 (12.5) |  | 40 (15.4) | 141 (12.5) |  |
|  | 3 | 52 (15.1) | 154 (13.7) |  | 39 (15.1) | 154 (13.7) |  |
|  | 4 | 110 (31.9) | 436 (38.7) |  | 80 (30.9) | 436 (38.7) |  |
|  | 5 | 85 (24.6) | 289 (25.6) |  | 69 (26.6) | 289 (25.6) |  |
| **Dataset 4** | | | | | | | |
|  | | **Before matching** | | | **After matching** | | |
|  | | Not LLD user | LLD user | SMD | Not LLD user | LLD user | SMD |
| n | | 345 | 1127 |  | 244 | 1126 |  |
| AGE, mean (SD) | | 66.34 (13.45) | 69.41 (9.72) | 0.261 | 68.00 (12.67) | 69.40 (9.72) | 0.124 |
| SEX_T7, n (%) | |  |  | 0.390 |  |  | 0.285 |
|  | 1 | 193 (55.9) | 836 (74.2) |  | 149 (61.1) | 836 (74.2) |  |
| BMI, mean (SD) | | 28.26 (4.94) | 28.43 (4.39) | 0.035 | 28.61 (4.82) | 28.42 (4.39) | 0.041 |
| HEART_FAILURE_T7, n (%) | |  |  | 0.212 |  |  | 0.150 |
|  | 0 | 280 (81.2) | 840 (74.5) |  | 197 (80.7) | 839 (74.5) |  |
|  | 1 | 32 (9.3) | 99 (8.8) |  | 16 (6.6) | 99 (8.8) |  |
|  | 2 | 33 (9.6) | 188 (16.7) |  | 31 (12.7) | 188 (16.7) |  |
| ATRIAL_FIBRILLATION_T7, n (%) | |  |  | 0.151 |  |  | 0.137 |
|  | 0 | 236 (68.4) | 843 (74.8) |  | 173 (70.9) | 843 (74.9) |  |
|  | 1 | 67 (19.4) | 161 (14.3) |  | 47 (19.3) | 160 (14.2) |  |
|  | 2 | 42 (12.2) | 123 (10.9) |  | 24 (9.8) | 123 (10.9) |  |
| STROKE_T7, n (%) | |  |  | 0.072 |  |  | 0.082 |
|  | 2 | 42 (12.2) | 165 (14.6) |  | 29 (11.9) | 165 (14.7) |  |
| KIDNEY_DISEASE_T7, n (%) | |  |  | 0.124 |  |  | 0.035 |
|  | 0 | 284 (82.3) | 978 (86.8) |  | 209 (85.7) | 978 (86.9) |  |
|  | 1 | 37 (10.7) | 90 (8.0) |  | 21 (8.6) | 89 (7.9) |  |
|  | 2 | 24 (7.0) | 59 (5.2) |  | 14 (5.7) | 59 (5.2) |  |
| CANCER_T7, n (%) | |  |  | 0.035 |  |  | 0.049 |
|  | 0 | 279 (80.9) | 919 (81.5) |  | 196 (80.3) | 918 (81.5) |  |
|  | 1 | 27 (7.8) | 78 (6.9) |  | 16 (6.6) | 78 (6.9) |  |
|  | 2 | 39 (11.3) | 130 (11.5) |  | 32 (13.1) | 130 (11.5) |  |
| DIABETES_impu_new, n (%) | |  |  | 0.064 |  |  | 0.056 |
|  | 0 | 276 (80.0) | 889 (78.9) |  | 195 (79.9) | 888 (78.9) |  |
|  | 1 | 53 (15.4) | 195 (17.3) |  | 38 (15.6) | 195 (17.3) |  |
|  | 2 | 16 (4.6) | 43 (3.8) |  | 11 (4.5) | 43 (3.8) |  |
| Cons_GP_Times_impu, mean (SD) | | 4.59 (6.65) | 4.18 (5.04) | 0.070 | 4.31 (6.35) | 4.17 (5.03) | 0.024 |
| Cons_Emergency_Times_impu, mean (SD) | | 0.39 (0.96) | 0.30 (0.73) | 0.104 | 0.31 (0.89) | 0.30 (0.73) | 0.011 |
| Cons_Hospital_Times_impu, mean (SD) | | 0.44 (1.25) | 0.43 (0.90) | 0.004 | 0.39 (1.33) | 0.43 (0.90) | 0.033 |
| Cons_Specialist_Times_impu, mean (SD) | | 0.54 (1.62) | 0.44 (2.44) | 0.051 | 0.48 (1.39) | 0.44 (2.44) | 0.018 |
| Cons_Clinic_Times_impu, mean (SD) | | 1.04 (2.38) | 1.09 (4.16) | 0.014 | 1.02 (2.49) | 1.09 (4.16) | 0.021 |
| Alcohol_frequency_impu, n (%) | |  |  | 0.150 |  |  | 0.133 |
|  | 1 | 34 (9.9) | 121 (10.7) |  | 25 (10.2) | 121 (10.7) |  |
|  | 2 | 85 (24.6) | 209 (18.5) |  | 58 (23.8) | 208 (18.5) |  |
|  | 3 | 129 (37.4) | 462 (41.0) |  | 94 (38.5) | 462 (41.0) |  |
|  | 4 | 69 (20.0) | 243 (21.6) |  | 47 (19.3) | 243 (21.6) |  |
|  | 5 | 28 (8.1) | 92 (8.2) |  | 20 (8.2) | 92 (8.2) |  |
| Alcohol_units_impu, n (%) | |  |  | 0.076 |  |  | 0.061 |
|  | 0 | 36 (10.4) | 121 (10.7) |  | 26 (10.7) | 121 (10.7) |  |
|  | 1 | 176 (51.0) | 540 (47.9) |  | 123 (50.4) | 539 (47.9) |  |
|  | 2 | 101 (29.3) | 367 (32.6) |  | 73 (29.9) | 367 (32.6) |  |
|  | 3 | 32 (9.3) | 99 (8.8) |  | 22 (9.0) | 99 (8.8) |  |
| Alcohol_units_6_impu, n (%) | |  |  | 0.103 |  |  | 0.065 |
|  | 1 | 187 (54.2) | 662 (58.7) |  | 137 (56.1) | 662 (58.8) |  |
|  | 2 | 122 (35.4) | 366 (32.5) |  | 84 (34.4) | 365 (32.4) |  |
|  | 3 | 27 (7.8) | 74 (6.6) |  | 16 (6.6) | 74 (6.6) |  |
|  | 4 | 7 (2.0) | 22 (2.0) |  | 6 (2.5) | 22 (2.0) |  |
|  | 5 | 2 (0.6) | 3 (0.3) |  | 1 (0.4) | 3 (0.3) |  |
| Smoke_impu, n (%) | |  |  | 0.305 |  |  | 0.271 |
|  | 1 | 57 (16.5) | 139 (12.3) |  | 39 (16.0) | 139 (12.3) |  |
|  | 2 | 12 (3.5) | 19 (1.7) |  | 4 (1.6) | 19 (1.7) |  |
|  | 3 | 167 (48.4) | 711 (63.1) |  | 122 (50.0) | 710 (63.1) |  |
|  | 4 | 109 (31.6) | 258 (22.9) |  | 79 (32.4) | 258 (22.9) |  |
| Snuff_chewing_tobacco_impu, n (%) | |  |  | 0.034 |  |  | 0.096 |
|  | 1 | 22 (6.4) | 67 (5.9) |  | 12 (4.9) | 67 (6.0) |  |
|  | 2 | 1 (0.3) | 2 (0.2) |  | 1 (0.4) | 2 (0.2) |  |
|  | 3 | 19 (5.5) | 58 (5.1) |  | 9 (3.7) | 58 (5.2) |  |
|  | 4 | 303 (87.8) | 1000 (88.7) |  | 222 (91.0) | 999 (88.7) |  |
| Cod_liver_oil_omega3_impu, n (%) | |  |  | 0.091 |  |  | 0.081 |
|  | 0 | 260 (75.4) | 825 (73.2) |  | 181 (74.2) | 824 (73.2) |  |
|  | 1 | 45 (13.0) | 145 (12.9) |  | 35 (14.3) | 145 (12.9) |  |
|  | 2 | 14 (4.1) | 43 (3.8) |  | 8 (3.3) | 43 (3.8) |  |
|  | 3 | 26 (7.5) | 114 (10.1) |  | 20 (8.2) | 114 (10.1) |  |
| FRUIT_UNITS_T7, mean (SD) | | 1.74 (1.35) | 1.95 (1.71) | 0.142 | 1.76 (1.28) | 1.92 (1.28) | 0.127 |
| RED_MEAT_T7, n (%) | |  |  | 0.068 |  |  | 0.090 |
|  | 1 | 24 (7.0) | 70 (6.2) |  | 14 (5.7) | 70 (6.2) |  |
|  | 2 | 78 (22.6) | 251 (22.3) |  | 57 (23.4) | 251 (22.3) |  |
|  | 3 | 214 (62.0) | 722 (64.1) |  | 156 (63.9) | 721 (64.0) |  |
|  | 4 | 26 (7.5) | 71 (6.3) |  | 16 (6.6) | 71 (6.3) |  |
|  | 5 | 3 (0.9) | 13 (1.2) |  | 1 (0.4) | 13 (1.2) |  |
| FRUITS_VEG_BERRY_T7, n (%) | |  |  | 0.124 |  |  | 0.091 |
|  | 1 | 5 (1.4) | 13 (1.2) |  | 4 (1.6) | 13 (1.2) |  |
|  | 2 | 14 (4.1) | 44 (3.9) |  | 11 (4.5) | 44 (3.9) |  |
|  | 3 | 62 (18.0) | 258 (22.9) |  | 48 (19.7) | 258 (22.9) |  |
|  | 4 | 101 (29.3) | 313 (27.8) |  | 71 (29.1) | 312 (27.7) |  |
|  | 5 | 163 (47.2) | 499 (44.3) |  | 110 (45.1) | 499 (44.3) |  |
| LEAN_FISH_T7, n (%) | |  |  | 0.217 |  |  | 0.101 |
|  | 1 | 23 (6.7) | 28 (2.5) |  | 9 (3.7) | 28 (2.5) |  |
|  | 2 | 53 (15.4) | 150 (13.3) |  | 37 (15.2) | 150 (13.3) |  |
|  | 3 | 224 (64.9) | 800 (71.0) |  | 166 (68.0) | 799 (71.0) |  |
|  | 4 | 41 (11.9) | 135 (12.0) |  | 30 (12.3) | 135 (12.0) |  |
|  | 5 | 4 (1.2) | 14 (1.2) |  | 2 (0.8) | 14 (1.2) |  |
| FAT_FISH_T7, n (%) | |  |  | 0.156 |  |  | 0.124 |
|  | 1 | 50 (14.5) | 114 (10.1) |  | 28 (11.5) | 113 (10.0) |  |
|  | 2 | 114 (33.0) | 389 (34.5) |  | 74 (30.3) | 389 (34.5) |  |
|  | 3 | 158 (45.8) | 553 (49.1) |  | 124 (50.8) | 553 (49.1) |  |
|  | 4 | 14 (4.1) | 53 (4.7) |  | 11 (4.5) | 53 (4.7) |  |
|  | 5 | 9 (2.6) | 18 (1.6) |  | 7 (2.9) | 18 (1.6) |  |
| PHYS_ACTIVITY_LEISURE_T7, n (%) | |  |  | 0.073 |  |  | 0.024 |
|  | 1 | 82 (23.8) | 234 (20.8) |  | 52 (21.3) | 233 (20.7) |  |
|  | 2 | 180 (52.2) | 615 (54.6) |  | 134 (54.9) | 615 (54.6) |  |
|  | 3 | 76 (22.0) | 253 (22.4) |  | 53 (21.7) | 253 (22.5) |  |
|  | 4 | 7 (2.0) | 25 (2.2) |  | 5 (2.0) | 25 (2.2) |  |
| EXERCISE_T7, n (%) | |  |  | 0.166 |  |  | 0.126 |
|  | 1 | 43 (12.5) | 104 (9.2) |  | 27 (11.1) | 104 (9.2) |  |
|  | 2 | 53 (15.4) | 143 (12.7) |  | 33 (13.5) | 143 (12.7) |  |
|  | 3 | 52 (15.1) | 156 (13.8) |  | 40 (16.4) | 156 (13.9) |  |
|  | 4 | 111 (32.2) | 434 (38.5) |  | 81 (33.2) | 433 (38.5) |  |
|  | 5 | 86 (24.9) | 290 (25.7) |  | 63 (25.8) | 290 (25.8) |  |
| **Dataset 5** | | | | | | | |
|  | | **Before matching** | | | **After matching** | | |
|  | | Not LLD user | LLD user | SMD | Not LLD user | LLD user | SMD |
| n | | 345 | 1127 |  | 249 | 1126 |  |
| AGE, mean (SD) | | 66.34 (13.45) | 69.41 (9.72) | 0.261 | 67.91 (12.69) | 69.40 (9.72) | 0.132 |
| SEX_T7, n (%) | |  |  | 0.390 |  |  | 0.243 |
|  | 1 | 193 (55.9) | 836 (74.2) |  | 157 (63.1) | 836 (74.2) |  |
| BMI, mean (SD) | | 28.23 (4.94) | 28.42 (4.37) | 0.042 | 28.31 (4.83) | 28.42 (4.37) | 0.024 |
| HEART_FAILURE_T7, n (%) | |  |  | 0.190 |  |  | 0.133 |
|  | 0 | 282 (81.7) | 838 (74.4) |  | 199 (79.9) | 837 (74.3) |  |
|  | 1 | 27 (7.8) | 104 (9.2) |  | 18 (7.2) | 104 (9.2) |  |
|  | 2 | 36 (10.4) | 185 (16.4) |  | 32 (12.9) | 185 (16.4) |  |
| ATRIAL_FIBRILLATION_T7, n (%) | |  |  | 0.123 |  |  | 0.125 |
|  | 0 | 239 (69.3) | 839 (74.4) |  | 174 (69.9) | 839 (74.5) |  |
|  | 1 | 64 (18.6) | 162 (14.4) |  | 47 (18.9) | 161 (14.3) |  |
|  | 2 | 42 (12.2) | 126 (11.2) |  | 28 (11.2) | 126 (11.2) |  |
| STROKE_T7, n (%) | |  |  | 0.074 |  |  | 0.007 |
|  | 2 | 43 (12.5) | 169 (15.0) |  | 38 (15.3) | 169 (15.0) |  |
| KIDNEY_DISEASE_T7, n (%) | |  |  | 0.144 |  |  | 0.156 |
|  | 0 | 281 (81.4) | 977 (86.7) |  | 202 (81.1) | 977 (86.8) |  |
|  | 1 | 39 (11.3) | 91 (8.1) |  | 27 (10.8) | 90 (8.0) |  |
|  | 2 | 25 (7.2) | 59 (5.2) |  | 20 (8.0) | 59 (5.2) |  |
| CANCER_T7, n (%) | |  |  | 0.025 |  |  | 0.101 |
|  | 0 | 278 (80.6) | 919 (81.5) |  | 193 (77.5) | 918 (81.5) |  |
|  | 1 | 26 (7.5) | 81 (7.2) |  | 21 (8.4) | 81 (7.2) |  |
|  | 2 | 41 (11.9) | 127 (11.3) |  | 35 (14.1) | 127 (11.3) |  |
| DIABETES_impu_new, n (%) | |  |  | 0.043 |  |  | 0.084 |
|  | 0 | 275 (79.7) | 891 (79.1) |  | 195 (78.3) | 890 (79.0) |  |
|  | 1 | 57 (16.5) | 200 (17.7) |  | 42 (16.9) | 200 (17.8) |  |
|  | 2 | 13 (3.8) | 36 (3.2) |  | 12 (4.8) | 36 (3.2) |  |
| Cons_GP_Times_impu, mean (SD) | | 4.28 (5.56) | 4.31 (5.47) | 0.005 | 4.27 (5.41) | 4.29 (5.43) | 0.004 |
| Cons_Emergency_Times_impu, mean (SD) | | 0.43 (0.87) | 0.30 (0.73) | 0.165 | 0.34 (0.74) | 0.30 (0.73) | 0.061 |
| Cons_Hospital_Times_impu, mean (SD) | | 0.43 (1.27) | 0.44 (0.92) | 0.003 | 0.42 (1.14) | 0.43 (0.91) | 0.015 |
| Cons_Specialist_Times_impu, mean (SD) | | 0.51 (1.43) | 0.44 (2.45) | 0.033 | 0.47 (1.34) | 0.44 (2.45) | 0.012 |
| Cons_Clinic_Times_impu, mean (SD) | | 1.32 (4.93) | 1.19 (4.81) | 0.027 | 1.50 (5.66) | 1.19 (4.82) | 0.059 |
| Alcohol_frequency_impu, n (%) | |  |  | 0.150 |  |  | 0.145 |
|  | 1 | 34 (9.9) | 121 (10.7) |  | 27 (10.8) | 121 (10.7) |  |
|  | 2 | 85 (24.6) | 209 (18.5) |  | 58 (23.3) | 208 (18.5) |  |
|  | 3 | 129 (37.4) | 462 (41.0) |  | 100 (40.2) | 462 (41.0) |  |
|  | 4 | 69 (20.0) | 243 (21.6) |  | 43 (17.3) | 243 (21.6) |  |
|  | 5 | 28 (8.1) | 92 (8.2) |  | 21 (8.4) | 92 (8.2) |  |
| Alcohol_units_impu, n (%) | |  |  | 0.075 |  |  | 0.112 |
|  | 0 | 36 (10.4) | 119 (10.6) |  | 27 (10.8) | 119 (10.6) |  |
|  | 1 | 175 (50.7) | 542 (48.1) |  | 127 (51.0) | 541 (48.0) |  |
|  | 2 | 101 (29.3) | 367 (32.6) |  | 69 (27.7) | 367 (32.6) |  |
|  | 3 | 33 (9.6) | 99 (8.8) |  | 26 (10.4) | 99 (8.8) |  |
| Alcohol_units_6_impu, n (%) | |  |  | 0.091 |  |  | 0.115 |
|  | 1 | 189 (54.8) | 664 (58.9) |  | 140 (56.2) | 664 (59.0) |  |
|  | 2 | 120 (34.8) | 364 (32.3) |  | 83 (33.3) | 363 (32.2) |  |
|  | 3 | 27 (7.8) | 74 (6.6) |  | 20 (8.0) | 74 (6.6) |  |
|  | 4 | 7 (2.0) | 21 (1.9) |  | 6 (2.4) | 21 (1.9) |  |
|  | 5 | 2 (0.6) | 4 (0.4) |  | 0 (0.0) | 4 (0.4) |  |
| Smoke_impu, n (%) | |  |  | 0.305 |  |  | 0.197 |
|  | 1 | 57 (16.5) | 139 (12.3) |  | 39 (15.7) | 139 (12.3) |  |
|  | 2 | 12 (3.5) | 19 (1.7) |  | 5 (2.0) | 19 (1.7) |  |
|  | 3 | 167 (48.4) | 711 (63.1) |  | 133 (53.4) | 710 (63.1) |  |
|  | 4 | 109 (31.6) | 258 (22.9) |  | 72 (28.9) | 258 (22.9) |  |
| Snuff_chewing_tobacco_impu, n (%) | |  |  | 0.053 |  |  | 0.080 |
|  | 1 | 22 (6.4) | 68 (6.0) |  | 15 (6.0) | 68 (6.0) |  |
|  | 2 | 1 (0.3) | 1 (0.1) |  | 1 (0.4) | 1 (0.1) |  |
|  | 3 | 19 (5.5) | 57 (5.1) |  | 10 (4.0) | 57 (5.1) |  |
|  | 4 | 303 (87.8) | 1001 (88.8) |  | 223 (89.6) | 1000 (88.8) |  |
| Cod_liver_oil_omega3_impu, n (%) | |  |  | 0.075 |  |  | 0.054 |
|  | 0 | 260 (75.4) | 823 (73.0) |  | 187 (75.1) | 822 (73.0) |  |
|  | 1 | 43 (12.5) | 145 (12.9) |  | 31 (12.4) | 145 (12.9) |  |
|  | 2 | 14 (4.1) | 44 (3.9) |  | 9 (3.6) | 44 (3.9) |  |
|  | 3 | 28 (8.1) | 115 (10.2) |  | 22 (8.8) | 115 (10.2) |  |
| FRUIT_UNITS_T7, mean (SD) | | 1.74 (1.35) | 1.98 (1.76 | 0.152 | 1.76 (1.41) | 1.95 (1.35) | 0.135 |
| RED_MEAT_T7, n (%) | |  |  | 0.055 |  |  | 0.032 |
|  | 1 | 23 (6.7) | 73 (6.5) |  | 17 (6.8) | 73 (6.5) |  |
|  | 2 | 80 (23.2) | 255 (22.6) |  | 55 (22.1) | 255 (22.6) |  |
|  | 3 | 214 (62.0) | 720 (63.9) |  | 158 (63.5) | 719 (63.9) |  |
|  | 4 | 24 (7.0) | 65 (5.8) |  | 16 (6.4) | 65 (5.8) |  |
|  | 5 | 4 (1.2) | 14 (1.2) |  | 3 (1.2) | 14 (1.2) |  |
| FRUITS_VEG_BERRY_T7, n (%) | |  |  | 0.101 |  |  | 0.121 |
|  | 1 | 4 (1.2) | 13 (1.2) |  | 2 (0.8) | 13 (1.2) |  |
|  | 2 | 15 (4.3) | 47 (4.2) |  | 13 (5.2) | 47 (4.2) |  |
|  | 3 | 64 (18.6) | 255 (22.6) |  | 46 (18.5) | 255 (22.6) |  |
|  | 4 | 101 (29.3) | 313 (27.8) |  | 69 (27.7) | 312 (27.7) |  |
|  | 5 | 161 (46.7) | 499 (44.3) |  | 119 (47.8) | 499 (44.3) |  |
| LEAN_FISH_T7, n (%) | |  |  | 0.241 |  |  | 0.102 |
|  | 1 | 24 (7.0) | 28 (2.5) |  | 10 (4.0) | 28 (2.5) |  |
|  | 2 | 53 (15.4) | 153 (13.6) |  | 35 (14.1) | 153 (13.6) |  |
|  | 3 | 223 (64.6) | 800 (71.0) |  | 171 (68.7) | 799 (71.0) |  |
|  | 4 | 38 (11.0) | 134 (11.9) |  | 29 (11.6) | 134 (11.9) |  |
|  | 5 | 7 (2.0) | 12 (1.1) |  | 4 (1.6) | 12 (1.1) |  |
| FAT_FISH_T7, n (%) | |  |  | 0.138 |  |  | 0.110 |
|  | 1 | 50 (14.5) | 115 (10.2) |  | 27 (10.8) | 114 (10.1) |  |
|  | 2 | 116 (33.6) | 389 (34.5) |  | 82 (32.9) | 389 (34.5) |  |
|  | 3 | 157 (45.5) | 550 (48.8) |  | 126 (50.6) | 550 (48.8) |  |
|  | 4 | 15 (4.3) | 55 (4.9) |  | 8 (3.2) | 55 (4.9) |  |
|  | 5 | 7 (2.0) | 18 (1.6) |  | 6 (2.4) | 18 (1.6) |  |
| PHYS_ACTIVITY_LEISURE_T7, n (%) | |  |  | 0.077 |  |  | 0.077 |
|  | 1 | 81 (23.5) | 237 (21.0) |  | 58 (23.3) | 236 (21.0) |  |
|  | 2 | 176 (51.0) | 615 (54.6) |  | 127 (51.0) | 615 (54.6) |  |
|  | 3 | 77 (22.3) | 244 (21.7) |  | 56 (22.5) | 244 (21.7) |  |
|  | 4 | 11 (3.2) | 31 (2.8) |  | 8 (3.2) | 31 (2.8) |  |
| EXERCISE_T7, n (%) | |  |  | 0.169 |  |  | 0.180 |
|  | 1 | 42 (12.2) | 103 (9.1) |  | 33 (13.3) | 103 (9.1) |  |
|  | 2 | 53 (15.4) | 143 (12.7) |  | 35 (14.1) | 143 (12.7) |  |
|  | 3 | 54 (15.7) | 153 (13.6) |  | 35 (14.1) | 153 (13.6) |  |
|  | 4 | 112 (32.5) | 435 (38.6) |  | 78 (31.3) | 434 (38.5) |  |
|  | 5 | 84 (24.3) | 293 (26.0) |  | 68 (27.3) | 293 (26.0) |  |
| **Dataset 6** | | | | | | | |
|  | | **Before matching** | | | **After matching** | | |
|  | | Not LLD user | LLD user | SMD | Not LLD user | LLD user | SMD |
| n | | 345 | 1127 |  | 248 | 1127 |  |
| AGE, mean (SD) | | 66.34 (13.45) | 69.41 (9.72) | 0.261 | 67.70 (12.36) | 69.41 (9.72) | 0.154 |
| SEX_T7, n (%) | |  |  | 0.390 |  |  | 0.236 |
|  | 1 | 193 (55.9) | 836 (74.2) |  | 157 (63.3) | 836 (74.2) |  |
| BMI, mean (SD) | | 28.22 (4.91) | 28.41 (4.36) | 0.042 | 28.51 (4.93) | 28.41 (4.36) | 0.021 |
| HEART_FAILURE_T7, n (%) | |  |  | 0.190 |  |  | 0.160 |
|  | 0 | 282 (81.7) | 841 (74.6) |  | 201 (81.0) | 841 (74.6) |  |
|  | 1 | 31 (9.0) | 116 (10.3) |  | 21 (8.5) | 116 (10.3) |  |
|  | 2 | 32 (9.3) | 170 (15.1) |  | 26 (10.5) | 170 (15.1) |  |
| ATRIAL_FIBRILLATION_T7, n (%) | |  |  | 0.133 |  |  | 0.073 |
|  | 0 | 240 (69.6) | 840 (74.5) |  | 177 (71.4) | 840 (74.5) |  |
|  | 1 | 66 (19.1) | 161 (14.3) |  | 41 (16.5) | 161 (14.3) |  |
|  | 2 | 39 (11.3) | 126 (11.2) |  | 30 (12.1) | 126 (11.2) |  |
| STROKE_T7, n (%) | |  |  | 0.052 |  |  | 0.036 |
|  | 2 | 44 (12.8) | 164 (14.6) |  | 33 (13.3) | 164 (14.6) |  |
| KIDNEY_DISEASE_T7, n (%) | |  |  | 0.145 |  |  | 0.086 |
|  | 0 | 283 (82.0) | 978 (86.8) |  | 208 (83.9) | 978 (86.8) |  |
|  | 1 | 36 (10.4) | 98 (8.7) |  | 25 (10.1) | 98 (8.7) |  |
|  | 2 | 26 (7.5) | 51 (4.5) |  | 15 (6.0) | 51 (4.5) |  |
| CANCER_T7, n (%) | |  |  | 0.019 |  |  | 0.076 |
|  | 0 | 280 (81.2) | 922 (81.8) |  | 198 (79.8) | 922 (81.8) |  |
|  | 1 | 27 (7.8) | 87 (7.7) |  | 18 (7.3) | 87 (7.7) |  |
|  | 2 | 38 (11.0) | 118 (10.5) |  | 32 (12.9) | 118 (10.5) |  |
| DIABETES_impu_new, n (%) | |  |  | 0.104 |  |  | 0.114 |
|  | 0 | 273 (79.1) | 887 (78.7) |  | 196 (79.0) | 887 (78.7) |  |
|  | 1 | 52 (15.1) | 196 (17.4) |  | 37 (14.9) | 196 (17.4) |  |
|  | 2 | 20 (5.8) | 44 (3.9) |  | 15 (6.0) | 44 (3.9) |  |
| Cons_GP_Times_impu, mean (SD) | | 4.19 (5.30) | 4.14 (4.62) | 0.009 | 4.28 (5.72) | 4.14 (4.62) | 0.027 |
| Cons_Emergency_Times_impu, mean (SD) | | 0.39 (0.81) | 0.30 (0.72) | 0.129 | 0.34 (0.73) | 0.30 (0.72) | 0.066 |
| Cons_Hospital_Times_impu, mean (SD) | | 0.43 (1.26) | 0.43 (0.91) | 0.002 | 0.47 (1.41) | 0.43 (0.91) | 0.033 |
| Cons_Specialist_Times_impu, mean (SD) | | 0.60 (1.69) | 0.52 (2.67) | 0.033 | 0.55 (1.47) | 0.52 (2.67) | 0.013 |
| Cons_Clinic_Times_impu, mean (SD) | | 1.29 (4.79) | 1.28 (5.18) | 0.001 | 1.25 (5.35) | 1.28 (5.18) | 0.006 |
| Alcohol_frequency_impu, n (%) | |  |  | 0.150 |  |  | 0.159 |
|  | 1 | 34 (9.9) | 121 (10.7) |  | 25 (10.1) | 121 (10.7) |  |
|  | 2 | 85 (24.6) | 209 (18.5) |  | 62 (25.0) | 209 (18.5) |  |
|  | 3 | 129 (37.4) | 462 (41.0) |  | 92 (37.1) | 462 (41.0) |  |
|  | 4 | 69 (20.0) | 243 (21.6) |  | 49 (19.8) | 243 (21.6) |  |
|  | 5 | 28 (8.1) | 92 (8.2) |  | 20 (8.1) | 92 (8.2) |  |
| Alcohol_units_impu, n (%) | |  |  | 0.074 |  |  | 0.038 |
|  | 0 | 36 (10.4) | 119 (10.6) |  | 25 (10.1) | 119 (10.6) |  |
|  | 1 | 175 (50.7) | 541 (48.0) |  | 121 (48.8) | 541 (48.0) |  |
|  | 2 | 101 (29.3) | 367 (32.6) |  | 78 (31.5) | 367 (32.6) |  |
|  | 3 | 33 (9.6) | 100 (8.9) |  | 24 (9.7) | 100 (8.9) |  |
| Alcohol_units_6_impu, n (%) | |  |  | 0.078 |  |  | 0.100 |
|  | 1 | 191 (55.4) | 663 (58.8) |  | 139 (56.0) | 663 (58.8) |  |
|  | 2 | 118 (34.2) | 363 (32.2) |  | 82 (33.1) | 363 (32.2) |  |
|  | 3 | 26 (7.5) | 75 (6.7) |  | 21 (8.5) | 75 (6.7) |  |
|  | 4 | 8 (2.3) | 22 (2.0) |  | 4 (1.6) | 22 (2.0) |  |
|  | 5 | 2 (0.6) | 4 (0.4) |  | 2 (0.8) | 4 (0.4) |  |
| Smoke_impu, n (%) | |  |  | 0.305 |  |  | 0.198 |
|  | 1 | 57 (16.5) | 139 (12.3) |  | 39 (15.7) | 139 (12.3) |  |
|  | 2 | 12 (3.5) | 19 (1.7) |  | 7 (2.8) | 19 (1.7) |  |
|  | 3 | 167 (48.4) | 711 (63.1) |  | 133 (53.6) | 711 (63.1) |  |
|  | 4 | 109 (31.6) | 258 (22.9) |  | 69 (27.8) | 258 (22.9) |  |
| Snuff_chewing_tobacco_impu, n (%) | |  |  | 0.053 |  |  | 0.067 |
|  | 1 | 22 (6.4) | 67 (5.9) |  | 16 (6.5) | 67 (5.9) |  |
|  | 2 | 1 (0.3) | 1 (0.1) |  | 1 (0.4) | 1 (0.1) |  |
|  | 3 | 19 (5.5) | 58 (5.1) |  | 13 (5.2) | 58 (5.1) |  |
|  | 4 | 303 (87.8) | 1001 (88.8) |  | 218 (87.9) | 1001 (88.8) |  |
| Cod_liver_oil_omega3_impu, n (%) | |  |  | 0.085 |  |  | 0.118 |
|  | 0 | 261 (75.7) | 820 (72.8) |  | 188 (75.8) | 820 (72.8) |  |
|  | 1 | 43 (12.5) | 146 (13.0) |  | 33 (13.3) | 146 (13.0) |  |
|  | 2 | 14 (4.1) | 47 (4.2) |  | 10 (4.0) | 47 (4.2) |  |
|  | 3 | 27 (7.8) | 114 (10.1) |  | 17 (6.9) | 114 (10.1) |  |
| FRUIT_UNITS_T7, mean (SD) | | 1.70 (1.34) | 1.96 (1.71) | 0.169 | 1.81 (1.44) | 1.96 (1.71) | 0.099 |
| RED_MEAT_T7, n (%) | |  |  | 0.089 |  |  | 0.115 |
|  | 1 | 23 (6.7) | 70 (6.2) |  | 11 (4.4) | 70 (6.2) |  |
|  | 2 | 76 (22.0) | 255 (22.6) |  | 57 (23.0) | 255 (22.6) |  |
|  | 3 | 214 (62.0) | 723 (64.2) |  | 158 (63.7) | 723 (64.2) |  |
|  | 4 | 28 (8.1) | 67 (5.9) |  | 20 (8.1) | 67 (5.9) |  |
|  | 5 | 4 (1.2) | 12 (1.1) |  | 2 (0.8) | 12 (1.1) |  |
| FRUITS_VEG_BERRY_T7, n (%) | |  |  | 0.108 |  |  | 0.083 |
|  | 1 | 3 (0.9) | 13 (1.2) |  | 2 (0.8) | 13 (1.2) |  |
|  | 2 | 17 (4.9) | 46 (4.1) |  | 10 (4.0) | 46 (4.1) |  |
|  | 3 | 65 (18.8) | 257 (22.8) |  | 50 (20.2) | 257 (22.8) |  |
|  | 4 | 99 (28.7) | 312 (27.7) |  | 75 (30.2) | 312 (27.7) |  |
|  | 5 | 161 (46.7) | 499 (44.3) |  | 111 (44.8) | 499 (44.3) |  |
| LEAN_FISH_T7, n (%) | |  |  | 0.261 |  |  | 0.169 |
|  | 1 | 28 (8.1) | 28 (2.5) |  | 13 (5.2) | 28 (2.5) |  |
|  | 2 | 51 (14.8) | 152 (13.5) |  | 36 (14.5) | 152 (13.5) |  |
|  | 3 | 224 (64.9) | 797 (70.7) |  | 173 (69.8) | 797 (70.7) |  |
|  | 4 | 37 (10.7) | 134 (11.9) |  | 24 (9.7) | 134 (11.9) |  |
|  | 5 | 5 (1.4) | 16 (1.4) |  | 2 (0.8) | 16 (1.4) |  |
| FAT_FISH_T7, n (%) | |  |  | 0.180 |  |  | 0.122 |
|  | 1 | 55 (15.9) | 114 (10.1) |  | 34 (13.7) | 114 (10.1) |  |
|  | 2 | 118 (34.2) | 389 (34.5) |  | 82 (33.1) | 389 (34.5) |  |
|  | 3 | 152 (44.1) | 549 (48.7) |  | 118 (47.6) | 549 (48.7) |  |
|  | 4 | 15 (4.3) | 54 (4.8) |  | 11 (4.4) | 54 (4.8) |  |
|  | 5 | 5 (1.4) | 21 (1.9) |  | 3 (1.2) | 21 (1.9) |  |
| PHYS_ACTIVITY_LEISURE_T7, n (%) | |  |  | 0.096 |  |  | 0.142 |
|  | 1 | 84 (24.3) | 235 (20.9) |  | 63 (25.4) | 235 (20.9) |  |
|  | 2 | 176 (51.0) | 621 (55.1) |  | 122 (49.2) | 621 (55.1) |  |
|  | 3 | 75 (21.7) | 243 (21.6) |  | 54 (21.8) | 243 (21.6) |  |
|  | 4 | 10 (2.9) | 28 (2.5) |  | 9 (3.6) | 28 (2.5) |  |
| EXERCISE_T7, n (%) | |  |  | 0.186 |  |  | 0.194 |
|  | 1 | 46 (13.3) | 105 (9.3) |  | 32 (12.9) | 105 (9.3) |  |
|  | 2 | 53 (15.4) | 144 (12.8) |  | 37 (14.9) | 144 (12.8) |  |
|  | 3 | 51 (14.8) | 154 (13.7) |  | 36 (14.5) | 154 (13.7) |  |
|  | 4 | 109 (31.6) | 436 (38.7) |  | 75 (30.2) | 436 (38.7) |  |
|  | 5 | 86 (24.9) | 288 (25.6) |  | 68 (27.4) | 288 (25.6) |  |
| **Dataset 7** | | | | | | | |
|  | | **Before matching** | | | **After matching** | | |
|  | | Not LLD user | LLD user | SMD | Not LLD user | LLD user | SMD |
| n | | 345 | 1127 |  | 250 | 1127 |  |
| AGE, mean (SD) | | 66.34 (13.45) | 69.41 (9.72) | 0.261 | 67.36 (13.22) | 69.41 (9.72) | 0.176 |
| SEX_T7, n (%) | |  |  |  |  |  | 0.238 |
|  | 1 | 193 (55.9) | 836 (74.2) | 0.390 | 158 (63.2) | 836 (74.2) |  |
| BMI, mean (SD) | | 28.28 (4.95) | 28.41 (4.35) | 0.028 | 28.41 (4.65) | 28.41 (4.35) | 0.001 |
| HEART_FAILURE_T7, n (%) | |  |  | 0.182 |  |  | 0.115 |
|  | 0 | 281 (81.4) | 839 (74.4) |  | 198 (79.2) | 839 (74.4) |  |
|  | 1 | 29 (8.4) | 110 (9.8) |  | 21 (8.4) | 110 (9.8) |  |
|  | 2 | 35 (10.1) | 178 (15.8) |  | 31 (12.4) | 178 (15.8) |  |
| ATRIAL_FIBRILLATION_T7, n (%) | |  |  | 0.138 |  |  | 0.128 |
|  | 0 | 238 (69.0) | 844 (74.9) |  | 173 (69.2) | 844 (74.9) |  |
|  | 1 | 66 (19.1) | 164 (14.6) |  | 46 (18.4) | 164 (14.6) |  |
|  | 2 | 41 (11.9) | 119 (10.6) |  | 31 (12.4) | 119 (10.6) |  |
| STROKE_T7, n (%) | |  |  | 0.060 |  |  | 0.041 |
|  | 2 | 42 (12.2) | 160 (14.2) |  | 32 (12.8) | 160 (14.2) |  |
| KIDNEY_DISEASE_T7, n (%) | |  |  | 0.144 |  |  | 0.151 |
|  | 0 | 281 (81.4) | 977 (86.7) |  | 203 (81.2) | 977 (86.7) |  |
|  | 1 | 39 (11.3) | 92 (8.2) |  | 30 (12.0) | 92 (8.2) |  |
|  | 2 | 25 (7.2) | 58 (5.1) |  | 17 (6.8) | 58 (5.1) |  |
| CANCER_T7, n (%) | |  |  | 0.046 |  |  | 0.092 |
|  | 0 | 278 (80.6) | 921 (81.7) |  | 200 (80.0) | 921 (81.7) |  |
|  | 1 | 30 (8.7) | 84 (7.5) |  | 25 (10.0) | 84 (7.5) |  |
|  | 2 | 37 (10.7) | 122 (10.8) |  | 25 (10.0) | 122 (10.8) |  |
| DIABETES_impu_new, n (%) | |  |  | 0.135 |  |  | 0.096 |
|  | 0 | 273 (79.1) | 887 (78.7) |  | 197 (78.8) | 887 (78.7) |  |
|  | 1 | 53 (15.4) | 205 (18.2) |  | 41 (16.4) | 205 (18.2) |  |
|  | 2 | 19 (5.5) | 35 (3.1) |  | 12 (4.8) | 35 (3.1) |  |
| Cons_GP_Times_impu, mean (SD) | | 4.48 (5.93) | 4.11 (4.67) | 0.069 | 4.22 (5.19) | 4.11 (4.67) | 0.024 |
| Cons_Emergency_Times_impu, mean (SD) | | 0.39 (0.81) | 0.31 (0.73) | 0.107 | 0.34 (0.78) | 0.31 (0.73) | 0.047 |
| Cons_Hospital_Times_impu, mean (SD) | | 0.44 (1.28) | 0.43 (0.96) | 0.012 | 0.36 (1.01) | 0.43 (0.96) | 0.076 |
| Cons_Specialist_Times_impu, mean (SD) | | 0.66 (2.14) | 0.52 (2.68) | 0.055 | 0.66 (2.27) | 0.52 (2.68) | 0.057 |
| Cons_Clinic_Times_impu, mean (SD) | | 1.26 (4.77) | 1.17 (4.76) | 0.019 | 1.32 (5.44) | 1.17 (4.76) | 0.029 |
| Alcohol_frequency_impu, n (%) | |  |  | 0.157 |  |  | 0.150 |
|  | 1 | 34 (9.9) | 121 (10.7) |  | 23 (9.2) | 121 (10.7) |  |
|  | 2 | 86 (24.9) | 209 (18.5) |  | 59 (23.6) | 209 (18.5) |  |
|  | 3 | 128 (37.1) | 462 (41.0) |  | 91 (36.4) | 462 (41.0) |  |
|  | 4 | 69 (20.0) | 243 (21.6) |  | 53 (21.2) | 243 (21.6) |  |
|  | 5 | 28 (8.1) | 92 (8.2) |  | 24 (9.6) | 92 (8.2) |  |
| Alcohol_units_impu, n (%) | |  |  | 0.074 |  |  | 0.074 |
|  | 0 | 36 (10.4) | 119 (10.6) |  | 23 (9.2) | 119 (10.6) |  |
|  | 1 | 175 (50.7) | 541 (48.0) |  | 127 (50.8) | 541 (48.0) |  |
|  | 2 | 101 (29.3) | 367 (32.6) |  | 76 (30.4) | 367 (32.6) |  |
|  | 3 | 33 (9.6) | 100 (8.9) |  | 24 (9.6) | 100 (8.9) |  |
| Alcohol_units_6_impu, n (%) | |  |  | 0.090 |  |  | 0.111 |
|  | 1 | 190 (55.1) | 664 (58.9) |  | 139 (55.6) | 664 (58.9) |  |
|  | 2 | 119 (34.5) | 362 (32.1) |  | 83 (33.2) | 362 (32.1) |  |
|  | 3 | 27 (7.8) | 77 (6.8) |  | 19 (7.6) | 77 (6.8) |  |
|  | 4 | 7 (2.0) | 21 (1.9) |  | 7 (2.8) | 21 (1.9) |  |
|  | 5 | 2 (0.6) | 3 (0.3) |  | 2 (0.8) | 3 (0.3) |  |
| Smoke_impu, n (%) | |  |  | 0.305 |  |  | 0.197 |
|  | 1 | 57 (16.5) | 139 (12.3) |  | 37 (14.8) | 139 (12.3) |  |
|  | 2 | 12 (3.5) | 19 (1.7) |  | 9 (3.6) | 19 (1.7) |  |
|  | 3 | 167 (48.4) | 711 (63.1) |  | 136 (54.4) | 711 (63.1) |  |
|  | 4 | 109 (31.6) | 258 (22.9) |  | 68 (27.2) | 258 (22.9) |  |
| Snuff_chewing_tobacco_impu, n (%) | |  |  | 0.054 |  |  | 0.086 |
|  | 1 | 22 (6.4) | 67 (5.9) |  | 18 (7.2) | 67 (5.9) |  |
|  | 2 | 1 (0.3) | 1 (0.1) |  | 1 (0.4) | 1 (0.1) |  |
|  | 3 | 19 (5.5) | 57 (5.1) |  | 14 (5.6) | 57 (5.1) |  |
|  | 4 | 303 (87.8) | 1002 (88.9) |  | 217 (86.8) | 1002 (88.9) |  |
| Cod_liver_oil_omega3_impu, n (%) | |  |  | 0.074 |  |  | 0.039 |
|  | 0 | 259 (75.1) | 822 (72.9) |  | 186 (74.4) | 822 (72.9) |  |
|  | 1 | 43 (12.5) | 143 (12.7) |  | 30 (12.0) | 143 (12.7) |  |
|  | 2 | 15 (4.3) | 47 (4.2) |  | 9 (3.6) | 47 (4.2) |  |
|  | 3 | 28 (8.1) | 115 (10.2) |  | 25 (10.0) | 115 (10.2) |  |
| FRUIT_UNITS_T7, mean (SD) | | 1.72 (1.33) | 1.97 (1.72) | 0.165 | 1.74 (1.38) | 1.97 (1.72) | 0.150 |
| RED_MEAT_T7, n (%) | |  |  | 0.079 |  |  | 0.089 |
|  | 1 | 24 (7.0) | 72 (6.4) |  | 14 (5.6) | 72 (6.4) |  |
|  | 2 | 80 (23.2) | 254 (22.5) |  | 58 (23.2) | 254 (22.5) |  |
|  | 3 | 212 (61.4) | 721 (64.0) |  | 155 (62.0) | 721 (64.0) |  |
|  | 4 | 26 (7.5) | 67 (5.9) |  | 20 (8.0) | 67 (5.9) |  |
|  | 5 | 3 (0.9) | 13 (1.2) |  | 3 (1.2) | 13 (1.2) |  |
| FRUITS_VEG_BERRY_T7, n (%) | |  |  | 0.091 |  |  | 0.029 |
|  | 1 | 5 (1.4) | 14 (1.2) |  | 3 (1.2) | 14 (1.2) |  |
|  | 2 | 16 (4.6) | 43 (3.8) |  | 10 (4.0) | 43 (3.8) |  |
|  | 3 | 66 (19.1) | 254 (22.5) |  | 55 (22.0) | 254 (22.5) |  |
|  | 4 | 99 (28.7) | 316 (28.0) |  | 68 (27.2) | 316 (28.0) |  |
|  | 5 | 159 (46.1) | 500 (44.4) |  | 114 (45.6) | 500 (44.4) |  |
| LEAN_FISH_T7, n (%) | |  |  | 0.225 |  |  | 0.181 |
|  | 1 | 22 (6.4) | 26 (2.3) |  | 11 (4.4) | 26 (2.3) |  |
|  | 2 | 52 (15.1) | 153 (13.6) |  | 43 (17.2) | 153 (13.6) |  |
|  | 3 | 224 (64.9) | 799 (70.9) |  | 161 (64.4) | 799 (70.9) |  |
|  | 4 | 40 (11.6) | 136 (12.1) |  | 30 (12.0) | 136 (12.1) |  |
|  | 5 | 7 (2.0) | 13 (1.2) |  | 5 (2.0) | 13 (1.2) |  |
| FAT_FISH_T7, n (%) | |  |  | 0.163 |  |  | 0.125 |
|  | 1 | 52 (15.1) | 114 (10.1) |  | 33 (13.2) | 114 (10.1) |  |
|  | 2 | 115 (33.3) | 388 (34.4) |  | 85 (34.0) | 388 (34.4) |  |
|  | 3 | 158 (45.8) | 553 (49.1) |  | 120 (48.0) | 553 (49.1) |  |
|  | 4 | 13 (3.8) | 55 (4.9) |  | 8 (3.2) | 55 (4.9) |  |
|  | 5 | 7 (2.0) | 17 (1.5) |  | 4 (1.6) | 17 (1.5) |  |
| PHYS_ACTIVITY_LEISURE_T7, n (%) | |  |  | 0.085 |  |  | 0.105 |
|  | 1 | 84 (24.3) | 242 (21.5) |  | 60 (24.0) | 242 (21.5) |  |
|  | 2 | 175 (50.7) | 610 (54.1) |  | 123 (49.2) | 610 (54.1) |  |
|  | 3 | 76 (22.0) | 249 (22.1) |  | 62 (24.8) | 249 (22.1) |  |
|  | 4 | 10 (2.9) | 26 (2.3) |  | 5 (2.0) | 26 (2.3) |  |
| EXERCISE_T7, n (%) | |  |  | 0.182 |  |  | 0.121 |
|  | 1 | 45 (13.0) | 101 (9.0) |  | 25 (10.0) | 101 (9.0) |  |
|  | 2 | 54 (15.7) | 146 (13.0) |  | 40 (16.0) | 146 (13.0) |  |
|  | 3 | 51 (14.8) | 155 (13.8) |  | 38 (15.2) | 155 (13.8) |  |
|  | 4 | 112 (32.5) | 436 (38.7) |  | 86 (34.4) | 436 (38.7) |  |
|  | 5 | 83 (24.1) | 289 (25.6) |  | 61 (24.4) | 289 (25.6) |  |
| **Dataset 8** | | | | | | | |
|  | | **Before matching** | | | **After matching** | | |
|  | | Not LLD user | LLD user | SMD | Not LLD user | LLD user | SMD |
| n | | 345 | 1127 |  | 251 | 1127 |  |
| AGE, mean (SD) | | 66.34 (13.45) | 69.41 (9.72) | 0.261 | 67.80 (12.91) | 69.41 (9.72) | 0.141 |
| SEX_T7, n (%) | |  |  | 0.390 |  |  | 0.252 |
|  | 1 | 193 (55.9) | 836 (74.2) |  | 157 (62.5) | 836 (74.2) |  |
| BMI, mean (SD) | | 28.28 (4.95) | 28.40 (4.35) | 0.027 | 28.51 (5.00) | 28.40 (4.35) | 0.024 |
| HEART_FAILURE_T7, n (%) | |  |  | 0.191 |  |  | 0.118 |
|  | 0 | 281 (81.4) | 838 (74.4) |  | 197 (78.5) | 838 (74.4) |  |
|  | 1 | 31 (9.0) | 114 (10.1) |  | 25 (10.0) | 114 (10.1) |  |
|  | 2 | 33 (9.6) | 175 (15.5) |  | 29 (11.6) | 175 (15.5) |  |
| ATRIAL_FIBRILLATION_T7, n (%) | |  |  | 0.155 |  |  | 0.114 |
|  | 0 | 233 (67.5) | 837 (74.3) |  | 176 (70.1) | 837 (74.3) |  |
|  | 1 | 68 (19.7) | 164 (14.6) |  | 47 (18.7) | 164 (14.6) |  |
|  | 2 | 44 (12.8) | 126 (11.2) |  | 28 (11.2) | 126 (11.2) |  |
| STROKE_T7, n (%) | |  |  | 0.045 |  |  | 0.024 |
|  | 2 | 44 (12.8) | 161 (14.3) |  | 38 (15.1) | 161 (14.3) |  |
| KIDNEY_DISEASE_T7, n (%) | |  |  | 0.129 |  |  | 0.127 |
|  | 0 | 283 (82.0) | 976 (86.6) |  | 206 (82.1) | 976 (86.6) |  |
|  | 1 | 36 (10.4) | 93 (8.3) |  | 29 (11.6) | 93 (8.3) |  |
|  | 2 | 26 (7.5) | 58 (5.1) |  | 16 (6.4) | 58 (5.1) |  |
| CANCER_T7, n (%) | |  |  | 0.072 |  |  | 0.079 |
|  | 0 | 278 (80.6) | 921 (81.7) |  | 200 (79.7) | 921 (81.7) |  |
|  | 1 | 32 (9.3) | 83 (7.4) |  | 24 (9.6) | 83 (7.4) |  |
|  | 2 | 35 (10.1) | 123 (10.9) |  | 27 (10.8) | 123 (10.9) |  |
| DIABETES_impu_new, n (%) | |  |  | 0.056 |  |  | 0.048 |
|  | 0 | 275 (79.7) | 889 (78.9) |  | 197 (78.5) | 889 (78.9) |  |
|  | 1 | 58 (16.8) | 207 (18.4) |  | 45 (17.9) | 207 (18.4) |  |
|  | 2 | 12 (3.5) | 31 (2.8) |  | 9 (3.6) | 31 (2.8) |  |
| Cons_GP_Times_impu, mean (SD) | | 4.34 (6.24) | 4.12 (4.59) | 0.040 | 4.48 (6.87) | 4.12 (4.59) | 0.061 |
| Cons_Emergency_Times_impu, mean (SD) | | 0.41 (0.84) | 0.30 (0.72) | 0.148 | 0.41 (0.79) | 0.30 (0.72) | 0.152 |
| Cons_Hospital_Times_impu, mean (SD) | | 0.47 (1.29) | 0.43 (0.90) | 0.033 | 0.52 (1.42) | 0.43 (0.90) | 0.077 |
| Cons_Specialist_Times_impu, mean (SD) | | 0.63 (1.82) | 0.48 (2.57) | 0.067 | 0.65 (1.91) | 0.48 (2.57) | 0.072 |
| Cons_Clinic_Times_impu, mean (SD) | | 1.14 (2.55) | 1.19 (4.79) | 0.013 | 1.23 (2.70) | 1.19 (4.79) | 0.008 |
| Alcohol_frequency_impu, n (%) | |  |  | 0.150 |  |  | 0.108 |
|  | 1 | 34 (9.9) | 121 (10.7) |  | 26 (10.4) | 121 (10.7) |  |
|  | 2 | 85 (24.6) | 209 (18.5) |  | 57 (22.7) | 209 (18.5) |  |
|  | 3 | 129 (37.4) | 462 (41.0) |  | 98 (39.0) | 462 (41.0) |  |
|  | 4 | 69 (20.0) | 243 (21.6) |  | 49 (19.5) | 243 (21.6) |  |
|  | 5 | 28 (8.1) | 92 (8.2) |  | 21 (8.4) | 92 (8.2) |  |
| Alcohol_units_impu, n (%) | |  |  | 0.075 |  |  | 0.095 |
|  | 0 | 36 (10.4) | 119 (10.6) |  | 25 (10.0) | 119 (10.6) |  |
|  | 1 | 176 (51.0) | 540 (47.9) |  | 132 (52.6) | 540 (47.9) |  |
|  | 2 | 101 (29.3) | 367 (32.6) |  | 74 (29.5) | 367 (32.6) |  |
|  | 3 | 32 (9.3) | 101 (9.0) |  | 20 (8.0) | 101 (9.0) |  |
| Alcohol_units_6_impu, n (%) | |  |  | 0.104 |  |  | 0.083 |
|  | 1 | 190 (55.1) | 663 (58.8) |  | 142 (56.6) | 663 (58.8) |  |
|  | 2 | 119 (34.5) | 364 (32.3) |  | 84 (33.5) | 364 (32.3) |  |
|  | 3 | 27 (7.8) | 74 (6.6) |  | 19 (7.6) | 74 (6.6) |  |
|  | 4 | 6 (1.7) | 22 (2.0) |  | 4 (1.6) | 22 (2.0) |  |
|  | 5 | 3 (0.9) | 4 (0.4) |  | 2 (0.8) | 4 (0.4) |  |
| Smoke_impu, n (%) | |  |  | 0.305 |  |  | 0.170 |
|  | 1 | 57 (16.5) | 139 (12.3) |  | 41 (16.3) | 139 (12.3) |  |
|  | 2 | 12 (3.5) | 19 (1.7) |  | 5 (2.0) | 19 (1.7) |  |
|  | 3 | 167 (48.4) | 711 (63.1) |  | 138 (55.0) | 711 (63.1) |  |
|  | 4 | 109 (31.6) | 258 (22.9) |  | 67 (26.7) | 258 (22.9) |  |
| Snuff_chewing_tobacco_impu, n (%) | |  |  | 0.054 |  |  | 0.073 |
|  | 1 | 22 (6.4) | 67 (5.9) |  | 17 (6.8) | 67 (5.9) |  |
|  | 2 | 1 (0.3) | 1 (0.1) |  | 1 (0.4) | 1 (0.1) |  |
|  | 3 | 19 (5.5) | 57 (5.1) |  | 12 (4.8) | 57 (5.1) |  |
|  | 4 | 303 (87.8) | 1002 (88.9) |  | 221 (88.0) | 1002 (88.9) |  |
| Cod_liver_oil_omega3_impu, n (%) | |  |  | 0.101 |  |  | 0.087 |
|  | 0 | 261 (75.7) | 821 (72.8) |  | 190 (75.7) | 821 (72.8) |  |
|  | 1 | 44 (12.8) | 143 (12.7) |  | 32 (12.7) | 143 (12.7) |  |
|  | 2 | 14 (4.1) | 46 (4.1) |  | 8 (3.2) | 46 (4.1) |  |
|  | 3 | 26 (7.5) | 117 (10.4) |  | 21 (8.4) | 117 (10.4) |  |
| FRUIT_UNITS_T7, mean (SD) | | 1.71 (1.33) | 1.97 (1.72) | 0.168 | 1.69 (1.35) | 1.97 (1.72) | 0.181 |
| RED_MEAT_T7, n (%) | |  |  | 0.043 |  |  | 0.046 |
|  | 1 | 23 (6.7) | 70 (6.2) |  | 16 (6.4) | 70 (6.2) |  |
|  | 2 | 79 (22.9) | 255 (22.6) |  | 58 (23.1) | 255 (22.6) |  |
|  | 3 | 215 (62.3) | 719 (63.8) |  | 156 (62.2) | 719 (63.8) |  |
|  | 4 | 24 (7.0) | 69 (6.1) |  | 17 (6.8) | 69 (6.1) |  |
|  | 5 | 4 (1.2) | 14 (1.2) |  | 4 (1.6) | 14 (1.2) |  |
| FRUITS_VEG_BERRY_T7, n (%) | |  |  | 0.107 |  |  | 0.069 |
|  | 1 | 4 (1.2) | 13 (1.2) |  | 3 (1.2) | 13 (1.2) |  |
|  | 2 | 17 (4.9) | 44 (3.9) |  | 12 (4.8) | 44 (3.9) |  |
|  | 3 | 65 (18.8) | 257 (22.8) |  | 54 (21.5) | 257 (22.8) |  |
|  | 4 | 102 (29.6) | 313 (27.8) |  | 75 (29.9) | 313 (27.8) |  |
|  | 5 | 157 (45.5) | 500 (44.4) |  | 107 (42.6) | 500 (44.4) |  |
| LEAN_FISH_T7, n (%) | |  |  | 0.225 |  |  | 0.074 |
|  | 1 | 23 (6.7) | 26 (2.3) |  | 8 (3.2) | 26 (2.3) |  |
|  | 2 | 53 (15.4) | 152 (13.5) |  | 37 (14.7) | 152 (13.5) |  |
|  | 3 | 227 (65.8) | 803 (71.3) |  | 176 (70.1) | 803 (71.3) |  |
|  | 4 | 38 (11.0) | 134 (11.9) |  | 27 (10.8) | 134 (11.9) |  |
|  | 5 | 4 (1.2) | 12 (1.1) |  | 3 (1.2) | 12 (1.1) |  |
| FAT_FISH_T7, n (%) | |  |  | 0.175 |  |  | 0.125 |
|  | 1 | 54 (15.7) | 115 (10.2) |  | 31 (12.4) | 115 (10.2) |  |
|  | 2 | 116 (33.6) | 387 (34.3) |  | 85 (33.9) | 387 (34.3) |  |
|  | 3 | 155 (44.9) | 552 (49.0) |  | 124 (49.4) | 552 (49.0) |  |
|  | 4 | 13 (3.8) | 55 (4.9) |  | 7 (2.8) | 55 (4.9) |  |
|  | 5 | 7 (2.0) | 18 (1.6) |  | 4 (1.6) | 18 (1.6) |  |
| PHYS_ACTIVITY_LEISURE_T7, n (%) | |  |  | 0.080 |  |  | 0.061 |
|  | 1 | 79 (22.9) | 241 (21.4) |  | 56 (22.3) | 241 (21.4) |  |
|  | 2 | 179 (51.9) | 617 (54.7) |  | 130 (51.8) | 617 (54.7) |  |
|  | 3 | 77 (22.3) | 247 (21.9) |  | 60 (23.9) | 247 (21.9) |  |
|  | 4 | 10 (2.9) | 22 (2.0) |  | 5 (2.0) | 22 (2.0) |  |
| EXERCISE_T7, n (%) | |  |  | 0.169 |  |  | 0.168 |
|  | 1 | 43 (12.5) | 106 (9.4) |  | 32 (12.7) | 106 (9.4) |  |
|  | 2 | 55 (15.9) | 142 (12.6) |  | 38 (15.1) | 142 (12.6) |  |
|  | 3 | 51 (14.8) | 157 (13.9) |  | 40 (15.9) | 157 (13.9) |  |
|  | 4 | 111 (32.2) | 433 (38.4) |  | 86 (34.3) | 433 (38.4) |  |
|  | 5 | 85 (24.6) | 289 (25.6) |  | 55 (21.9) | 289 (25.6) |  |
| **Dataset 9** | | | | | | | |
|  | | **Before matching** | | | **After matching** | | |
|  | | Not LLD user | LLD user | SMD | Not LLD user | LLD user | SMD |
| n | | 345 | 1127 |  | 259 | 1126 |  |
| AGE, mean (SD) | | 66.34 (13.45) | 69.41 (9.72) | 0.261 | 67.54 (12.90) | 69.40 (9.72) | 0.163 |
| SEX_T7, n (%) | |  |  | 0.390 |  |  | 0.237 |
|  | 1 | 193 (55.9) | 836 (74.2) |  | 164 (63.3) | 836 (74.2) |  |
| BMI, mean (SD) | | 28.25 (4.96) | 28.41 (4.36) | 0.034 | 28.66 (4.84) | 28.41 (4.36) | 0.054 |
| HEART_FAILURE_T7, n (%) | |  |  | 0.172 |  |  | 0.155 |
|  | 0 | 279 (80.9) | 841 (74.6) |  | 206 (79.5) | 840 (74.6) |  |
|  | 1 | 30 (8.7) | 105 (9.3) |  | 25 (9.7) | 105 (9.3) |  |
|  | 2 | 36 (10.4) | 181 (16.1) |  | 28 (10.8) | 181 (16.1) |  |
| ATRIAL_FIBRILLATION_T7, n (%) | |  |  | 0.133 |  |  | 0.100 |
|  | 0 | 237 (68.7) | 841 (74.6) |  | 182 (70.3) | 841 (74.7) |  |
|  | 1 | 62 (18.0) | 159 (14.1) |  | 42 (16.2) | 158 (14.0) |  |
|  | 2 | 46 (13.3) | 127 (11.3) |  | 35 (13.5) | 127 (11.3) |  |
| STROKE_T7, n (%) | |  |  | 0.054 |  |  | 0.046 |
|  | 2 | 43 (12.5) | 161 (14.3) |  | 33 (12.7) | 161 (14.3) |  |
| KIDNEY_DISEASE_T7, n (%) | |  |  | 0.141 |  |  | 0.131 |
|  | 0 | 281 (81.4) | 975 (86.5) |  | 212 (81.9) | 975 (86.6) |  |
|  | 1 | 39 (11.3) | 88 (7.8) |  | 28 (10.8) | 87 (7.7) |  |
|  | 2 | 25 (7.2) | 64 (5.7) |  | 19 (7.3) | 64 (5.7) |  |
| CANCER_T7, n (%) | |  |  | 0.024 |  |  | 0.038 |
|  | 0 | 279 (80.9) | 921 (81.7) |  | 208 (80.3) | 920 (81.7) |  |
|  | 1 | 27 (7.8) | 82 (7.3) |  | 21 (8.1) | 82 (7.3) |  |
|  | 2 | 39 (11.3) | 124 (11.0) |  | 30 (11.6) | 124 (11.0) |  |
| DIABETES_impu_new, n (%) | |  |  | 0.124 |  |  | 0.100 |
|  | 0 | 275 (79.7) | 886 (78.6) |  | 205 (79.2) | 885 (78.6) |  |
|  | 1 | 49 (14.2) | 196 (17.4) |  | 39 (15.1) | 196 (17.4) |  |
|  | 2 | 21 (6.1) | 45 (4.0) |  | 15 (5.8) | 45 (4.0) |  |
| Cons_GP_Times_impu, mean (SD) | | 4.24 (5.14) | 4.09 (4.47) | 0.031 | 4.11 (5.06) | 4.09 (4.47) | 0.005 |
| Cons_Emergency_Times_impu, mean (SD) | | 0.41 (0.85) | 0.32 (0.77) | 0.114 | 0.36 (0.77) | 0.32 (0.77) | 0.052 |
| Cons_Hospital_Times_impu, mean (SD) | | 0.47 (1.41) | 0.43 (0.90) | 0.039 | 0.42 (1.27) | 0.42 (0.90) | 0.003 |
| Cons_Specialist_Times_impu, mean (SD) | | 0.60 (1.60) | 0.46 (2.49) | 0.067 | 0.57 (1.58) | 0.46 (2.49) | 0.052 |
| Cons_Clinic_Times_impu, mean (SD) | | 1.00 (2.33) | 1.24 (5.29) | 0.059 | 0.99 (2.21) | 1.25 (5.29) | 0.062 |
| Alcohol_frequency_impu, n (%) | |  |  | 0.152 |  |  | 0.141 |
|  | 1 | 34 (9.9) | 121 (10.7) |  | 26 (10.0) | 121 (10.7) |  |
|  | 2 | 85 (24.6) | 209 (18.5) |  | 62 (23.9) | 208 (18.5) |  |
|  | 3 | 128 (37.1) | 462 (41.0) |  | 100 (38.6) | 462 (41.0) |  |
|  | 4 | 69 (20.0) | 243 (21.6) |  | 49 (18.9) | 243 (21.6) |  |
|  | 5 | 29 (8.4) | 92 (8.2) |  | 22 (8.5) | 92 (8.2) |  |
| Alcohol_units_impu, n (%) | |  |  | 0.072 |  |  | 0.084 |
|  | 0 | 37 (10.7) | 119 (10.6) |  | 27 (10.4) | 119 (10.6) |  |
|  | 1 | 175 (50.7) | 542 (48.1) |  | 127 (49.0) | 541 (48.0) |  |
|  | 2 | 101 (29.3) | 367 (32.6) |  | 77 (29.7) | 367 (32.6) |  |
|  | 3 | 32 (9.3) | 99 (8.8) |  | 28 (10.8) | 99 (8.8) |  |
| Alcohol_units_6_impu, n (%) | |  |  | 0.094 |  |  | 0.108 |
|  | 1 | 190 (55.1) | 664 (58.9) |  | 140 (54.1) | 664 (59.0) |  |
|  | 2 | 120 (34.8) | 364 (32.3) |  | 91 (35.1) | 363 (32.2) |  |
|  | 3 | 27 (7.8) | 75 (6.7) |  | 22 (8.5) | 75 (6.7) |  |
|  | 4 | 6 (1.7) | 21 (1.9) |  | 5 (1.9) | 21 (1.9) |  |
|  | 5 | 2 (0.6) | 3 (0.3) |  | 1 (0.4) | 3 (0.3) |  |
| Smoke_impu, n (%) | |  |  | 0.305 |  |  | 0.190 |
|  | 1 | 57 (16.5) | 139 (12.3) |  | 42 (16.2) | 139 (12.3) |  |
|  | 2 | 12 (3.5) | 19 (1.7) |  | 7 (2.7) | 19 (1.7) |  |
|  | 3 | 167 (48.4) | 711 (63.1) |  | 140 (54.1) | 710 (63.1) |  |
|  | 4 | 109 (31.6) | 258 (22.9) |  | 70 (27.0) | 258 (22.9) |  |
| Snuff_chewing_tobacco_impu, n (%) | |  |  | 0.054 |  |  | 0.076 |
|  | 1 | 22 (6.4) | 67 (5.9) |  | 18 (6.9) | 67 (6.0) |  |
|  | 2 | 1 (0.3) | 1 (0.1) |  | 1 (0.4) | 1 (0.1) |  |
|  | 3 | 19 (5.5) | 57 (5.1) |  | 12 (4.6) | 57 (5.1) |  |
|  | 4 | 303 (87.8) | 1002 (88.9) |  | 228 (88.0) | 1001 (88.9) |  |
| Cod_liver_oil_omega3_impu, n (%) | |  |  | 0.113 |  |  | 0.138 |
|  | 0 | 262 (75.9) | 818 (72.6) |  | 193 (74.5) | 817 (72.6) |  |
|  | 1 | 43 (12.5) | 148 (13.1) |  | 38 (14.7) | 148 (13.1) |  |
|  | 2 | 15 (4.3) | 45 (4.0) |  | 11 (4.2) | 45 (4.0) |  |
|  | 3 | 25 (7.2) | 116 (10.3) |  | 17 (6.6) | 116 (10.3) |  |
| FRUIT_UNITS_T7, mean (SD) | | 1.71 (1.34) | 1.97 (1.72) | 0.167 | 1.77 (1.42) | 1.93 (1.29) | 0.118 |
| RED_MEAT_T7, n (%) | |  |  | 0.047 |  |  | 0.041 |
|  | 1 | 23 (6.7) | 69 (6.1) |  | 16 (6.2) | 69 (6.1) |  |
|  | 2 | 77 (22.3) | 254 (22.5) |  | 55 (21.2) | 254 (22.6) |  |
|  | 3 | 218 (63.2) | 721 (64.0) |  | 167 (64.5) | 720 (63.9) |  |
|  | 4 | 24 (7.0) | 70 (6.2) |  | 18 (6.9) | 70 (6.2) |  |
|  | 5 | 3 (0.9) | 13 (1.2) |  | 3 (1.2) | 13 (1.2) |  |
| FRUITS_VEG_BERRY_T7, n (%) | |  |  | 0.108 |  |  | 0.084 |
|  | 1 | 4 (1.2) | 12 (1.1) |  | 3 (1.2) | 12 (1.1) |  |
|  | 2 | 15 (4.3) | 45 (4.0) |  | 8 (3.1) | 45 (4.0) |  |
|  | 3 | 64 (18.6) | 258 (22.9) |  | 53 (20.5) | 258 (22.9) |  |
|  | 4 | 102 (29.6) | 311 (27.6) |  | 77 (29.7) | 310 (27.5) |  |
|  | 5 | 160 (46.4) | 501 (44.5) |  | 118 (45.6) | 501 (44.5) |  |
| LEAN_FISH_T7, n (%) | |  |  | 0.225 |  |  | 0.155 |
|  | 1 | 23 (6.7) | 27 (2.4) |  | 12 (4.6) | 27 (2.4) |  |
|  | 2 | 53 (15.4) | 153 (13.6) |  | 43 (16.6) | 153 (13.6) |  |
|  | 3 | 224 (64.9) | 801 (71.1) |  | 174 (67.2) | 800 (71.0) |  |
|  | 4 | 39 (11.3) | 132 (11.7) |  | 27 (10.4) | 132 (11.7) |  |
|  | 5 | 6 (1.7) | 14 (1.2) |  | 3 (1.2) | 14 (1.2) |  |
| FAT_FISH_T7, n (%) | |  |  | 0.139 |  |  | 0.124 |
|  | 1 | 51 (14.8) | 116 (10.3) |  | 35 (13.5) | 115 (10.2) |  |
|  | 2 | 116 (33.6) | 390 (34.6) |  | 87 (33.6) | 390 (34.6) |  |
|  | 3 | 157 (45.5) | 548 (48.6) |  | 121 (46.7) | 548 (48.7) |  |
|  | 4 | 15 (4.3) | 55 (4.9) |  | 10 (3.9) | 55 (4.9) |  |
|  | 5 | 6 (1.7) | 18 (1.6) |  | 6 (2.3) | 18 (1.6) |  |
| PHYS_ACTIVITY_LEISURE_T7, n (%) | |  |  | 0.096 |  |  | 0.081 |
|  | 1 | 83 (24.1) | 229 (20.3) |  | 61 (23.6) | 228 (20.2) |  |
|  | 2 | 181 (52.5) | 626 (55.5) |  | 138 (53.3) | 626 (55.6) |  |
|  | 3 | 72 (20.9) | 247 (21.9) |  | 55 (21.2) | 247 (21.9) |  |
|  | 4 | 9 (2.6) | 25 (2.2) |  | 5 (1.9) | 25 (2.2) |  |
| EXERCISE_T7, n (%) | |  |  | 0.186 |  |  | 0.129 |
|  | 1 | 44 (12.8) | 100 (8.9) |  | 28 (10.8) | 100 (8.9) |  |
|  | 2 | 55 (15.9) | 144 (12.8) |  | 41 (15.8) | 144 (12.8) |  |
|  | 3 | 51 (14.8) | 157 (13.9) |  | 36 (13.9) | 157 (13.9) |  |
|  | 4 | 111 (32.2) | 437 (38.8) |  | 88 (34.0) | 436 (38.7) |  |
|  | 5 | 84 (24.3) | 289 (25.6) |  | 66 (25.5) | 289 (25.7) |  |
| **Dataset 10** | | | | | | | |
|  | | **Before matching** | | | **After matching** | | |
|  | | Not LLD user | LLD user | SMD | Not LLD user | LLD user | SMD |
| n | | 345 | 1127 |  | 251 | 1124 |  |
| AGE, mean (SD) | | 66.34 (13.45) | 69.41 (9.72) | 0.261 | 67.57 (12.87) | 69.39 (9.73) | 0.160 |
| SEX_T7, n (%) | |  |  | 0.390 |  |  | 0.294 |
|  | 1 | 193 (55.9) | 836 (74.2) |  | 152 (60.6) | 834 (74.2) |  |
| BMI, mean (SD) | | 28.17 (4.94) | 28.41 (4.35) | 0.050 | 28.37 (4.96) | 28.39 (4.34) | 0.005 |
| HEART_FAILURE_T7, n (%) | |  |  | 0.204 |  |  | 0.158 |
|  | 0 | 283 (82.0) | 832 (73.8) |  | 202 (80.5) | 831 (73.9) |  |
|  | 1 | 27 (7.8) | 114 (10.1) |  | 20 (8.0) | 114 (10.1) |  |
|  | 2 | 35 (10.1) | 181 (16.1) |  | 29 (11.6) | 179 (15.9) |  |
| ATRIAL_FIBRILLATION_T7, n (%) | |  |  | 0.137 |  |  | 0.041 |
|  | 0 | 239 (69.3) | 846 (75.1) |  | 186 (74.1) | 845 (75.2) |  |
|  | 1 | 63 (18.3) | 155 (13.8) |  | 38 (15.1) | 154 (13.7) |  |
|  | 2 | 43 (12.5) | 126 (11.2) |  | 27 (10.8) | 125 (11.1) |  |
| STROKE_T7, n (%) | |  |  | 0.113 |  |  | 0.126 |
|  | 2 | 37 (10.7) | 163 (14.5) |  | 26 (10.4) | 163 (14.5) |  |
| KIDNEY_DISEASE_T7, n (%) | |  |  | 0.142 |  |  | 0.139 |
|  | 0 | 281 (81.4) | 976 (86.6) |  | 205 (81.7) | 974 (86.7) |  |
|  | 1 | 37 (10.7) | 90 (8.0) |  | 26 (10.4) | 89 (7.9) |  |
|  | 2 | 27 (7.8) | 61 (5.4) |  | 20 (8.0) | 61 (5.4) |  |
| CANCER_T7, n (%) | |  |  | 0.032 |  |  | 0.019 |
|  | 0 | 279 (80.9) | 922 (81.8) |  | 204 (81.3) | 919 (81.8) |  |
|  | 1 | 25 (7.2) | 73 (6.5) |  | 16 (6.4) | 73 (6.5) |  |
|  | 2 | 41 (11.9) | 132 (11.7) |  | 31 (12.4) | 132 (11.7) |  |
| DIABETES_impu_new, n (%) | |  |  | 0.070 |  |  | 0.101 |
|  | 0 | 281 (81.4) | 893 (79.2) |  | 208 (82.9) | 892 (79.4) |  |
|  | 1 | 51 (14.8) | 195 (17.3) |  | 34 (13.5) | 193 (17.2) |  |
|  | 2 | 13 (3.8) | 39 (3.5) |  | 9 (3.6) | 39 (3.5) |  |
| Cons_GP_Times_impu, mean (SD) | | 4.18 (5.12) | 4.13 (4.51) | 0.011 | 4.15 (5.28) | 4.11 (4.49) | 0.008 |
| Cons_Emergency_Times_impu, mean (SD) | | 0.39 (0.94) | 0.30 (0.72) | 0.113 | 0.29 (0.65) | 0.30 (0.72) | 0.002 |
| Cons_Hospital_Times_impu, mean (SD) | | 0.43 (1.26) | 0.44 (0.91) | 0.002 | 0.45 (1.39) | 0.44 (0.91) | 0.013 |
| Cons_Specialist_Times_impu, mean (SD) | | 0.64 (1.80) | 0.50 (2.59) | 0.063 | 0.51 (1.38) | 0.50 (2.60) | 0.005 |
| Cons_Clinic_Times_impu, mean (SD) | | 1.44 (5.05) | 1.13 (4.25) | 0.065 | 1.19 (3.01) | 1.13 (4.26) | 0.016 |
| Alcohol_frequency_impu, n (%) | |  |  | 0.150 |  |  | 0.142 |
|  | 1 | 34 (9.9) | 121 (10.7) |  | 20 (8.0) | 121 (10.8) |  |
|  | 2 | 85 (24.6) | 209 (18.5) |  | 58 (23.1) | 208 (18.5) |  |
|  | 3 | 129 (37.4) | 462 (41.0) |  | 99 (39.4) | 461 (41.0) |  |
|  | 4 | 69 (20.0) | 243 (21.6) |  | 52 (20.7) | 243 (21.6) |  |
|  | 5 | 28 (8.1) | 92 (8.2) |  | 22 (8.8) | 91 (8.1) |  |
| Alcohol_units_impu, n (%) | |  |  | 0.075 |  |  | 0.096 |
|  | 0 | 36 (10.4) | 120 (10.6) |  | 21 (8.4) | 120 (10.7) |  |
|  | 1 | 176 (51.0) | 540 (47.9) |  | 127 (50.6) | 537 (47.8) |  |
|  | 2 | 101 (29.3) | 367 (32.6) |  | 78 (31.1) | 367 (32.7) |  |
|  | 3 | 32 (9.3) | 100 (8.9) |  | 25 (10.0) | 100 (8.9) |  |
| Alcohol_units_6_impu, n (%) | |  |  | 0.098 |  |  | 0.083 |
|  | 1 | 191 (55.4) | 664 (58.9) |  | 139 (55.4) | 663 (59.0) |  |
|  | 2 | 118 (34.2) | 363 (32.2) |  | 86 (34.3) | 361 (32.1) |  |
|  | 3 | 28 (8.1) | 74 (6.6) |  | 20 (8.0) | 74 (6.6) |  |
|  | 4 | 6 (1.7) | 23 (2.0) |  | 5 (2.0) | 23 (2.0) |  |
|  | 5 | 2 (0.6) | 3 (0.3) |  | 1 (0.4) | 3 (0.3) |  |
| Smoke_impu, n (%) | |  |  | 0.305 |  |  | 0.195 |
|  | 1 | 57 (16.5) | 139 (12.3) |  | 35 (13.9) | 139 (12.4) |  |
|  | 2 | 12 (3.5) | 19 (1.7) |  | 8 (3.2) | 19 (1.7) |  |
|  | 3 | 167 (48.4) | 711 (63.1) |  | 136 (54.2) | 709 (63.1) |  |
|  | 4 | 109 (31.6) | 258 (22.9) |  | 72 (28.7) | 257 (22.9) |  |
| Snuff_chewing_tobacco_impu, n (%) | |  |  | 0.054 |  |  | 0.069 |
|  | 1 | 22 (6.4) | 67 (5.9) |  | 12 (4.8) | 67 (6.0) |  |
|  | 2 | 1 (0.3) | 1 (0.1) |  | 0 (0.0) | 1 (0.1) |  |
|  | 3 | 19 (5.5) | 57 (5.1) |  | 12 (4.8) | 57 (5.1) |  |
|  | 4 | 303 (87.8) | 1002 (88.9) |  | 227 (90.4) | 999 (88.9) |  |
| Cod_liver_oil_omega3_impu, n (%) | |  |  | 0.098 |  |  | 0.063 |
|  | 0 | 260 (75.4) | 822 (72.9) |  | 189 (75.3) | 819 (72.9) |  |
|  | 1 | 44 (12.8) | 144 (12.8) |  | 30 (12.0) | 144 (12.8) |  |
|  | 2 | 15 (4.3) | 45 (4.0) |  | 10 (4.0) | 45 (4.0) |  |
|  | 3 | 26 (7.5) | 116 (10.3) |  | 22 (8.8) | 116 (10.3) |  |
| FRUIT_UNITS_T7, mean (SD) | | 1.75 (1.37) | 1.97 (1.71) | 0.141 | 1.81 (1.46) | 1.92 (1.25) | 0.080 |
| RED_MEAT_T7, n (%) | |  |  | 0.089 |  |  |  |
|  | 1 | 24 (7.0) | 74 (6.6) |  | 19 (7.6) | 74 (6.6) |  |
|  | 2 | 75 (21.7) | 256 (22.7) |  | 54 (21.5) | 256 (22.8) |  |
|  | 3 | 216 (62.6) | 715 (63.4) |  | 159 (63.3) | 714 (63.5) |  |
|  | 4 | 27 (7.8) | 67 (5.9) |  | 17 (6.8) | 67 (6.0) |  |
|  | 5 | 3 (0.9) | 15 (1.3) |  | 2 (0.8) | 13 (1.2) |  |
| FRUITS_VEG_BERRY_T7, n (%) | |  |  | 0.114 |  |  | 0.124 |
|  | 1 | 3 (0.9) | 12 (1.1) |  | 3 (1.2) | 12 (1.1) |  |
|  | 2 | 18 (5.2) | 47 (4.2) |  | 12 (4.8) | 47 (4.2) |  |
|  | 3 | 64 (18.6) | 257 (22.8) |  | 45 (17.9) | 257 (22.9) |  |
|  | 4 | 100 (29.0) | 310 (27.5) |  | 73 (29.1) | 309 (27.5) |  |
|  | 5 | 160 (46.4) | 501 (44.5) |  | 118 (47.0) | 499 (44.4) |  |
| LEAN_FISH_T7, n (%) | |  |  | 0.240 |  |  | 0.112 |
|  | 1 | 24 (7.0) | 27 (2.4) |  | 11 (4.4) | 27 (2.4) |  |
|  | 2 | 52 (15.1) | 153 (13.6) |  | 34 (13.5) | 153 (13.6) |  |
|  | 3 | 223 (64.6) | 803 (71.3) |  | 176 (70.1) | 800 (71.2) |  |
|  | 4 | 40 (11.6) | 133 (11.8) |  | 28 (11.2) | 133 (11.8) |  |
|  | 5 | 6 (1.7) | 11 (1.0) |  | 2 (0.8) | 11 (1.0) |  |
| FAT_FISH_T7, n (%) | |  |  | 0.159 |  |  | 0.103 |
|  | 1 | 50 (14.5) | 115 (10.2) |  | 33 (13.1) | 114 (10.1) |  |
|  | 2 | 117 (33.9) | 391 (34.7) |  | 83 (33.1) | 391 (34.8) |  |
|  | 3 | 155 (44.9) | 551 (48.9) |  | 119 (47.4) | 549 (48.8) |  |
|  | 4 | 14 (4.1) | 53 (4.7) |  | 11 (4.4) | 53 (4.7) |  |
|  | 5 | 9 (2.6) | 17 (1.5) |  | 5 (2.0) | 17 (1.5) |  |
| PHYS_ACTIVITY_LEISURE_T7, n (%) | |  |  | 0.088 |  |  | 0.055 |
|  | 1 | 84 (24.3) | 237 (21.0) |  | 51 (20.3) | 234 (20.8) |  |
|  | 2 | 178 (51.6) | 622 (55.2) |  | 137 (54.6) | 622 (55.3) |  |
|  | 3 | 74 (21.4) | 242 (21.5) |  | 55 (21.9) | 242 (21.5) |  |
|  | 4 | 9 (2.6) | 26 (2.3) |  | 8 (3.2) | 26 (2.3) |  |
| EXERCISE_T7, n (%) | |  |  | 0.176 |  |  | 0.157 |
|  | 1 | 44 (12.8) | 105 (9.3) |  | 30 (12.0) | 105 (9.3) |  |
|  | 2 | 54 (15.7) | 141 (12.5) |  | 38 (15.1) | 140 (12.5) |  |
|  | 3 | 52 (15.1) | 158 (14.0) |  | 33 (13.1) | 158 (14.1) |  |
|  | 4 | 111 (32.2) | 435 (38.6) |  | 81 (32.3) | 433 (38.5) |  |
|  | 5 | 84 (24.3) | 288 (25.6) |  | 69 (27.5) | 288 (25.6) |  |
| SMD, standardized mean difference | | | | | | | |

Additional file: Table S6. Results from propensity score matching of the ten imputed datasets for the logistic regression analysis of the association between use of antihypertensive drugs and achieving the treatment goal for blood pressure among those with self-reported hypertension

| **Dataset 1** | | | | | | | |
| --- | --- | --- | --- | --- | --- | --- | --- |
|  | | **Before matching** | | | **After matching** | | |
|  | | Not anti-hypertensive drug user | Anti-hypertensive drug user | SMD | Not anti-hypertensive drug user | Anti-hypertensive drug user | SMD |
| n | | 64 | 763 |  | 46 | 762 |  |
| AGE, mean (SD) | | 61.95 (11.84) | 69.82 (9.82) | 0.723 | 64.17 (11.06) | 69.84 (9.81) | 0.542 |
| SEX_T7, n (%) | |  |  | 0.013 |  |  | 0.113 |
|  | 1 | 43 (67.2) | 508 (66.6) |  | 33 (71.7) | 507 (66.5) |  |
| BMI, mean (SD) | | 28.99 (5.07) | 28.99 (4.54) | 0.001 | 28.53 (4.76) | 28.99 (4.55) | 0.100 |
| HEART_FAILURE_T7, n (%) | |  |  | 0.340 |  |  | 0.247 |
|  | 0 | 55 (85.9) | 559 (73.3) |  | 38 (82.6) | 559 (73.4) |  |
|  | 1 | 2 (3.1) | 72 (9.4) |  | 2 (4.3) | 72 (9.4) |  |
|  | 2 | 7 (10.9) | 132 (17.3) |  | 6 (13.0) | 131 (17.2) |  |
| ATRIAL_FIBRILLATION_T7, n (%) | |  |  | 0.216 |  |  | 0.239 |
|  | 0 | 40 (62.5) | 544 (71.3) |  | 30 (65.2) | 544 (71.4) |  |
|  | 1 | 11 (17.2) | 121 (15.9) |  | 6 (13.0) | 120 (15.7) |  |
|  | 2 | 13 (20.3) | 98 (12.8) |  | 10 (21.7) | 98 (12.9) |  |
| STROKE_T7, n (%) | |  |  | 0.135 |  |  | 0.002 |
|  | 2 | 8 (12.5) | 132 (17.3) |  | 8 (17.4) | 132 (17.3) |  |
| KIDNEY_DISEASE_T7, n (%) | |  |  | 0.235 |  |  | 0.229 |
|  | 0 | 53 (82.8) | 654 (85.7) |  | 38 (82.6) | 653 (85.7) |  |
|  | 1 | 4 (6.2) | 70 (9.2) |  | 3 (6.5) | 70 (9.2) |  |
|  | 2 | 7 (10.9) | 39 (5.1) |  | 5 (10.9) | 39 (5.1) |  |
| CANCER_T7, n (%) | |  |  | 0.205 |  |  | 0.193 |
|  | 0 | 56 (87.5) | 620 (81.3) |  | 40 (87.0) | 619 (81.2) |  |
|  | 1 | 4 (6.2) | 51 (6.7) |  | 3 (6.5) | 51 (6.7) |  |
|  | 2 | 4 (6.2) | 92 (12.1) |  | 3 (6.5) | 92 (12.1) |  |
| DIABETES_impu_new, n (%) | |  |  | 0.038 |  |  | 0.139 |
|  | 0 | 47 (73.4) | 573 (75.1) |  | 35 (76.1) | 572 (75.1) |  |
|  | 1 | 13 (20.3) | 145 (19.0) |  | 7 (15.2) | 145 (19.0) |  |
|  | 2 | 4 (6.2) | 45 (5.9) |  | 4 (8.7) | 45 (5.9) |  |
| Cons_GP_Times_impu, mean (SD) | | 3.66 (3.32) | 5.11 (6.78) | 0.271 | 3.61 (2.84) | 5.11 (6.79) | 0.288 |
| Cons_Emergency_Times_impu, mean (SD) | | 0.33 (0.64) | 0.34 (0.80) | 0.023 | 0.26 (0.57) | 0.35 (0.80) | 0.121 |
| Cons_Hospital_Times_impu, mean (SD) | | 0.50 (1.62) | 0.47 (1.01) | 0.024 | 0.28 (0.58) | 0.47 (1.01) | 0.226 |
| Cons_Specialist_Times_impu, mean (SD) | | 0.48 (0.96) | 0.56 (3.04) | 0.035 | 0.54 (1.03) | 0.56 (3.04) | 0.009 |
| Cons_Clinic_Times_impu, mean (SD) | | 0.73 (1.34) | 1.20 (3.54) | 0.174 | 0.93 (1.51) | 1.20 (3.54) | 0.098 |
| Alcohol_frequency_impu, n (%) | |  |  | 0.160 |  |  | 0.283 |
|  | 1 | 7 (10.9) | 79 (10.4) |  | 2 (4.3) | 79 (10.4) |  |
|  | 2 | 14 (21.9) | 160 (21.0) |  | 10 (21.7) | 160 (21.0) |  |
|  | 3 | 26 (40.6) | 285 (37.4) |  | 18 (39.1) | 284 (37.3) |  |
|  | 4 | 10 (15.6) | 166 (21.8) |  | 9 (19.6) | 166 (21.8) |  |
|  | 5 | 7 (10.9) | 73 (9.6) |  | 7 (15.2) | 73 (9.6) |  |
| Alcohol_units_impu, n (%) | |  |  | 0.203 |  |  | 0.304 |
|  | 0 | 8 (12.5) | 81 (10.6) |  | 2 (4.3) | 81 (10.6) |  |
|  | 1 | 28 (43.8) | 372 (48.8) |  | 23 (50.0) | 372 (48.8) |  |
|  | 2 | 24 (37.5) | 233 (30.5) |  | 18 (39.1) | 232 (30.4) |  |
|  | 3 | 4 (6.2) | 77 (10.1) |  | 3 (6.5) | 77 (10.1) |  |
| Alcohol_units_6_impu, n (%) | |  |  | 0.296 |  |  | 0.290 |
|  | 1 | 33 (51.6) | 448 (58.7) |  | 23 (50.0) | 447 (58.7) |  |
|  | 2 | 27 (42.2) | 237 (31.1) |  | 20 (43.5) | 237 (31.1) |  |
|  | 3 | 2 (3.1) | 55 (7.2) |  | 2 (4.3) | 55 (7.2) |  |
|  | 4 | 1 (1.6) | 18 (2.4) |  | 1 (2.2) | 18 (2.4) |  |
|  | 5 | 1 (1.6) | 5 (0.7) |  | 0 (0.0) | 5 (0.7) |  |
| Smoke_impu, n (%) | |  |  | 0.230 |  |  | 0.255 |
|  | 1 | 4 (6.2) | 86 (11.3) |  | 3 (6.5) | 86 (11.3) |  |
|  | 2 | 1 (1.6) | 12 (1.6) |  | 0 (0.0) | 12 (1.6) |  |
|  | 3 | 37 (57.8) | 465 (60.9) |  | 29 (63.0) | 464 (60.9) |  |
|  | 4 | 22 (34.4) | 200 (26.2) |  | 14 (30.4) | 200 (26.2) |  |
| Snuff_chewing_tobacco_impu, n (%) | |  |  | 0.098 |  |  | 0.072 |
|  | 1 | 5 (7.8) | 44 (5.8) |  | 3 (6.5) | 43 (5.6) |  |
|  | 2 | 0 (0.0) | 0 (0.0) |  | 0 (0.0) | 0 (0.0) |  |
|  | 3 | 4 (6.2) | 39 (5.1) |  | 3 (6.5) | 39 (5.1) |  |
|  | 4 | 55 (85.9) | 680 (89.1) |  | 40 (87.0) | 680 (89.2) |  |
| Cod_liver_oil_omega3_impu, n (%) | |  |  | 0.239 |  |  | 0.141 |
|  | 0 | 41 (64.1) | 560 (73.4) |  | 31 (67.4) | 560 (73.5) |  |
|  | 1 | 11 (17.2) | 101 (13.2) |  | 8 (17.4) | 101 (13.3) |  |
|  | 2 | 5 (7.8) | 27 (3.5) |  | 2 (4.3) | 26 (3.4) |  |
|  | 3 | 7 (10.9) | 75 (9.8) |  | 5 (10.9) | 75 (9.8) |  |
| FRUIT_UNITS_T7, mean (SD) | | 1.81 (1.37) | 2.06 (1.94) | 0.144 | 2.04 (1.48) | 2.05 (1.94) | 0.005 |
| RED_MEAT_T7, n (%) | |  |  | 0.438 |  |  | 0.399 |
|  | 1 | 9 (14.1) | 42 (5.5) |  | 7 (15.2) | 42 (5.5) |  |
|  | 2 | 14 (21.9) | 163 (21.4) |  | 11 (23.9) | 163 (21.4) |  |
|  | 3 | 32 (50.0) | 510 (66.8) |  | 25 (54.3) | 509 (66.8) |  |
|  | 4 | 7 (10.9) | 37 (4.8) |  | 3 (6.5) | 37 (4.9) |  |
|  | 5 | 2 (3.1) | 11 (1.4) |  | 0 (0.0) | 11 (1.4) |  |
| FRUITS_VEG_BERRY_T7, n (%) | |  |  | 0.253 |  |  | 0.201 |
|  | 1 | 0 (0.0) | 8 (1.0) |  | 0 (0.0) | 8 (1.0) |  |
|  | 2 | 5 (7.8) | 31 (4.1) |  | 2 (4.3) | 31 (4.1) |  |
|  | 3 | 12 (18.8) | 151 (19.8) |  | 8 (17.4) | 151 (19.8) |  |
|  | 4 | 14 (21.9) | 212 (27.8) |  | 11 (23.9) | 212 (27.8) |  |
|  | 5 | 33 (51.6) | 361 (47.3) |  | 25 (54.3) | 360 (47.2) |  |
| LEAN_FISH_T7, n (%) | |  |  | 0.406 |  |  | 0.299 |
|  | 1 | 7 (10.9) | 20 (2.6) |  | 3 (6.5) | 20 (2.6) |  |
|  | 2 | 8 (12.5) | 100 (13.1) |  | 7 (15.2) | 100 (13.1) |  |
|  | 3 | 43 (67.2) | 531 (69.6) |  | 32 (69.6) | 530 (69.6) |  |
|  | 4 | 4 (6.2) | 99 (13.0) |  | 4 (8.7) | 99 (13.0) |  |
|  | 5 | 2 (3.1) | 13 (1.7) |  | 0 (0.0) | 13 (1.7) |  |
| FAT_FISH_T7, n (%) | |  |  | 0.271 |  |  | 0.153 |
|  | 1 | 8 (12.5) | 87 (11.4) |  | 6 (13.0) | 87 (11.4) |  |
|  | 2 | 26 (40.6) | 261 (34.2) |  | 18 (39.1) | 260 (34.1) |  |
|  | 3 | 25 (39.1) | 368 (48.2) |  | 19 (41.3) | 368 (48.3) |  |
|  | 4 | 2 (3.1) | 36 (4.7) |  | 2 (4.3) | 36 (4.7) |  |
|  | 5 | 3 (4.7) | 11 (1.4) |  | 1 (2.2) | 11 (1.4) |  |
| PHYS_ACTIVITY_LEISURE_T7, n (%) | |  |  | 0.295 |  |  | 0.194 |
|  | 1 | 14 (21.9) | 169 (22.1) |  | 8 (17.4) | 169 (22.2) |  |
|  | 2 | 37 (57.8) | 409 (53.6) |  | 27 (58.7) | 409 (53.7) |  |
|  | 3 | 9 (14.1) | 170 (22.3) |  | 9 (19.6) | 169 (22.2) |  |
|  | 4 | 4 (6.2) | 15 (2.0) |  | 2 (4.3) | 15 (2.0) |  |
| EXERCISE_T7, n (%) | |  |  | 0.217 |  |  | 0.443 |
|  | 1 | 6 (9.4) | 88 (11.5) |  | 1 (2.2) | 88 (11.5) |  |
|  | 2 | 10 (15.6) | 90 (11.8) |  | 9 (19.6) | 90 (11.8) |  |
|  | 3 | 11 (17.2) | 123 (16.1) |  | 6 (13.0) | 123 (16.1) |  |
|  | 4 | 18 (28.1) | 276 (36.2) |  | 16 (34.8) | 276 (36.2) |  |
|  | 5 | 19 (29.7) | 186 (24.4) |  | 14 (30.4) | 185 (24.3) |  |
| **Dataset 2** | | | | | | | |
|  | | **Before matching** | | | **After matching** | | |
|  | | Not anti-hypertensive drug user | Anti-hypertensive drug user | SMD | Not anti-hypertensive drug user | Anti-hypertensive drug user | SMD |
| n | | 64 | 763 |  | 48 | 762 |  |
| AGE, mean (SD) | | 61.95 (11.84) | 69.82 (9.82) | 0.723 | 65.77 (10.39) | 69.84 (9.81) | 0.402 |
| SEX_T7, n (%) | |  |  | 0.013 |  |  | 0.041 |
|  | 1 | 43 (67.2) | 508 (66.6) |  | 31 (64.6) | 507 (66.5) |  |
| BMI, mean (SD) | | 28.99 (5.07) | 28.99 (4.54) | 0.001 | 28.83 (4.63) | 28.99 (4.54) | 0.035 |
| HEART_FAILURE_T7, n (%) | |  |  | 0.389 |  |  | 0.356 |
|  | 0 | 54 (84.4) | 563 (73.8) |  | 40 (83.3) | 562 (73.8) |  |
|  | 1 | 1 (1.6) | 79 (10.4) |  | 1 (2.1) | 79 (10.4) |  |
|  | 2 | 9 (14.1) | 121 (15.9) |  | 7 (14.6) | 121 (15.9) |  |
| ATRIAL_FIBRILLATION_T7, n (%) | |  |  | 0.266 |  |  | 0.185 |
|  | 0 | 39 (60.9) | 546 (71.6) |  | 31 (64.6) | 546 (71.7) |  |
|  | 1 | 11 (17.2) | 122 (16.0) |  | 8 (16.7) | 122 (16.0) |  |
|  | 2 | 14 (21.9) | 95 (12.5) |  | 9 (18.8) | 94 (12.3) |  |
| STROKE_T7, n (%) | |  |  | 0.089 |  |  | 0.075 |
|  | 2 | 9 (14.1) | 132 (17.3) |  | 7 (14.6) | 132 (17.3) |  |
| KIDNEY_DISEASE_T7, n (%) | |  |  | 0.180 |  |  | 0.013 |
|  | 0 | 53 (82.8) | 655 (85.8) |  | 41 (85.4) | 654 (85.8) |  |
|  | 1 | 4 (6.2) | 61 (8.0) |  | 4 (8.3) | 61 (8.0) |  |
|  | 2 | 7 (10.9) | 47 (6.2) |  | 3 (6.2) | 47 (6.2) |  |
| CANCER_T7, n (%) | |  |  | 0.179 |  |  | 0.208 |
|  | 0 | 56 (87.5) | 618 (81.0) |  | 42 (87.5) | 617 (81.0) |  |
|  | 1 | 3 (4.7) | 53 (6.9) |  | 3 (6.2) | 53 (7.0) |  |
|  | 2 | 5 (7.8) | 92 (12.1) |  | 3 (6.2) | 92 (12.1) |  |
| DIABETES_impu_new, n (%) | |  |  | 0.075 |  |  | 0.027 |
|  | 0 | 47 (73.4) | 575 (75.4) |  | 36 (75.0) | 574 (75.3) |  |
|  | 1 | 13 (20.3) | 153 (20.1) |  | 10 (20.8) | 153 (20.1) |  |
|  | 2 | 4 (6.2) | 35 (4.6) |  | 2 (4.2) | 35 (4.6) |  |
| Cons_GP_Times_impu, mean (SD) | | 3.56 (3.00) | 4.78 (5.61) | 0.271 | 3.83 (3.11) | 4.78 (5.62) | 0.208 |
| Cons_Emergency_Times_impu, mean (SD) | | 0.31 (0.59) | 0.34 (0.79) | 0.042 | 0.31 (0.59) | 0.34 (0.79) | 0.043 |
| Cons_Hospital_Times_impu, mean (SD) | | 0.48 (1.61) | 0.47 (1.01) | 0.012 | 0.50 (1.77) | 0.47 (1.01) | 0.022 |
| Cons_Specialist_Times_impu, mean (SD) | | 0.53 (0.98) | 0.52 (2.94) | 0.004 | 0.65 (1.08) | 0.52 (2.94) | 0.056 |
| Cons_Clinic_Times_impu, mean (SD) | | 1.03 (2.30) | 1.17 (3.51) | 0.045 | 1.33 (2.58) | 1.17 (3.51) | 0.054 |
| Alcohol_frequency_impu, n (%) | |  |  | 0.180 |  |  | 0.307 |
|  | 1 | 6 (9.4) | 79 (10.4) |  | 2 (4.2) | 79 (10.4) |  |
|  | 2 | 14 (21.9) | 160 (21.0) |  | 10 (20.8) | 160 (21.0) |  |
|  | 3 | 26 (40.6) | 285 (37.4) |  | 22 (45.8) | 284 (37.3) |  |
|  | 4 | 10 (15.6) | 166 (21.8) |  | 8 (16.7) | 166 (21.8) |  |
|  | 5 | 8 (12.5) | 73 (9.6) |  | 6 (12.5) | 73 (9.6) |  |
| Alcohol_units_impu, n (%) | |  |  | 0.214 |  |  | 0.283 |
|  | 0 | 7 (10.9) | 81 (10.6) |  | 3 (6.2) | 81 (10.6) |  |
|  | 1 | 28 (43.8) | 371 (48.6) |  | 22 (45.8) | 371 (48.7) |  |
|  | 2 | 25 (39.1) | 233 (30.5) |  | 20 (41.7) | 232 (30.4) |  |
|  | 3 | 4 (6.2) | 78 (10.2) |  | 3 (6.2) | 78 (10.2) |  |
| Alcohol_units_6_impu, n (%) | |  |  | 0.296 |  |  | 0.391 |
|  | 1 | 34 (53.1) | 449 (58.8) |  | 27 (56.2) | 449 (58.9) |  |
|  | 2 | 27 (42.2) | 237 (31.1) |  | 20 (41.7) | 236 (31.0) |  |
|  | 3 | 2 (3.1) | 55 (7.2) |  | 1 (2.1) | 55 (7.2) |  |
|  | 4 | 1 (1.6) | 18 (2.4) |  | 0 (0.0) | 18 (2.4) |  |
|  | 5 | 0 (0.0) | 4 (0.5) |  | 0 (0.0) | 4 (0.5) |  |
| Smoke_impu, n (%) | |  |  | 0.230 |  |  | 0.243 |
|  | 1 | 4 (6.2) | 86 (11.3) |  | 4 (8.3) | 86 (11.3) |  |
|  | 2 | 1 (1.6) | 12 (1.6) |  | 0 (0.0) | 12 (1.6) |  |
|  | 3 | 37 (57.8) | 465 (60.9) |  | 28 (58.3) | 464 (60.9) |  |
|  | 4 | 22 (34.4) | 200 (26.2) |  | 16 (33.3) | 200 (26.2) |  |
| Snuff_chewing_tobacco_impu, n (%) | |  |  | 0.095 |  |  | 0.126 |
|  | 1 | 5 (7.8) | 44 (5.8) |  | 3 (6.2) | 44 (5.8) |  |
|  | 2 | 0 (0.0) | 0 (0.0) |  | 0 (0.0) | 0 (0.0) |  |
|  | 3 | 4 (6.2) | 40 (5.2) |  | 4 (8.3) | 40 (5.2) |  |
|  | 4 | 55 (85.9) | 679 (89.0) |  | 41 (85.4) | 678 (89.0) |  |
| Cod_liver_oil_omega3_impu, n (%) | |  |  | 0.221 |  |  | 0.155 |
|  | 0 | 41 (64.1) | 557 (73.0) |  | 33 (68.8) | 557 (73.1) |  |
|  | 1 | 11 (17.2) | 102 (13.4) |  | 9 (18.8) | 101 (13.3) |  |
|  | 2 | 5 (7.8) | 30 (3.9) |  | 2 (4.2) | 30 (3.9) |  |
|  | 3 | 7 (10.9) | 74 (9.7) |  | 4 (8.3) | 74 (9.7) |  |
| FRUIT_UNITS_T7, mean (SD) | | 1.83 (1.44) | 2.09 (1.97) | 0.151 | 1.92 (1.57) | 2.08 (1.96) | 0.093 |
| RED_MEAT_T7, n (%) | |  |  | 0.465 |  |  | 0.435 |
|  | 1 | 9 (14.1) | 44 (5.8) |  | 7 (14.6) | 44 (5.8) |  |
|  | 2 | 15 (23.4) | 166 (21.8) |  | 15 (31.2) | 166 (21.8) |  |
|  | 3 | 32 (50.0) | 506 (66.3) |  | 24 (50.0) | 505 (66.3) |  |
|  | 4 | 8 (12.5) | 38 (5.0) |  | 2 (4.2) | 38 (5.0) |  |
|  | 5 | 0 (0.0) | 9 (1.2) |  | 0 (0.0) | 9 (1.2) |  |
| FRUITS_VEG_BERRY_T7, n (%) | |  |  | 0.206 |  |  | 0.205 |
|  | 1 | 0 (0.0) | 8 (1.0) |  | 0 (0.0) | 8 (1.0) |  |
|  | 2 | 4 (6.2) | 32 (4.2) |  | 3 (6.2) | 32 (4.2) |  |
|  | 3 | 12 (18.8) | 152 (19.9) |  | 10 (20.8) | 152 (19.9) |  |
|  | 4 | 15 (23.4) | 213 (27.9) |  | 11 (22.9) | 213 (28.0) |  |
|  | 5 | 33 (51.6) | 358 (46.9) |  | 24 (50.0) | 357 (46.9) |  |
| LEAN_FISH_T7, n (%) | |  |  | 0.396 |  |  | 0.255 |
|  | 1 | 7 (10.9) | 21 (2.8) |  | 3 (6.2) | 20 (2.6) |  |
|  | 2 | 9 (14.1) | 103 (13.5) |  | 7 (14.6) | 103 (13.5) |  |
|  | 3 | 43 (67.2) | 531 (69.6) |  | 33 (68.8) | 531 (69.7) |  |
|  | 4 | 5 (7.8) | 97 (12.7) |  | 5 (10.4) | 97 (12.7) |  |
|  | 5 | 0 (0.0) | 11 (1.4) |  | 0 (0.0) | 11 (1.4) |  |
| FAT_FISH_T7, n (%) | |  |  | 0.293 |  |  | 0.344 |
|  | 1 | 9 (14.1) | 88 (11.5) |  | 7 (14.6) | 87 (11.4) |  |
|  | 2 | 28 (43.8) | 261 (34.2) |  | 22 (45.8) | 261 (34.3) |  |
|  | 3 | 25 (39.1) | 367 (48.1) |  | 18 (37.5) | 367 (48.2) |  |
|  | 4 | 1 (1.6) | 38 (5.0) |  | 1 (2.1) | 38 (5.0) |  |
|  | 5 | 1 (1.6) | 9 (1.2) |  | 0 (0.0) | 9 (1.2) |  |
| PHYS_ACTIVITY_LEISURE_T7, n (%) | |  |  | 0.279 |  |  | 0.163 |
|  | 1 | 14 (21.9) | 176 (23.1) |  | 12 (25.0) | 176 (23.1) |  |
|  | 2 | 37 (57.8) | 404 (52.9) |  | 26 (54.2) | 404 (53.0) |  |
|  | 3 | 9 (14.1) | 166 (21.8) |  | 8 (16.7) | 165 (21.7) |  |
|  | 4 | 4 (6.2) | 17 (2.2) |  | 2 (4.2) | 17 (2.2) |  |
| EXERCISE_T7, n (%) | |  |  | 0.213 |  |  | 0.348 |
|  | 1 | 6 (9.4) | 91 (11.9) |  | 3 (6.2) | 91 (11.9) |  |
|  | 2 | 10 (15.6) | 92 (12.1) |  | 10 (20.8) | 92 (12.1) |  |
|  | 3 | 12 (18.8) | 122 (16.0) |  | 8 (16.7) | 122 (16.0) |  |
|  | 4 | 18 (28.1) | 274 (35.9) |  | 13 (27.1) | 274 (36.0) |  |
|  | 5 | 18 (28.1) | 184 (24.1) |  | 14 (29.2) | 183 (24.0) |  |
| **Dataset 3** | | | | | | | |
|  | | **Before matching** | | | **After matching** | | |
|  | | Not anti-hypertensive drug user | Anti-hypertensive drug user | SMD | Not anti-hypertensive drug user | Anti-hypertensive drug user | SMD |
| n | | 64 | 763 |  | 46 | 760 |  |
| AGE, mean (SD) | | 61.95 (11.84) | 69.82 (9.82) | 0.723 | 65.02 (11.05) | 69.84 (9.82) | 0.461 |
| SEX_T7, n (%) | |  |  | 0.013 |  |  | 0.064 |
|  | 1 | 43 (67.2) | 508 (66.6) |  | 32 (69.6) | 506 (66.6) |  |
| BMI, mean (SD) | | 28.99 (5.07) | 29.01 (4.55) | 0.003 | 28.88 (4.63) | 29.02 (4.55) | 0.030 |
| HEART_FAILURE_T7, n (%) | |  |  | 0.344 |  |  | 0.312 |
|  | 0 | 54 (84.4) | 560 (73.4) |  | 39 (84.8) | 557 (73.3) |  |
|  | 1 | 2 (3.1) | 87 (11.4) |  | 2 (4.3) | 87 (11.4) |  |
|  | 2 | 8 (12.5) | 116 (15.2) |  | 5 (10.9) | 116 (15.3) |  |
| ATRIAL_FIBRILLATION_T7, n (%) | |  |  | 0.228 |  |  | 0.263 |
|  | 0 | 40 (62.5) | 541 (70.9) |  | 29 (63.0) | 540 (71.1) |  |
|  | 1 | 11 (17.2) | 129 (16.9) |  | 7 (15.2) | 129 (17.0) |  |
|  | 2 | 13 (20.3) | 93 (12.2) |  | 10 (21.7) | 91 (12.0) |  |
| STROKE_T7, n (%) | |  |  | 0.093 |  |  | 0.124 |
|  | 2 | 9 (14.1) | 133 (17.4) |  | 6 (13.0) | 133 (17.5) |  |
| KIDNEY_DISEASE_T7, n (%) | |  |  | 0.169 |  |  | 0.061 |
|  | 0 | 53 (82.8) | 654 (85.7) |  | 39 (84.8) | 651 (85.7) |  |
|  | 1 | 5 (7.8) | 70 (9.2) |  | 4 (8.7) | 70 (9.2) |  |
|  | 2 | 6 (9.4) | 39 (5.1) |  | 3 (6.5) | 39 (5.1) |  |
| CANCER_T7, n (%) | |  |  | 0.177 |  |  | 0.307 |
|  | 0 | 56 (87.5) | 619 (81.1) |  | 42 (91.3) | 616 (81.1) |  |
|  | 1 | 3 (4.7) | 58 (7.6) |  | 2 (4.3) | 58 (7.6) |  |
|  | 2 | 5 (7.8) | 86 (11.3) |  | 2 (4.3) | 86 (11.3) |  |
| DIABETES_impu_new, n (%) | |  |  | 0.075 |  |  | 0.160 |
|  | 0 | 47 (73.4) | 574 (75.2) |  | 37 (80.4) | 571 (75.1) |  |
|  | 1 | 13 (20.3) | 154 (20.2) |  | 8 (17.4) | 154 (20.3) |  |
|  | 2 | 4 (6.2) | 35 (4.6) |  | 1 (2.2) | 35 (4.6) |  |
| Cons_GP_Times_impu, mean (SD) | | 4.86 (8.57) | 4.91 (5.79) | 0.007 | 5.52 (9.89) | 4.91 (5.79) | 0.076 |
| Cons_Emergency_Times_impu, mean (SD) | | 0.34 (0.74) | 0.34 (0.78) | 0.009 | 0.35 (0.77) | 0.34 (0.78) | 0.012 |
| Cons_Hospital_Times_impu, mean (SD) | | 0.47 (1.61) | 0.48 (1.01) | 0.006 | 0.52 (1.83) | 0.48 (1.02) | 0.030 |
| Cons_Specialist_Times_impu, mean (SD) | | 0.56 (1.18) | 0.56 (3.11) | 0.001 | 0.74 (1.34) | 0.56 (3.12) | 0.074 |
| Cons_Clinic_Times_impu, mean (SD) | | 1.06 (2.35) | 1.18 (3.60) | 0.037 | 1.28 (2.67) | 1.18 (3.61) | 0.033 |
| Alcohol_frequency_impu, n (%) | |  |  | 0.169 |  |  | 0.283 |
|  | 1 | 6 (9.4) | 79 (10.4) |  | 2 (4.3) | 79 (10.4) |  |
|  | 2 | 15 (23.4) | 160 (21.0) |  | 11 (23.9) | 159 (20.9) |  |
|  | 3 | 26 (40.6) | 285 (37.4) |  | 19 (41.3) | 283 (37.2) |  |
|  | 4 | 10 (15.6) | 166 (21.8) |  | 8 (17.4) | 166 (21.8) |  |
|  | 5 | 7 (10.9) | 73 (9.6) |  | 6 (13.0) | 73 (9.6) |  |
| Alcohol_units_impu, n (%) | |  |  | 0.203 |  |  | 0.142 |
|  | 0 | 8 (12.5) | 81 (10.6) |  | 4 (8.7) | 80 (10.5) |  |
|  | 1 | 28 (43.8) | 372 (48.8) |  | 21 (45.7) | 371 (48.8) |  |
|  | 2 | 24 (37.5) | 233 (30.5) |  | 17 (37.0) | 232 (30.5) |  |
|  | 3 | 4 (6.2) | 77 (10.1) |  | 4 (8.7) | 77 (10.1) |  |
| Alcohol_units_6_impu, n (%) | |  |  | 0.297 |  |  | 0.317 |
|  | 1 | 34 (53.1) | 448 (58.7) |  | 25 (54.3) | 446 (58.7) |  |
|  | 2 | 27 (42.2) | 237 (31.1) |  | 19 (41.3) | 236 (31.1) |  |
|  | 3 | 2 (3.1) | 55 (7.2) |  | 1 (2.2) | 55 (7.2) |  |
|  | 4 | 1 (1.6) | 19 (2.5) |  | 1 (2.2) | 19 (2.5) |  |
|  | 5 | 0 (0.0) | 4 (0.5) |  | 0 (0.0) | 4 (0.5) |  |
| Smoke_impu, n (%) | |  |  | 0.230 |  |  | 0.213 |
|  | 1 | 4 (6.2) | 86 (11.3) |  | 4 (8.7) | 86 (11.3) |  |
|  | 2 | 1 (1.6) | 12 (1.6) |  | 0 (0.0) | 12 (1.6) |  |
|  | 3 | 37 (57.8) | 465 (60.9) |  | 28 (60.9) | 463 (60.9) |  |
|  | 4 | 22 (34.4) | 200 (26.2) |  | 14 (30.4) | 199 (26.2) |  |
| Snuff_chewing_tobacco_impu, n (%) | |  |  | 0.098 |  |  | 0.152 |
|  | 1 | 5 (7.8) | 44 (5.8) |  | 2 (4.3) | 44 (5.8) |  |
|  | 2 | 0 (0.0) | 0 (0.0) |  | 0 (0.0) | 0 (0.0) |  |
|  | 3 | 4 (6.2) | 39 (5.1) |  | 4 (8.7) | 39 (5.1) |  |
|  | 4 | 55 (85.9) | 680 (89.1) |  | 40 (87.0) | 677 (89.1) |  |
| Cod_liver_oil_omega3_impu, n (%) | |  |  | 0.168 |  |  | 0.251 |
|  | 0 | 42 (65.6) | 559 (73.3) |  | 31 (67.4) | 558 (73.4) |  |
|  | 1 | 11 (17.2) | 99 (13.0) |  | 10 (21.7) | 98 (12.9) |  |
|  | 2 | 3 (4.7) | 30 (3.9) |  | 1 (2.2) | 30 (3.9) |  |
|  | 3 | 8 (12.5) | 75 (9.8) |  | 4 (8.7) | 74 (9.7) |  |
| FRUIT_UNITS_T7, mean (SD) | | 1.80 (1.38) | 2.06 (1.94) | 0.155 | 1.89 (1.51) | 2.05 (1.94) | 0.093 |
| RED_MEAT_T7, n (%) | |  |  | 0.452 |  |  | 0.346 |
|  | 1 | 9 (14.1) | 45 (5.9) |  | 7 (15.2) | 45 (5.9) |  |
|  | 2 | 14 (21.9) | 162 (21.2) |  | 10 (21.7) | 162 (21.3) |  |
|  | 3 | 33 (51.6) | 509 (66.7) |  | 27 (58.7) | 506 (66.6) |  |
|  | 4 | 8 (12.5) | 38 (5.0) |  | 2 (4.3) | 38 (5.0) |  |
|  | 5 | 0 (0.0) | 9 (1.2) |  | 0 (0.0) | 9 (1.2) |  |
| FRUITS_VEG_BERRY_T7, n (%) | |  |  | 0.221 |  |  | 0.220 |
|  | 1 | 0 (0.0) | 8 (1.0) |  | 0 (0.0) | 8 (1.1) |  |
|  | 2 | 5 (7.8) | 35 (4.6) |  | 3 (6.5) | 35 (4.6) |  |
|  | 3 | 12 (18.8) | 151 (19.8) |  | 10 (21.7) | 151 (19.9) |  |
|  | 4 | 15 (23.4) | 213 (27.9) |  | 10 (21.7) | 213 (28.0) |  |
|  | 5 | 32 (50.0) | 356 (46.7) |  | 23 (50.0) | 353 (46.4) |  |
| LEAN_FISH_T7, n (%) | |  |  | 0.411 |  |  | 0.212 |
|  | 1 | 7 (10.9) | 19 (2.5) |  | 1 (2.2) | 18 (2.4) |  |
|  | 2 | 9 (14.1) | 103 (13.5) |  | 8 (17.4) | 103 (13.6) |  |
|  | 3 | 43 (67.2) | 532 (69.7) |  | 32 (69.6) | 530 (69.7) |  |
|  | 4 | 5 (7.8) | 97 (12.7) |  | 5 (10.9) | 97 (12.8) |  |
|  | 5 | 0 (0.0) | 12 (1.6) |  | 0 (0.0) | 12 (1.6) |  |
| FAT_FISH_T7, n (%) | |  |  | 0.297 |  |  | 0.240 |
|  | 1 | 8 (12.5) | 87 (11.4) |  | 7 (15.2) | 86 (11.3) |  |
|  | 2 | 29 (45.3) | 261 (34.2) |  | 18 (39.1) | 259 (34.1) |  |
|  | 3 | 25 (39.1) | 369 (48.4) |  | 19 (41.3) | 369 (48.6) |  |
|  | 4 | 1 (1.6) | 37 (4.8) |  | 1 (2.2) | 37 (4.9) |  |
|  | 5 | 1 (1.6) | 9 (1.2) |  | 1 (2.2) | 9 (1.2) |  |
| PHYS_ACTIVITY_LEISURE_T7, n (%) | |  |  | 0.291 |  |  | 0.168 |
|  | 1 | 14 (21.9) | 178 (23.3) |  | 11 (23.9) | 178 (23.4) |  |
|  | 2 | 37 (57.8) | 404 (52.9) |  | 25 (54.3) | 402 (52.9) |  |
|  | 3 | 9 (14.1) | 166 (21.8) |  | 8 (17.4) | 165 (21.7) |  |
|  | 4 | 4 (6.2) | 15 (2.0) |  | 2 (4.3) | 15 (2.0) |  |
| EXERCISE_T7, n (%) | |  |  | 0.189 |  |  | 0.225 |
|  | 1 | 7 (10.9) | 90 (11.8) |  | 4 (8.7) | 90 (11.8) |  |
|  | 2 | 10 (15.6) | 91 (11.9) |  | 9 (19.6) | 91 (12.0) |  |
|  | 3 | 11 (17.2) | 121 (15.9) |  | 7 (15.2) | 121 (15.9) |  |
|  | 4 | 18 (28.1) | 274 (35.9) |  | 15 (32.6) | 274 (36.1) |  |
|  | 5 | 18 (28.1) | 187 (24.5) |  | 11 (23.9) | 184 (24.2) |  |
| **Dataset 4** | | | | | | | |
|  | | **Before matching** | | | **After matching** | | |
|  | | Not anti-hypertensive drug user | Anti-hypertensive drug user | SMD | Not anti-hypertensive drug user | Anti-hypertensive drug user | SMD |
| n | | 64 | 763 |  | 45 | 761 |  |
| AGE, mean (SD) | | 61.95 (11.84) | 69.82 (9.82) | 0.723 | 63.98 (10.44) | 69.85 (9.80) | 0.580 |
| SEX_T7, n (%) | |  |  | 0.013 |  |  | 0.043 |
|  | 1 | 43 (67.2) | 508 (66.6) |  | 29 (64.4) | 506 (66.5) |  |
| BMI, mean (SD) | | 28.99 (5.07) | 29.01 (4.55) | 0.004 | 28.73 (4.66) | 29.01 (4.54) | 0.060 |
| HEART_FAILURE_T7, n (%) | |  |  | 0.301 |  |  | 0.324 |
|  | 0 | 54 (84.4) | 567 (74.3) |  | 37 (82.2) | 566 (74.4) |  |
|  | 1 | 2 (3.1) | 74 (9.7) |  | 1 (2.2) | 74 (9.7) |  |
|  | 2 | 8 (12.5) | 122 (16.0) |  | 7 (15.6) | 121 (15.9) |  |
| ATRIAL_FIBRILLATION_T7, n (%) | |  |  | 0.248 |  |  | 0.221 |
|  | 0 | 39 (60.9) | 545 (71.4) |  | 30 (66.7) | 545 (71.6) |  |
|  | 1 | 12 (18.8) | 125 (16.4) |  | 6 (13.3) | 124 (16.3) |  |
|  | 2 | 13 (20.3) | 93 (12.2) |  | 9 (20.0) | 92 (12.1) |  |
| STROKE_T7, n (%) | |  |  | 0.086 |  |  | 0.041 |
|  | 2 | 9 (14.1) | 131 (17.2) |  | 7 (15.6) | 130 (17.1) |  |
| KIDNEY_DISEASE_T7, n (%) | |  |  | 0.230 |  |  | 0.040 |
|  | 0 | 53 (82.8) | 653 (85.6) |  | 39 (86.7) | 651 (85.5) |  |
|  | 1 | 4 (6.2) | 70 (9.2) |  | 4 (8.9) | 70 (9.2) |  |
|  | 2 | 7 (10.9) | 40 (5.2) |  | 2 (4.4) | 40 (5.3) |  |
| CANCER_T7, n (%) | |  |  | 0.205 |  |  | 0.187 |
|  | 0 | 56 (87.5) | 620 (81.3) |  | 39 (86.7) | 619 (81.3) |  |
|  | 1 | 4 (6.2) | 51 (6.7) |  | 3 (6.7) | 50 (6.6) |  |
|  | 2 | 4 (6.2) | 92 (12.1) |  | 3 (6.7) | 92 (12.1) |  |
| DIABETES_impu_new, n (%) | |  |  | 0.067 |  |  | 0.176 |
|  | 0 | 47 (73.4) | 577 (75.6) |  | 36 (80.0) | 576 (75.7) |  |
|  | 1 | 13 (20.3) | 149 (19.5) |  | 6 (13.3) | 148 (19.4) |  |
|  | 2 | 4 (6.2) | 37 (4.8) |  | 3 (6.7) | 37 (4.9) |  |
| Cons_GP_Times_impu, mean (SD) | | 3.72 (3.33) | 4.95 (6.09) | 0.250 | 3.91 (3.36) | 4.94 (6.09) | 0.208 |
| Cons_Emergency_Times_impu, mean (SD) | | 0.30 (0.58) | 0.33 (0.78) | 0.045 | 0.24 (0.53) | 0.33 (0.78) | 0.127 |
| Cons_Hospital_Times_impu, mean (SD) | | 0.48 (1.61) | 0.45 (0.99) | 0.024 | 0.47 (1.83) | 0.45 (0.99) | 0.009 |
| Cons_Specialist_Times_impu, mean (SD) | | 0.47 (0.96) | 0.57 (3.10) | 0.044 | 0.51 (1.04) | 0.57 (3.10) | 0.026 |
| Cons_Clinic_Times_impu, mean (SD) | | 0.83 (1.69) | 1.16 (3.49) | 0.121 | 1.07 (1.95) | 1.15 (3.49) | 0.030 |
| Alcohol_frequency_impu, n (%) | |  |  | 0.169 |  |  | 0.240 |
|  | 1 | 6 (9.4) | 79 (10.4) |  | 3 (6.7) | 79 (10.4) |  |
|  | 2 | 15 (23.4) | 160 (21.0) |  | 12 (26.7) | 159 (20.9) |  |
|  | 3 | 26 (40.6) | 285 (37.4) |  | 19 (42.2) | 285 (37.5) |  |
|  | 4 | 10 (15.6) | 166 (21.8) |  | 7 (15.6) | 165 (21.7) |  |
|  | 5 | 7 (10.9) | 73 (9.6) |  | 4 (8.9) | 73 (9.6) |  |
| Alcohol_units_impu, n (%) | |  |  | 0.214 |  |  | 0.260 |
|  | 0 | 7 (10.9) | 81 (10.6) |  | 4 (8.9) | 81 (10.6) |  |
|  | 1 | 28 (43.8) | 371 (48.6) |  | 19 (42.2) | 371 (48.8) |  |
|  | 2 | 25 (39.1) | 233 (30.5) |  | 19 (42.2) | 231 (30.4) |  |
|  | 3 | 4 (6.2) | 78 (10.2) |  | 3 (6.7) | 78 (10.2) |  |
| Alcohol_units_6_impu, n (%) | |  |  | 0.270 |  |  | 0.346 |
|  | 1 | 33 (51.6) | 449 (58.8) |  | 24 (53.3) | 448 (58.9) |  |
|  | 2 | 27 (42.2) | 235 (30.8) |  | 19 (42.2) | 234 (30.7) |  |
|  | 3 | 3 (4.7) | 56 (7.3) |  | 2 (4.4) | 56 (7.4) |  |
|  | 4 | 1 (1.6) | 19 (2.5) |  | 0 (0.0) | 19 (2.5) |  |
|  | 5 | 0 (0.0) | 4 (0.5) |  | 0 (0.0) | 4 (0.5) |  |
| Smoke_impu, n (%) | |  |  | 0.230 |  |  | 0.239 |
|  | 1 | 4 (6.2) | 86 (11.3) |  | 4 (8.9) | 86 (11.3) |  |
|  | 2 | 1 (1.6) | 12 (1.6) |  | 0 (0.0) | 12 (1.6) |  |
|  | 3 | 37 (57.8) | 465 (60.9) |  | 26 (57.8) | 464 (61.0) |  |
|  | 4 | 22 (34.4) | 200 (26.2) |  | 15 (33.3) | 199 (26.1) |  |
| Snuff_chewing_tobacco_impu, n (%) | |  |  | 0.095 |  |  | 0.077 |
|  | 1 | 5 (7.8) | 44 (5.8) |  | 3 (6.7) | 44 (5.8) |  |
|  | 2 | 0 (0.0) | 0 (0.0) |  | 0 (0.0) | 0 (0.0) |  |
|  | 3 | 4 (6.2) | 40 (5.2) |  | 3 (6.7) | 39 (5.1) |  |
|  | 4 | 55 (85.9) | 679 (89.0) |  | 39 (86.7) | 678 (89.1) |  |
| Cod_liver_oil_omega3_impu, n (%) | |  |  | 0.198 |  |  | 0.159 |
|  | 0 | 41 (64.1) | 557 (73.0) |  | 32 (71.1) | 556 (73.1) |  |
|  | 1 | 11 (17.2) | 102 (13.4) |  | 8 (17.8) | 102 (13.4) |  |
|  | 2 | 4 (6.2) | 30 (3.9) |  | 2 (4.4) | 29 (3.8) |  |
|  | 3 | 8 (12.5) | 74 (9.7) |  | 3 (6.7) | 74 (9.7) |  |
| FRUIT_UNITS_T7, mean (SD) | | 1.77 (1.39) | 2.08 (1.95) | 0.187 | 1.89 (1.56) | 2.08 (1.95) | 0.111 |
| RED_MEAT_T7, n (%) | |  |  | 0.411 |  |  | 0.275 |
|  | 1 | 9 (14.1) | 43 (5.6) |  | 4 (8.9) | 42 (5.5) |  |
|  | 2 | 14 (21.9) | 165 (21.6) |  | 13 (28.9) | 165 (21.7) |  |
|  | 3 | 33 (51.6) | 510 (66.8) |  | 26 (57.8) | 509 (66.9) |  |
|  | 4 | 7 (10.9) | 36 (4.7) |  | 1 (2.2) | 36 (4.7) |  |
|  | 5 | 1 (1.6) | 9 (1.2) |  | 1 (2.2) | 9 (1.2) |  |
| FRUITS_VEG_BERRY_T7, n (%) | |  |  | 0.222 |  |  | 0.181 |
|  | 1 | 0 (0.0) | 7 (0.9) |  | 0 (0.0) | 7 (0.9) |  |
|  | 2 | 5 (7.8) | 33 (4.3) |  | 3 (6.7) | 33 (4.3) |  |
|  | 3 | 11 (17.2) | 153 (20.1) |  | 10 (22.2) | 152 (20.0) |  |
|  | 4 | 16 (25.0) | 214 (28.0) |  | 12 (26.7) | 213 (28.0) |  |
|  | 5 | 32 (50.0) | 356 (46.7) |  | 20 (44.4) | 356 (46.8) |  |
| LEAN_FISH_T7, n (%) | |  |  | 0.388 |  |  | 0.296 |
|  | 1 | 7 (10.9) | 20 (2.6) |  | 3 (6.7) | 20 (2.6) |  |
|  | 2 | 9 (14.1) | 100 (13.1) |  | 8 (17.8) | 99 (13.0) |  |
|  | 3 | 42 (65.6) | 534 (70.0) |  | 29 (64.4) | 534 (70.2) |  |
|  | 4 | 6 (9.4) | 98 (12.8) |  | 5 (11.1) | 97 (12.7) |  |
|  | 5 | 0 (0.0) | 11 (1.4) |  | 0 (0.0) | 11 (1.4) |  |
| FAT_FISH_T7, n (%) | |  |  | 0.288 |  |  | 0.304 |
|  | 1 | 9 (14.1) | 88 (11.5) |  | 8 (17.8) | 88 (11.6) |  |
|  | 2 | 26 (40.6) | 260 (34.1) |  | 18 (40.0) | 259 (34.0) |  |
|  | 3 | 26 (40.6) | 369 (48.4) |  | 17 (37.8) | 369 (48.5) |  |
|  | 4 | 1 (1.6) | 37 (4.8) |  | 1 (2.2) | 37 (4.9) |  |
|  | 5 | 2 (3.1) | 9 (1.2) |  | 1 (2.2) | 8 (1.1) |  |
| PHYS_ACTIVITY_LEISURE_T7, n (%) | |  |  | 0.278 |  |  | 0.198 |
|  | 1 | 14 (21.9) | 172 (22.5) |  | 11 (24.4) | 172 (22.6) |  |
|  | 2 | 37 (57.8) | 404 (52.9) |  | 25 (55.6) | 402 (52.8) |  |
|  | 3 | 9 (14.1) | 169 (22.1) |  | 7 (15.6) | 169 (22.2) |  |
|  | 4 | 4 (6.2) | 18 (2.4) |  | 2 (4.4) | 18 (2.4) |  |
| EXERCISE_T7, n (%) | |  |  | 0.217 |  |  | 0.382 |
|  | 1 | 6 (9.4) | 92 (12.1) |  | 2 (4.4) | 92 (12.1) |  |
|  | 2 | 10 (15.6) | 92 (12.1) |  | 9 (20.0) | 91 (12.0) |  |
|  | 3 | 11 (17.2) | 122 (16.0) |  | 6 (13.3) | 122 (16.0) |  |
|  | 4 | 18 (28.1) | 273 (35.8) |  | 14 (31.1) | 273 (35.9) |  |
|  | 5 | 19 (29.7) | 184 (24.1) |  | 14 (31.1) | 183 (24.0) |  |
| **Dataset 5** | | | | | | | |
|  | | **Before matching** | | | **After matching** | | |
|  | | Not anti-hypertensive drug user | Anti-hypertensive drug user | SMD | Not anti-hypertensive drug user | Anti-hypertensive drug user | SMD |
| n | | 64 | 763 |  | 47 | 760 |  |
| AGE, mean (SD) | | 61.95 (11.84) | 69.82 (9.82) | 0.723 | 64.17 (10.77) | 69.86 (9.80) | 0.553 |
| SEX_T7, n (%) | |  |  | 0.013 |  |  | 0.010 |
|  | 1 | 43 (67.2) | 508 (66.6) |  | 31 (66.0) | 505 (66.4) |  |
| BMI, mean (SD) | | 28.99 (5.07) | 29.00 (4.54) | 0.002 | 29.05 (5.18) | 29.00 (4.55) | 0.012 |
| HEART_FAILURE_T7, n (%) | |  |  | 0.263 |  |  | 0.228 |
|  | 0 | 54 (84.4) | 563 (73.8) |  | 39 (83.0) | 561 (73.8) |  |
|  | 1 | 4 (6.2) | 85 (11.1) |  | 3 (6.4) | 84 (11.1) |  |
|  | 2 | 6 (9.4) | 115 (15.1) |  | 5 (10.6) | 115 (15.1) |  |
| ATRIAL_FIBRILLATION_T7, n (%) | |  |  | 0.246 |  |  | 0.227 |
|  | 0 | 39 (60.9) | 544 (71.3) |  | 29 (61.7) | 544 (71.6) |  |
|  | 1 | 12 (18.8) | 126 (16.5) |  | 9 (19.1) | 124 (16.3) |  |
|  | 2 | 13 (20.3) | 93 (12.2) |  | 9 (19.1) | 92 (12.1) |  |
| STROKE_T7, n (%) | |  |  | 0.089 |  |  | 0.067 |
|  | 2 | 9 (14.1) | 132 (17.3) |  | 7 (14.9) | 132 (17.4) |  |
| KIDNEY_DISEASE_T7, n (%) | |  |  | 0.156 |  |  | 0.079 |
|  | 0 | 53 (82.8) | 654 (85.7) |  | 40 (85.1) | 652 (85.8) |  |
|  | 1 | 5 (7.8) | 68 (8.9) |  | 5 (10.6) | 67 (8.8) |  |
|  | 2 | 6 (9.4) | 41 (5.4) |  | 2 (4.3) | 41 (5.4) |  |
| CANCER_T7, n (%) | |  |  | 0.216 |  |  | 0.208 |
|  | 0 | 56 (87.5) | 618 (81.0) |  | 41 (87.2) | 616 (81.1) |  |
|  | 1 | 4 (6.2) | 50 (6.6) |  | 3 (6.4) | 50 (6.6) |  |
|  | 2 | 4 (6.2) | 95 (12.5) |  | 3 (6.4) | 94 (12.4) |  |
| DIABETES_impu_new, n (%) | |  |  | 0.045 |  |  | 0.028 |
|  | 0 | 47 (73.4) | 575 (75.4) |  | 36 (76.6) | 574 (75.5) |  |
|  | 1 | 14 (21.9) | 156 (20.4) |  | 9 (19.1) | 154 (20.3) |  |
|  | 2 | 3 (4.7) | 32 (4.2) |  | 2 (4.3) | 32 (4.2) |  |
| Cons_GP_Times_impu, mean (SD) | | 4.00 (4.38) | 4.99 (6.15) | 0.186 | 4.30 (4.80) | 4.97 (6.13) | 0.123 |
| Cons_Emergency_Times_impu, mean (SD) | | 0.28 (0.58) | 0.33 (0.78) | 0.075 | 0.26 (0.53) | 0.33 (0.78) | 0.118 |
| Cons_Hospital_Times_impu, mean (SD) | | 0.47 (1.61) | 0.46 (0.99) | 0.006 | 0.43 (1.79) | 0.46 (0.99) | 0.024 |
| Cons_Specialist_Times_impu, mean (SD) | | 0.55 (1.01) | 0.53 (2.96) | 0.009 | 0.62 (1.07) | 0.53 (2.97) | 0.040 |
| Cons_Clinic_Times_impu, mean (SD) | | 1.11 (2.39) | 1.20 (3.53) | 0.028 | 1.32 (2.70) | 1.19 (3.54) | 0.040 |
| Alcohol_frequency_impu, n (%) | |  |  | 0.180 |  |  | 0.309 |
|  | 1 | 6 (9.4) | 79 (10.4) |  | 2 (4.3) | 79 (10.4) |  |
|  | 2 | 14 (21.9) | 160 (21.0) |  | 10 (21.3) | 159 (20.9) |  |
|  | 3 | 26 (40.6) | 285 (37.4) |  | 20 (42.6) | 283 (37.2) |  |
|  | 4 | 10 (15.6) | 166 (21.8) |  | 8 (17.0) | 166 (21.8) |  |
|  | 5 | 8 (12.5) | 73 (9.6) |  | 7 (14.9) | 73 (9.6) |  |
| Alcohol_units_impu, n (%) | |  |  | 0.158 |  |  | 0.322 |
|  | 0 | 7 (10.9) | 81 (10.6) |  | 2 (4.3) | 81 (10.7) |  |
|  | 1 | 28 (43.8) | 371 (48.6) |  | 21 (44.7) | 370 (48.7) |  |
|  | 2 | 24 (37.5) | 234 (30.7) |  | 20 (42.6) | 232 (30.5) |  |
|  | 3 | 5 (7.8) | 77 (10.1) |  | 4 (8.5) | 77 (10.1) |  |
| Alcohol_units_6_impu, n (%) | |  |  | 0.266 |  |  | 0.264 |
|  | 1 | 33 (51.6) | 449 (58.8) |  | 23 (48.9) | 448 (58.9) |  |
|  | 2 | 27 (42.2) | 236 (30.9) |  | 20 (42.6) | 234 (30.8) |  |
|  | 3 | 3 (4.7) | 55 (7.2) |  | 3 (6.4) | 55 (7.2) |  |
|  | 4 | 1 (1.6) | 19 (2.5) |  | 1 (2.1) | 19 (2.5) |  |
|  | 5 | 0 (0.0) | 4 (0.5) |  | 0 (0.0) | 4 (0.5) |  |
| Smoke_impu, n (%) | |  |  | 0.230 |  |  | 0.205 |
|  | 1 | 4 (6.2) | 86 (11.3) |  | 4 (8.5) | 86 (11.3) |  |
|  | 2 | 1 (1.6) | 12 (1.6) |  | 0 (0.0) | 12 (1.6) |  |
|  | 3 | 37 (57.8) | 465 (60.9) |  | 30 (63.8) | 463 (60.9) |  |
|  | 4 | 22 (34.4) | 200 (26.2) |  | 13 (27.7) | 199 (26.2) |  |
| Snuff_chewing_tobacco_impu, n (%) | |  |  | 0.093 |  |  | 0.117 |
|  | 1 | 5 (7.8) | 45 (5.9) |  | 4 (8.5) | 45 (5.9) |  |
|  | 2 | 0 (0.0) | 0 (0.0) |  | 0 (0.0) | 0 (0.0) |  |
|  | 3 | 4 (6.2) | 39 (5.1) |  | 3 (6.4) | 39 (5.1) |  |
|  | 4 | 55 (85.9) | 679 (89.0) |  | 40 (85.1) | 676 (88.9) |  |
| Cod_liver_oil_omega3_impu, n (%) | |  |  | 0.193 |  |  | 0.126 |
|  | 0 | 41 (64.1) | 555 (72.7) |  | 33 (70.2) | 553 (72.8) |  |
|  | 1 | 11 (17.2) | 103 (13.5) |  | 7 (14.9) | 102 (13.4) |  |
|  | 2 | 4 (6.2) | 30 (3.9) |  | 3 (6.4) | 30 (3.9) |  |
|  | 3 | 8 (12.5) | 75 (9.8) |  | 4 (8.5) | 75 (9.9) |  |
| FRUIT_UNITS_T7, mean (SD) | | 1.84 (1.38) | 2.10 (2.00) | 0.147 | 2.02 (1.52) | 2.09 (2.00) | 0.041 |
| RED_MEAT_T7, n (%) | |  |  | 0.437 |  |  | 0.200 |
|  | 1 | 10 (15.6) | 44 (5.8) |  | 4 (8.5) | 43 (5.7) |  |
|  | 2 | 14 (21.9) | 166 (21.8) |  | 12 (25.5) | 166 (21.8) |  |
|  | 3 | 32 (50.0) | 506 (66.3) |  | 27 (57.4) | 505 (66.4) |  |
|  | 4 | 7 (10.9) | 38 (5.0) |  | 3 (6.4) | 37 (4.9) |  |
|  | 5 | 1 (1.6) | 9 (1.2) |  | 1 (2.1) | 9 (1.2) |  |
| FRUITS_VEG_BERRY_T7, n (%) | |  |  | 0.222 |  |  | 0.252 |
|  | 1 | 0 (0.0) | 9 (1.2) |  | 0 (0.0) | 9 (1.2) |  |
|  | 2 | 4 (6.2) | 29 (3.8) |  | 4 (8.5) | 29 (3.8) |  |
|  | 3 | 12 (18.8) | 152 (19.9) |  | 9 (19.1) | 151 (19.9) |  |
|  | 4 | 15 (23.4) | 214 (28.0) |  | 12 (25.5) | 214 (28.2) |  |
|  | 5 | 33 (51.6) | 359 (47.1) |  | 22 (46.8) | 357 (47.0) |  |
| LEAN_FISH_T7, n (%) | |  |  | 0.436 |  |  | 0.249 |
|  | 1 | 8 (12.5) | 19 (2.5) |  | 2 (4.3) | 18 (2.4) |  |
|  | 2 | 9 (14.1) | 101 (13.2) |  | 6 (12.8) | 100 (13.2) |  |
|  | 3 | 42 (65.6) | 536 (70.2) |  | 35 (74.5) | 536 (70.5) |  |
|  | 4 | 4 (6.2) | 97 (12.7) |  | 3 (6.4) | 97 (12.8) |  |
|  | 5 | 1 (1.6) | 10 (1.3) |  | 1 (2.1) | 9 (1.2) |  |
| FAT_FISH_T7, n (%) | |  |  | 0.307 |  |  | 0.360 |
|  | 1 | 9 (14.1) | 86 (11.3) |  | 7 (14.9) | 84 (11.1) |  |
|  | 2 | 27 (42.2) | 264 (34.6) |  | 20 (42.6) | 264 (34.7) |  |
|  | 3 | 25 (39.1) | 370 (48.5) |  | 17 (36.2) | 370 (48.7) |  |
|  | 4 | 1 (1.6) | 35 (4.6) |  | 1 (2.1) | 35 (4.6) |  |
|  | 5 | 2 (3.1) | 8 (1.0) |  | 2 (4.3) | 7 (0.9) |  |
| PHYS_ACTIVITY_LEISURE_T7, n (%) | |  |  | 0.287 |  |  | 0.089 |
|  | 1 | 14 (21.9) | 171 (22.4) |  | 11 (23.4) | 170 (22.4) |  |
|  | 2 | 37 (57.8) | 401 (52.6) |  | 26 (55.3) | 400 (52.6) |  |
|  | 3 | 9 (14.1) | 173 (22.7) |  | 9 (19.1) | 172 (22.6) |  |
|  | 4 | 4 (6.2) | 18 (2.4) |  | 1 (2.1) | 18 (2.4) |  |
| EXERCISE_T7, n (%) | |  |  | 0.253 |  |  | 0.319 |
|  | 1 | 5 (7.8) | 90 (11.8) |  | 3 (6.4) | 89 (11.7) |  |
|  | 2 | 11 (17.2) | 91 (11.9) |  | 10 (21.3) | 91 (12.0) |  |
|  | 3 | 12 (18.8) | 122 (16.0) |  | 6 (12.8) | 122 (16.1) |  |
|  | 4 | 18 (28.1) | 273 (35.8) |  | 15 (31.9) | 272 (35.8) |  |
|  | 5 | 18 (28.1) | 187 (24.5) |  | 13 (27.7) | 186 (24.5) |  |
| **Dataset 6** | | | | | | | |
|  | | **Before matching** | | | **After matching** | | |
|  | | Not anti-hypertensive drug user | Anti-hypertensive drug user | SMD | Not anti-hypertensive drug user | Anti-hypertensive drug user | SMD |
| n | | 64 | 763 |  | 49 | 759 |  |
| AGE, mean (SD) | | 61.95 (11.84) | 69.82 (9.82) | 0.723 | 65.08 (10.80) | 69.88 (9.81) | 0.465 |
| SEX_T7, n (%) | |  |  | 0.013 |  |  | 0.058 |
|  | 1 | 43 (67.2) | 508 (66.6) |  | 34 (69.4) | 506 (66.7) |  |
| BMI, mean (SD) | | 28.99 (5.07) | 29.00 (4.54) | 0.002 | 28.60 (4.62) | 29.00 (4.54) | 0.087 |
| HEART_FAILURE_T7, n (%) | |  |  | 0.353 |  |  | 0.291 |
|  | 0 | 55 (85.9) | 557 (73.0) |  | 41 (83.7) | 553 (72.9) |  |
|  | 1 | 2 (3.1) | 78 (10.2) |  | 2 (4.1) | 78 (10.3) |  |
|  | 2 | 7 (10.9) | 128 (16.8) |  | 6 (12.2) | 128 (16.9) |  |
| ATRIAL_FIBRILLATION_T7, n (%) | |  |  | 0.222 |  |  | 0.184 |
|  | 0 | 40 (62.5) | 544 (71.3) |  | 31 (63.3) | 541 (71.3) |  |
|  | 1 | 11 (17.2) | 123 (16.1) |  | 9 (18.4) | 122 (16.1) |  |
|  | 2 | 13 (20.3) | 96 (12.6) |  | 9 (18.4) | 96 (12.6) |  |
| STROKE_T7, n (%) | |  |  | 0.089 |  |  | 0.028 |
|  | 2 | 9 (14.1) | 132 (17.3) |  | 8 (16.3) | 132 (17.4) |  |
| KIDNEY_DISEASE_T7, n (%) | |  |  | 0.186 |  |  | 0.063 |
|  | 0 | 53 (82.8) | 651 (85.3) |  | 41 (83.7) | 648 (85.4) |  |
|  | 1 | 5 (7.8) | 75 (9.8) |  | 5 (10.2) | 75 (9.9) |  |
|  | 2 | 6 (9.4) | 37 (4.8) |  | 3 (6.1) | 36 (4.7) |  |
| CANCER_T7, n (%) | |  |  | 0.273 |  |  | 0.320 |
|  | 0 | 56 (87.5) | 619 (81.1) |  | 42 (85.7) | 616 (81.2) |  |
|  | 1 | 5 (7.8) | 51 (6.7) |  | 5 (10.2) | 50 (6.6) |  |
|  | 2 | 3 (4.7) | 93 (12.2) |  | 2 (4.1) | 93 (12.3) |  |
| DIABETES_impu_new, n (%) | |  |  | 0.069 |  |  | 0.065 |
|  | 0 | 47 (73.4) | 575 (75.4) |  | 37 (75.5) | 571 (75.2) |  |
|  | 1 | 14 (21.9) | 147 (19.3) |  | 10 (20.4) | 147 (19.4) |  |
|  | 2 | 3 (4.7) | 41 (5.4) |  | 2 (4.1) | 41 (5.4) |  |
| Cons_GP_Times_impu, mean (SD) | | 3.34 (2.84) | 4.90 (5.80) | 0.340 | 3.69 (2.99) | 4.90 (5.82) | 0.261 |
| Cons_Emergency_Times_impu, mean (SD) | | 0.28 (0.55) | 0.33 (0.78) | 0.065 | 0.24 (0.48) | 0.33 (0.78) | 0.125 |
| Cons_Hospital_Times_impu, mean (SD) | | 0.48 (1.61) | 0.46 (1.00) | 0.015 | 0.47 (1.76) | 0.47 (1.00) | 0.003 |
| Cons_Specialist_Times_impu, mean (SD) | | 0.53 (1.01) | 0.64 (3.24) | 0.045 | 0.61 (1.06) | 0.64 (3.25) | 0.011 |
| Cons_Clinic_Times_impu, mean (SD) | | 0.69 (1.23) | 1.16 (3.50) | 0.181 | 0.80 (1.35) | 1.16 (3.51) | 0.138 |
| Alcohol_frequency_impu, n (%) | |  |  | 0.169 |  |  | 0.227 |
|  | 1 | 6 (9.4) | 79 (10.4) |  | 3 (6.1) | 79 (10.4) |  |
|  | 2 | 15 (23.4) | 160 (21.0) |  | 11 (22.4) | 160 (21.1) |  |
|  | 3 | 26 (40.6) | 285 (37.4) |  | 21 (42.9) | 284 (37.4) |  |
|  | 4 | 10 (15.6) | 166 (21.8) |  | 8 (16.3) | 163 (21.5) |  |
|  | 5 | 7 (10.9) | 73 (9.6) |  | 6 (12.2) | 73 (9.6) |  |
| Alcohol_units_impu, n (%) | |  |  | 0.187 |  |  | 0.281 |
|  | 0 | 7 (10.9) | 82 (10.7) |  | 3 (6.1) | 82 (10.8) |  |
|  | 1 | 29 (45.3) | 371 (48.6) |  | 23 (46.9) | 370 (48.7) |  |
|  | 2 | 24 (37.5) | 233 (30.5) |  | 20 (40.8) | 230 (30.3) |  |
|  | 3 | 4 (6.2) | 77 (10.1) |  | 3 (6.1) | 77 (10.1) |  |
| Alcohol_units_6_impu, n (%) | |  |  | 0.302 |  |  | 0.287 |
|  | 1 | 34 (53.1) | 448 (58.7) |  | 25 (51.0) | 446 (58.8) |  |
|  | 2 | 27 (42.2) | 236 (30.9) |  | 21 (42.9) | 234 (30.8) |  |
|  | 3 | 2 (3.1) | 56 (7.3) |  | 2 (4.1) | 56 (7.4) |  |
|  | 4 | 1 (1.6) | 19 (2.5) |  | 1 (2.0) | 19 (2.5) |  |
|  | 5 | 0 (0.0) | 4 (0.5) |  | 0 (0.0) | 4 (0.5) |  |
| Smoke_impu, n (%) | |  |  | 0.230 |  |  | 0.223 |
|  | 1 | 4 (6.2) | 86 (11.3) |  | 4 (8.2) | 86 (11.3) |  |
|  | 2 | 1 (1.6) | 12 (1.6) |  | 0 (0.0) | 12 (1.6) |  |
|  | 3 | 37 (57.8) | 465 (60.9) |  | 30 (61.2) | 463 (61.0) |  |
|  | 4 | 22 (34.4) | 200 (26.2) |  | 15 (30.6) | 198 (26.1) |  |
| Snuff_chewing_tobacco_impu, n (%) | |  |  | 0.092 |  |  | 0.038 |
|  | 1 | 5 (7.8) | 44 (5.8) |  | 3 (6.1) | 43 (5.7) |  |
|  | 2 | 0 (0.0) | 0 (0.0) |  | 0 (0.0) | 0 (0.0) |  |
|  | 3 | 4 (6.2) | 41 (5.4) |  | 3 (6.1) | 41 (5.4) |  |
|  | 4 | 55 (85.9) | 678 (88.9) |  | 43 (87.8) | 675 (88.9) |  |
| Cod_liver_oil_omega3_impu, n (%) | |  |  | 0.193 |  |  | 0.148 |
|  | 0 | 41 (64.1) | 556 (72.9) |  | 33 (67.3) | 554 (73.0) |  |
|  | 1 | 11 (17.2) | 101 (13.2) |  | 9 (18.4) | 100 (13.2) |  |
|  | 2 | 4 (6.2) | 32 (4.2) |  | 2 (4.1) | 31 (4.1) |  |
|  | 3 | 8 (12.5) | 74 (9.7) |  | 5 (10.2) | 74 (9.7) |  |
| FRUIT_UNITS_T7, mean (SD) | | 1.81 (1.37) | 2.06 (1.94) | 0.148 | 1.92 (1.48) | 2.06 (1.94) | 0.081 |
| RED_MEAT_T7, n (%) | |  |  | 0.503 |  |  | 0.377 |
|  | 1 | 11 (17.2) | 43 (5.6) |  | 7 (14.3) | 41 (5.4) |  |
|  | 2 | 14 (21.9) | 163 (21.4) |  | 12 (24.5) | 163 (21.5) |  |
|  | 3 | 32 (50.0) | 508 (66.6) |  | 27 (55.1) | 506 (66.7) |  |
|  | 4 | 7 (10.9) | 38 (5.0) |  | 3 (6.1) | 38 (5.0) |  |
|  | 5 | 0 (0.0) | 11 (1.4) |  | 0 (0.0) | 11 (1.4) |  |
| FRUITS_VEG_BERRY_T7, n (%) | |  |  | 0.187 |  |  | 0.171 |
|  | 1 | 0 (0.0) | 7 (0.9) |  | 0 (0.0) | 7 (0.9) |  |
|  | 2 | 4 (6.2) | 30 (3.9) |  | 3 (6.1) | 30 (4.0) |  |
|  | 3 | 12 (18.8) | 152 (19.9) |  | 10 (20.4) | 151 (19.9) |  |
|  | 4 | 16 (25.0) | 214 (28.0) |  | 14 (28.6) | 213 (28.1) |  |
|  | 5 | 32 (50.0) | 360 (47.2) |  | 22 (44.9) | 358 (47.2) |  |
| LEAN_FISH_T7, n (%) | |  |  | 0.438 |  |  | 0.287 |
|  | 1 | 8 (12.5) | 20 (2.6) |  | 3 (6.1) | 19 (2.5) |  |
|  | 2 | 8 (12.5) | 100 (13.1) |  | 6 (12.2) | 100 (13.2) |  |
|  | 3 | 43 (67.2) | 535 (70.1) |  | 36 (73.5) | 533 (70.2) |  |
|  | 4 | 5 (7.8) | 97 (12.7) |  | 4 (8.2) | 96 (12.6) |  |
|  | 5 | 0 (0.0) | 11 (1.4) |  | 0 (0.0) | 11 (1.4) |  |
| FAT_FISH_T7, n (%) | |  |  | 0.336 |  |  | 0.344 |
|  | 1 | 8 (12.5) | 84 (11.0) |  | 7 (14.3) | 83 (10.9) |  |
|  | 2 | 29 (45.3) | 260 (34.1) |  | 21 (42.9) | 259 (34.1) |  |
|  | 3 | 24 (37.5) | 370 (48.5) |  | 18 (36.7) | 369 (48.6) |  |
|  | 4 | 1 (1.6) | 38 (5.0) |  | 1 (2.0) | 38 (5.0) |  |
|  | 5 | 2 (3.1) | 11 (1.4) |  | 2 (4.1) | 10 (1.3) |  |
| PHYS_ACTIVITY_LEISURE_T7, n (%) | |  |  | 0.275 |  |  | 0.131 |
|  | 1 | 14 (21.9) | 169 (22.1) |  | 11 (22.4) | 168 (22.1) |  |
|  | 2 | 37 (57.8) | 399 (52.3) |  | 27 (55.1) | 398 (52.4) |  |
|  | 3 | 9 (14.1) | 174 (22.8) |  | 9 (18.4) | 173 (22.8) |  |
|  | 4 | 4 (6.2) | 21 (2.8) |  | 2 (4.1) | 20 (2.6) |  |
| EXERCISE_T7, n (%) | |  |  | 0.219 |  |  | 0.307 |
|  | 1 | 6 (9.4) | 91 (11.9) |  | 3 (6.1) | 91 (12.0) |  |
|  | 2 | 10 (15.6) | 90 (11.8) |  | 10 (20.4) | 89 (11.7) |  |
|  | 3 | 12 (18.8) | 120 (15.7) |  | 7 (14.3) | 120 (15.8) |  |
|  | 4 | 18 (28.1) | 276 (36.2) |  | 16 (32.7) | 275 (36.2) |  |
|  | 5 | 18 (28.1) | 186 (24.4) |  | 13 (26.5) | 184 (24.2) |  |
| **Dataset 7** | | | | | | | |
|  | | **Before matching** | | | **After matching** | | |
|  | | Not anti-hypertensive drug user | Anti-hypertensive drug user | SMD | Not anti-hypertensive drug user | Anti-hypertensive drug user | SMD |
| n | | 64 | 763 |  | 47 | 760 |  |
| AGE, mean (SD) | | 61.95 (11.84) | 69.82 (9.82) | 0.723 | 64.21 (11.06) | 69.85 (9.81) | 0.539 |
| SEX_T7, n (%) | |  |  | 0.013 |  |  | 0.013 |
|  | 1 | 43 (67.2) | 508 (66.6) |  | 31 (66.0) | 506 (66.6) |  |
| BMI, mean (SD) | | 28.99 (5.07) | 28.99 (4.54) | 0.001 | 28.64 (4.58) | 28.99 (4.54) | 0.077 |
| HEART_FAILURE_T7, n (%) | |  |  | 0.313 |  |  | 0.259 |
|  | 0 | 54 (84.4) | 564 (73.9) |  | 39 (83.0) | 561 (73.8) |  |
|  | 1 | 2 (3.1) | 77 (10.1) |  | 2 (4.3) | 77 (10.1) |  |
|  | 2 | 8 (12.5) | 122 (16.0) |  | 6 (12.8) | 122 (16.1) |  |
| ATRIAL_FIBRILLATION_T7, n (%) | |  |  | 0.236 |  |  | 0.179 |
|  | 0 | 39 (60.9) | 544 (71.3) |  | 31 (66.0) | 543 (71.4) |  |
|  | 1 | 12 (18.8) | 121 (15.9) |  | 7 (14.9) | 121 (15.9) |  |
|  | 2 | 13 (20.3) | 98 (12.8) |  | 9 (19.1) | 96 (12.6) |  |
| STROKE_T7, n (%) | |  |  | 0.187 |  |  | 0.129 |
|  | 2 | 7 (10.9) | 133 (17.4) |  | 6 (12.8) | 132 (17.4) |  |
| KIDNEY_DISEASE_T7, n (%) | |  |  | 0.151 |  |  | 0.062 |
|  | 0 | 53 (82.8) | 652 (85.5) |  | 41 (87.2) | 650 (85.5) |  |
|  | 1 | 5 (7.8) | 69 (9.0) |  | 4 (8.5) | 68 (8.9) |  |
|  | 2 | 6 (9.4) | 42 (5.5) |  | 2 (4.3) | 42 (5.5) |  |
| CANCER_T7, n (%) | |  |  | 0.179 |  |  | 0.222 |
|  | 0 | 56 (87.5) | 620 (81.3) |  | 41 (87.2) | 617 (81.2) |  |
|  | 1 | 3 (4.7) | 45 (5.9) |  | 3 (6.4) | 45 (5.9) |  |
|  | 2 | 5 (7.8) | 98 (12.8) |  | 3 (6.4) | 98 (12.9) |  |
| DIABETES_impu_new, n (%) | |  |  | 0.051 |  |  | 0.079 |
|  | 0 | 47 (73.4) | 575 (75.4) |  | 37 (78.7) | 573 (75.4) |  |
|  | 1 | 14 (21.9) | 151 (19.8) |  | 8 (17.0) | 150 (19.7) |  |
|  | 2 | 3 (4.7) | 37 (4.8) |  | 2 (4.3) | 37 (4.9) |  |
| Cons_GP_Times_impu, mean (SD) | | 4.00 (4.50) | 4.92 (5.50) | 0.182 | 4.43 (4.97) | 4.92 (5.51) | 0.094 |
| Cons_Emergency_Times_impu, mean (SD) | | 0.36 (0.70) | 0.34 (0.78) | 0.025 | 0.32 (0.66) | 0.34 (0.79) | 0.032 |
| Cons_Hospital_Times_impu, mean (SD) | | 0.48 (1.61) | 0.47 (1.01) | 0.014 | 0.45 (1.78) | 0.47 (1.01) | 0.014 |
| Cons_Specialist_Times_impu, mean (SD) | | 0.56 (1.18) | 0.54 (2.96) | 0.010 | 0.64 (1.29) | 0.54 (2.96) | 0.043 |
| Cons_Clinic_Times_impu, mean (SD) | | 0.83 (1.53) | 1.19 (3.56) | 0.132 | 1.02 (1.71) | 1.19 (3.57) | 0.061 |
| Alcohol_frequency_impu, n (%) | |  |  | 0.171 |  |  | 0.232 |
|  | 1 | 6 (9.4) | 79 (10.4) |  | 3 (6.4) | 79 (10.4) |  |
|  | 2 | 14 (21.9) | 160 (21.0) |  | 8 (17.0) | 159 (20.9) |  |
|  | 3 | 27 (42.2) | 285 (37.4) |  | 22 (46.8) | 284 (37.4) |  |
|  | 4 | 10 (15.6) | 166 (21.8) |  | 9 (19.1) | 166 (21.8) |  |
|  | 5 | 7 (10.9) | 73 (9.6) |  | 5 (10.6) | 72 (9.5) |  |
| Alcohol_units_impu, n (%) | |  |  | 0.160 |  |  | 0.244 |
|  | 0 | 7 (10.9) | 81 (10.6) |  | 3 (6.4) | 81 (10.7) |  |
|  | 1 | 28 (43.8) | 372 (48.8) |  | 20 (42.6) | 371 (48.8) |  |
|  | 2 | 24 (37.5) | 233 (30.5) |  | 19 (40.4) | 231 (30.4) |  |
|  | 3 | 5 (7.8) | 77 (10.1) |  | 5 (10.6) | 77 (10.1) |  |
| Alcohol_units_6_impu, n (%) | |  |  | 0.274 |  |  | 0.270 |
|  | 1 | 33 (51.6) | 448 (58.7) |  | 27 (57.4) | 447 (58.8) |  |
|  | 2 | 27 (42.2) | 235 (30.8) |  | 17 (36.2) | 233 (30.7) |  |
|  | 3 | 3 (4.7) | 57 (7.5) |  | 3 (6.4) | 57 (7.5) |  |
|  | 4 | 1 (1.6) | 18 (2.4) |  | 0 (0.0) | 18 (2.4) |  |
|  | 5 | 0 (0.0) | 5 (0.7) |  | 0 (0.0) | 5 (0.7) |  |
| Smoke_impu, n (%) | |  |  | 0.230 |  |  | 0.249 |
|  | 1 | 4 (6.2) | 86 (11.3) |  | 4 (8.5) | 86 (11.3) |  |
|  | 2 | 1 (1.6) | 12 (1.6) |  | 0 (0.0) | 12 (1.6) |  |
|  | 3 | 37 (57.8) | 465 (60.9) |  | 27 (57.4) | 462 (60.8) |  |
|  | 4 | 22 (34.4) | 200 (26.2) |  | 16 (34.0) | 200 (26.3) |  |
| Snuff_chewing_tobacco_impu, n (%) | |  |  | 0.098 |  |  | 0.086 |
|  | 1 | 5 (7.8) | 44 (5.8) |  | 2 (4.3) | 44 (5.8) |  |
|  | 2 | 0 (0.0) | 0 (0.0) |  | 0 (0.0) | 0 (0.0) |  |
|  | 3 | 4 (6.2) | 39 (5.1) |  | 3 (6.4) | 39 (5.1) |  |
|  | 4 | 55 (85.9) | 680 (89.1) |  | 42 (89.4) | 677 (89.1) |  |
| Cod_liver_oil_omega3_impu, n (%) | |  |  | 0.210 |  |  | 0.175 |
|  | 0 | 41 (64.1) | 557 (73.0) |  | 31 (66.0) | 555 (73.0) |  |
|  | 1 | 12 (18.8) | 101 (13.2) |  | 9 (19.1) | 100 (13.2) |  |
|  | 2 | 4 (6.2) | 29 (3.8) |  | 2 (4.3) | 29 (3.8) |  |
|  | 3 | 7 (10.9) | 76 (10.0) |  | 5 (10.6) | 76 (10.0) |  |
| FRUIT_UNITS_T7, mean (SD) | | 1.80 (1.42) | 2.07 (1.95) | 0.161 | 1.98 (1.52) | 2.06 (1.95) | 0.049 |
| RED_MEAT_T7, n (%) | |  |  | 0.443 |  |  | 0.345 |
|  | 1 | 10 (15.6) | 43 (5.6) |  | 7 (14.9) | 42 (5.5) |  |
|  | 2 | 14 (21.9) | 165 (21.6) |  | 11 (23.4) | 164 (21.6) |  |
|  | 3 | 32 (50.0) | 508 (66.6) |  | 26 (55.3) | 507 (66.7) |  |
|  | 4 | 7 (10.9) | 38 (5.0) |  | 2 (4.3) | 38 (5.0) |  |
|  | 5 | 1 (1.6) | 9 (1.2) |  | 1 (2.1) | 9 (1.2) |  |
| FRUITS_VEG_BERRY_T7, n (%) | |  |  | 0.233 |  |  | 0.266 |
|  | 1 | 0 (0.0) | 8 (1.0) |  | 0 (0.0) | 8 (1.1) |  |
|  | 2 | 4 (6.2) | 31 (4.1) |  | 2 (4.3) | 30 (3.9) |  |
|  | 3 | 12 (18.8) | 153 (20.1) |  | 10 (21.3) | 153 (20.1) |  |
|  | 4 | 14 (21.9) | 213 (27.9) |  | 9 (19.1) | 213 (28.0) |  |
|  | 5 | 34 (53.1) | 358 (46.9) |  | 26 (55.3) | 356 (46.8) |  |
| LEAN_FISH_T7, n (%) | |  |  | 0.434 |  |  | 0.353 |
|  | 1 | 8 (12.5) | 19 (2.5) |  | 3 (6.4) | 16 (2.1) |  |
|  | 2 | 9 (14.1) | 100 (13.1) |  | 9 (19.1) | 100 (13.2) |  |
|  | 3 | 42 (65.6) | 534 (70.0) |  | 31 (66.0) | 534 (70.3) |  |
|  | 4 | 4 (6.2) | 96 (12.6) |  | 4 (8.5) | 96 (12.6) |  |
|  | 5 | 1 (1.6) | 14 (1.8) |  | 0 (0.0) | 14 (1.8) |  |
| FAT_FISH_T7, n (%) | |  |  | 0.328 |  |  | 0.249 |
|  | 1 | 8 (12.5) | 85 (11.1) |  | 7 (14.9) | 84 (11.1) |  |
|  | 2 | 27 (42.2) | 260 (34.1) |  | 19 (40.4) | 259 (34.1) |  |
|  | 3 | 25 (39.1) | 368 (48.2) |  | 19 (40.4) | 367 (48.3) |  |
|  | 4 | 1 (1.6) | 38 (5.0) |  | 1 (2.1) | 38 (5.0) |  |
|  | 5 | 3 (4.7) | 12 (1.6) |  | 1 (2.1) | 12 (1.6) |  |
| PHYS_ACTIVITY_LEISURE_T7, n (%) | |  |  | 0.279 |  |  | 0.168 |
|  | 1 | 14 (21.9) | 173 (22.7) |  | 10 (21.3) | 172 (22.6) |  |
|  | 2 | 37 (57.8) | 400 (52.4) |  | 27 (57.4) | 400 (52.6) |  |
|  | 3 | 9 (14.1) | 171 (22.4) |  | 8 (17.0) | 169 (22.2) |  |
|  | 4 | 4 (6.2) | 19 (2.5) |  | 2 (4.3) | 19 (2.5) |  |
| EXERCISE_T7, n (%) | |  |  | 0.234 |  |  | 0.312 |
|  | 1 | 6 (9.4) | 93 (12.2) |  | 3 (6.4) | 92 (12.1) |  |
|  | 2 | 11 (17.2) | 90 (11.8) |  | 9 (19.1) | 90 (11.8) |  |
|  | 3 | 11 (17.2) | 120 (15.7) |  | 7 (14.9) | 120 (15.8) |  |
|  | 4 | 18 (28.1) | 275 (36.0) |  | 14 (29.8) | 275 (36.2) |  |
|  | 5 | 18 (28.1) | 185 (24.2) |  | 14 (29.8) | 183 (24.1) |  |
| **Dataset 8** | | | | | | | |
|  | | **Before matching** | | | **After matching** | | |
|  | | Not anti-hypertensive drug user | Anti-hypertensive drug user | SMD | Not anti-hypertensive drug user | Anti-hypertensive drug user | SMD |
| n | | 64 | 763 |  | 47 | 762 |  |
| AGE, mean (SD) | | 61.95 (11.84) | 69.82 (9.82) | 0.723 | 65.79 (10.75) | 69.86 (9.77) | 0.396 |
| SEX_T7, n (%) | |  |  | 0.013 |  |  | 0.030 |
|  | 1 | 43 (67.2) | 508 (66.6) |  | 32 (68.1) | 508 (66.7) |  |
| BMI, mean (SD) | | 28.99 (5.07) | 29.00 (4.54) | 0.001 | 28.67 (4.79) | 29.00 (4.54) | 0.069 |
| HEART_FAILURE_T7, n (%) | |  |  | 0.379 |  |  | 0.334 |
|  | 0 | 54 (84.4) | 565 (74.0) |  | 38 (80.9) | 564 (74.0) |  |
|  | 1 | 1 (1.6) | 76 (10.0) |  | 1 (2.1) | 76 (10.0) |  |
|  | 2 | 9 (14.1) | 122 (16.0) |  | 8 (17.0) | 122 (16.0) |  |
| ATRIAL_FIBRILLATION_T7, n (%) | |  |  | 0.215 |  |  | 0.165 |
|  | 0 | 40 (62.5) | 545 (71.4) |  | 30 (63.8) | 545 (71.5) |  |
|  | 1 | 12 (18.8) | 128 (16.8) |  | 10 (21.3) | 128 (16.8) |  |
|  | 2 | 12 (18.8) | 90 (11.8) |  | 7 (14.9) | 89 (11.7) |  |
| STROKE_T7, n (%) | |  |  | 0.128 |  |  | 0.059 |
|  | 2 | 8 (12.5) | 130 (17.0) |  | 7 (14.9) | 130 (17.1) |  |
| KIDNEY_DISEASE_T7, n (%) | |  |  | 0.209 |  |  | 0.112 |
|  | 0 | 53 (82.8) | 653 (85.6) |  | 39 (83.0) | 652 (85.6) |  |
|  | 1 | 4 (6.2) | 67 (8.8) |  | 4 (8.5) | 67 (8.8) |  |
|  | 2 | 7 (10.9) | 43 (5.6) |  | 4 (8.5) | 43 (5.6) |  |
| CANCER_T7, n (%) | |  |  | 0.202 |  |  | 0.199 |
|  | 0 | 56 (87.5) | 620 (81.3) |  | 40 (85.1) | 619 (81.2) |  |
|  | 1 | 4 (6.2) | 52 (6.8) |  | 4 (8.5) | 52 (6.8) |  |
|  | 2 | 4 (6.2) | 91 (11.9) |  | 3 (6.4) | 91 (11.9) |  |
| DIABETES_impu_new, n (%) | |  |  | 0.089 |  |  | 0.041 |
|  | 0 | 46 (71.9) | 576 (75.5) |  | 35 (74.5) | 575 (75.5) |  |
|  | 1 | 15 (23.4) | 151 (19.8) |  | 10 (21.3) | 151 (19.8) |  |
|  | 2 | 3 (4.7) | 36 (4.7) |  | 2 (4.3) | 36 (4.7) |  |
| Cons_GP_Times_impu, mean (SD) | | 4.22 (4.68) | 5.06 (6.40) | 0.149 | 4.79 (5.26) | 5.05 (6.40) | 0.045 |
| Cons_Emergency_Times_impu, mean (SD) | | 0.28 (0.55) | 0.34 (0.80) | 0.085 | 0.23 (0.48) | 0.34 (0.80) | 0.161 |
| Cons_Hospital_Times_impu, mean (SD) | | 0.47 (1.61) | 0.45 (0.98) | 0.017 | 0.47 (1.79) | 0.45 (0.98) | 0.015 |
| Cons_Specialist_Times_impu, mean (SD) | | 0.66 (1.73) | 0.58 (3.05) | 0.031 | 0.72 (1.95) | 0.58 (3.05) | 0.056 |
| Cons_Clinic_Times_impu, mean (SD) | | 0.95 (2.18) | 1.32 (4.56) | 0.103 | 1.21 (2.48) | 1.32 (4.57) | 0.029 |
| Alcohol_frequency_impu, n (%) | |  |  | 0.160 |  |  | 0.254 |
|  | 1 | 7 (10.9) | 79 (10.4) |  | 3 (6.4) | 79 (10.4) |  |
|  | 2 | 14 (21.9) | 160 (21.0) |  | 11 (23.4) | 159 (20.9) |  |
|  | 3 | 26 (40.6) | 285 (37.4) |  | 20 (42.6) | 285 (37.4) |  |
|  | 4 | 10 (15.6) | 166 (21.8) |  | 7 (14.9) | 166 (21.8) |  |
|  | 5 | 7 (10.9) | 73 (9.6) |  | 6 (12.8) | 73 (9.6) |  |
| Alcohol_units_impu, n (%) | |  |  | 0.205 |  |  | 0.234 |
|  | 0 | 8 (12.5) | 81 (10.6) |  | 4 (8.5) | 81 (10.6) |  |
|  | 1 | 28 (43.8) | 371 (48.6) |  | 21 (44.7) | 371 (48.7) |  |
|  | 2 | 24 (37.5) | 233 (30.5) |  | 19 (40.4) | 232 (30.4) |  |
|  | 3 | 4 (6.2) | 78 (10.2) |  | 3 (6.4) | 78 (10.2) |  |
| Alcohol_units_6_impu, n (%) | |  |  | 0.300 |  |  | 0.314 |
|  | 1 | 34 (53.1) | 448 (58.7) |  | 23 (48.9) | 448 (58.8) |  |
|  | 2 | 27 (42.2) | 237 (31.1) |  | 21 (44.7) | 236 (31.0) |  |
|  | 3 | 2 (3.1) | 55 (7.2) |  | 2 (4.3) | 55 (7.2) |  |
|  | 4 | 1 (1.6) | 18 (2.4) |  | 1 (2.1) | 18 (2.4) |  |
|  | 5 | 0 (0.0) | 5 (0.7) |  | 0 (0.0) | 5 (0.7) |  |
| Smoke_impu, n (%) | |  |  | 0.230 |  |  | 0.228 |
|  | 1 | 4 (6.2) | 86 (11.3) |  | 4 (8.5) | 86 (11.3) |  |
|  | 2 | 1 (1.6) | 12 (1.6) |  | 0 (0.0) | 12 (1.6) |  |
|  | 3 | 37 (57.8) | 465 (60.9) |  | 28 (59.6) | 465 (61.0) |  |
|  | 4 | 22 (34.4) | 200 (26.2) |  | 15 (31.9) | 199 (26.1) |  |
| Snuff_chewing_tobacco_impu, n (%) | |  |  | 0.095 |  |  | 0.052 |
|  | 1 | 5 (7.8) | 44 (5.8) |  | 3 (6.4) | 44 (5.8) |  |
|  | 2 | 0 (0.0) | 0 (0.0) |  | 0 (0.0) | 0 (0.0) |  |
|  | 3 | 4 (6.2) | 40 (5.2) |  | 2 (4.3) | 40 (5.2) |  |
|  | 4 | 55 (85.9) | 679 (89.0) |  | 42 (89.4) | 678 (89.0) |  |
| Cod_liver_oil_omega3_impu, n (%) | |  |  | 0.205 |  |  | 0.189 |
|  | 0 | 41 (64.1) | 558 (73.1) |  | 32 (68.1) | 557 (73.1) |  |
|  | 1 | 11 (17.2) | 99 (13.0) |  | 9 (19.1) | 99 (13.0) |  |
|  | 2 | 4 (6.2) | 28 (3.7) |  | 1 (2.1) | 28 (3.7) |  |
|  | 3 | 8 (12.5) | 78 (10.2) |  | 5 (10.6) | 78 (10.2) |  |
| FRUIT_UNITS_T7, mean (SD) | | 1.73 (1.41) | 2.07 (1.96) | 0.200 | 1.81 (1.54) | 2.08 (1.96) | 0.152 |
| RED_MEAT_T7, n (%) | |  |  | 0.461 |  |  | 0.346 |
|  | 1 | 9 (14.1) | 45 (5.9) |  | 5 (10.6) | 45 (5.9) |  |
|  | 2 | 15 (23.4) | 162 (21.2) |  | 13 (27.7) | 162 (21.3) |  |
|  | 3 | 32 (50.0) | 508 (66.6) |  | 25 (53.2) | 508 (66.7) |  |
|  | 4 | 8 (12.5) | 39 (5.1) |  | 4 (8.5) | 38 (5.0) |  |
|  | 5 | 0 (0.0) | 9 (1.2) |  | 0 (0.0) | 9 (1.2) |  |
| FRUITS_VEG_BERRY_T7, n (%) | |  |  | 0.197 |  |  | 0.199 |
|  | 1 | 0 (0.0) | 7 (0.9) |  | 0 (0.0) | 7 (0.9) |  |
|  | 2 | 4 (6.2) | 31 (4.1) |  | 3 (6.4) | 31 (4.1) |  |
|  | 3 | 13 (20.3) | 151 (19.8) |  | 11 (23.4) | 151 (19.8) |  |
|  | 4 | 15 (23.4) | 215 (28.2) |  | 13 (27.7) | 214 (28.1) |  |
|  | 5 | 32 (50.0) | 359 (47.1) |  | 20 (42.6) | 359 (47.1) |  |
| LEAN_FISH_T7, n (%) | |  |  | 0.378 |  |  | 0.193 |
|  | 1 | 7 (10.9) | 20 (2.6) |  | 3 (6.4) | 20 (2.6) |  |
|  | 2 | 8 (12.5) | 102 (13.4) |  | 6 (12.8) | 101 (13.3) |  |
|  | 3 | 42 (65.6) | 533 (69.9) |  | 32 (68.1) | 533 (69.9) |  |
|  | 4 | 5 (7.8) | 96 (12.6) |  | 5 (10.6) | 96 (12.6) |  |
|  | 5 | 2 (3.1) | 12 (1.6) |  | 1 (2.1) | 12 (1.6) |  |
| FAT_FISH_T7, n (%) | |  |  | 0.335 |  |  | 0.353 |
|  | 1 | 9 (14.1) | 88 (11.5) |  | 8 (17.0) | 87 (11.4) |  |
|  | 2 | 26 (40.6) | 260 (34.1) |  | 19 (40.4) | 260 (34.1) |  |
|  | 3 | 25 (39.1) | 367 (48.1) |  | 17 (36.2) | 367 (48.2) |  |
|  | 4 | 1 (1.6) | 38 (5.0) |  | 1 (2.1) | 38 (5.0) |  |
|  | 5 | 3 (4.7) | 10 (1.3) |  | 2 (4.3) | 10 (1.3) |  |
| PHYS_ACTIVITY_LEISURE_T7, n (%) | |  |  | 0.326 |  |  | 0.203 |
|  | 1 | 14 (21.9) | 174 (22.8) |  | 9 (19.1) | 174 (22.8) |  |
|  | 2 | 37 (57.8) | 402 (52.7) |  | 29 (61.7) | 402 (52.8) |  |
|  | 3 | 9 (14.1) | 175 (22.9) |  | 8 (17.0) | 175 (23.0) |  |
|  | 4 | 4 (6.2) | 12 (1.6) |  | 1 (2.1) | 11 (1.4) |  |
| EXERCISE_T7, n (%) | |  |  | 0.260 |  |  | 0.427 |
|  | 1 | 5 (7.8) | 93 (12.2) |  | 2 (4.3) | 93 (12.2) |  |
|  | 2 | 10 (15.6) | 89 (11.7) |  | 10 (21.3) | 89 (11.7) |  |
|  | 3 | 13 (20.3) | 119 (15.6) |  | 5 (10.6) | 119 (15.6) |  |
|  | 4 | 18 (28.1) | 274 (35.9) |  | 15 (31.9) | 273 (35.8) |  |
|  | 5 | 18 (28.1) | 188 (24.6) |  | 15 (31.9) | 188 (24.7) |  |
| **Dataset 9** | | | | | | | |
|  | | **Before matching** | | | **After matching** | | |
|  | | Not anti-hypertensive drug user | Anti-hypertensive drug user | SMD | Not anti-hypertensive drug user | Anti-hypertensive drug user | SMD |
| n | | 64 | 763 |  | 46 | 763 |  |
| AGE, mean (SD) | | 61.95 (11.84) | 69.82 (9.82) | 0.723 | 65.72 (10.23) | 69.82 (9.82) | 0.409 |
| SEX_T7, n (%) | |  |  | 0.013 |  |  | 0.064 |
|  | 1 | 43 (67.2) | 508 (66.6) |  | 32 (69.6) | 508 (66.6) |  |
| BMI, mean (SD) | | 28.99 (5.07) | 29.00 (4.54) | 0.002 | 28.63 (4.49) | 29.00 (4.54) | 0.082 |
| HEART_FAILURE_T7, n (%) | |  |  | 0.328 |  |  | 0.329 |
|  | 0 | 55 (85.9) | 566 (74.2) |  | 40 (87.0) | 566 (74.2) |  |
|  | 1 | 2 (3.1) | 74 (9.7) |  | 2 (4.3) | 74 (9.7) |  |
|  | 2 | 7 (10.9) | 123 (16.1) |  | 4 (8.7) | 123 (16.1) |  |
| ATRIAL_FIBRILLATION_T7, n (%) | |  |  | 0.215 |  |  | 0.127 |
|  | 0 | 40 (62.5) | 543 (71.2) |  | 31 (67.4) | 543 (71.2) |  |
|  | 1 | 11 (17.2) | 122 (16.0) |  | 7 (15.2) | 122 (16.0) |  |
|  | 2 | 13 (20.3) | 98 (12.8) |  | 8 (17.4) | 98 (12.8) |  |
| STROKE_T7, n (%) | |  |  | 0.096 |  |  | 0.126 |
|  | 2 | 9 (14.1) | 134 (17.6) |  | 6 (13.0) | 134 (17.6) |  |
| KIDNEY_DISEASE_T7, n (%) | |  |  | 0.226 |  |  | 0.102 |
|  | 0 | 53 (82.8) | 655 (85.8) |  | 40 (87.0) | 655 (85.8) |  |
|  | 1 | 4 (6.2) | 68 (8.9) |  | 3 (6.5) | 68 (8.9) |  |
|  | 2 | 7 (10.9) | 40 (5.2) |  | 3 (6.5) | 40 (5.2) |  |
| CANCER_T7, n (%) | |  |  | 0.198 |  |  | 0.164 |
|  | 0 | 56 (87.5) | 618 (81.0) |  | 40 (87.0) | 618 (81.0) |  |
|  | 1 | 2 (3.1) | 51 (6.7) |  | 2 (4.3) | 51 (6.7) |  |
|  | 2 | 6 (9.4) | 94 (12.3) |  | 4 (8.7) | 94 (12.3) |  |
| DIABETES_impu_new, n (%) | |  |  | 0.043 |  |  | 0.126 |
|  | 0 | 48 (75.0) | 574 (75.2) |  | 37 (80.4) | 574 (75.2) |  |
|  | 1 | 13 (20.3) | 147 (19.3) |  | 7 (15.2) | 147 (19.3) |  |
|  | 2 | 3 (4.7) | 42 (5.5) |  | 2 (4.3) | 42 (5.5) |  |
| Cons_GP_Times_impu, mean (SD) | | 3.28 (2.58) | 4.86 (5.76) | 0.353 | 3.37 (2.78) | 4.86 (5.76) | 0.329 |
| Cons_Emergency_Times_impu, mean (SD) | | 0.25 (0.53) | 0.34 (0.77) | 0.129 | 0.20 (0.45) | 0.34 (0.77) | 0.221 |
| Cons_Hospital_Times_impu, mean (SD) | | 0.47 (1.61) | 0.45 (0.99) | 0.012 | 0.43 (1.80) | 0.45 (0.99) | 0.012 |
| Cons_Specialist_Times_impu, mean (SD) | | 0.47 (0.96) | 0.56 (3.06) | 0.039 | 0.52 (1.03) | 0.56 (3.06) | 0.016 |
| Cons_Clinic_Times_impu, mean (SD) | | "0.77 (1.46) | 1.19 (3.56) | 0.158 | 0.91 (1.63) | 1.19 (3.56) | 0.102 |
| Alcohol_frequency_impu, n (%) | |  |  | 0.160 |  |  | 0.152 |
|  | 1 | 7 (10.9) | 79 (10.4) |  | 4 (8.7) | 79 (10.4) |  |
|  | 2 | 14 (21.9) | 160 (21.0) |  | 8 (17.4) | 160 (21.0) |  |
|  | 3 | 26 (40.6) | 285 (37.4) |  | 20 (43.5) | 285 (37.4) |  |
|  | 4 | 10 (15.6) | 166 (21.8) |  | 9 (19.6) | 166 (21.8) |  |
|  | 5 | 7 (10.9) | 73 (9.6) |  | 5 (10.9) | 73 (9.6) |  |
| Alcohol_units_impu, n (%) | |  |  | 0.201 |  |  | 0.187 |
|  | 0 | 8 (12.5) | 81 (10.6) |  | 5 (10.9) | 81 (10.6) |  |
|  | 1 | 28 (43.8) | 371 (48.6) |  | 19 (41.3) | 371 (48.6) |  |
|  | 2 | 24 (37.5) | 234 (30.7) |  | 18 (39.1) | 234 (30.7) |  |
|  | 3 | 4 (6.2) | 77 (10.1) |  | 4 (8.7) | 77 (10.1) |  |
| Alcohol_units_6_impu, n (%) | |  |  | 0.300 |  |  | 0.222 |
|  | 1 | 34 (53.1) | 449 (58.8) |  | 25 (54.3) | 449 (58.8) |  |
|  | 2 | 27 (42.2) | 236 (30.9) |  | 18 (39.1) | 236 (30.9) |  |
|  | 3 | 2 (3.1) | 56 (7.3) |  | 2 (4.3) | 56 (7.3) |  |
|  | 4 | 1 (1.6) | 18 (2.4) |  | 1 (2.2) | 18 (2.4) |  |
|  | 5 | 0 (0.0) | 4 (0.5) |  | 0 (0.0) | 4 (0.5) |  |
| Smoke_impu, n (%) | |  |  | 0.230 |  |  | 0.290 |
|  | 1 | 4 (6.2) | 86 (11.3) |  | 4 (8.7) | 86 (11.3) |  |
|  | 2 | 1 (1.6) | 12 (1.6) |  | 0 (0.0) | 12 (1.6) |  |
|  | 3 | 37 (57.8) | 465 (60.9) |  | 25 (54.3) | 465 (60.9) |  |
|  | 4 | 22 (34.4) | 200 (26.2) |  | 17 (37.0) | 200 (26.2) |  |
| Snuff_chewing_tobacco_impu, n (%) | |  |  | 0.093 |  |  | 0.043 |
|  | 1 | 5 (7.8) | 45 (5.9) |  | 3 (6.5) | 45 (5.9) |  |
|  | 2 | 0 (0.0) | 0 (0.0) |  | 0 (0.0) | 0 (0.0) |  |
|  | 3 | 4 (6.2) | 39 (5.1) |  | 2 (4.3) | 39 (5.1) |  |
|  | 4 | 55 (85.9) | 679 (89.0) |  | 41 (89.1) | 679 (89.0) |  |
| Cod_liver_oil_omega3_impu, n (%) | |  |  | 0.203 |  |  | 0.137 |
|  | 0 | 41 (64.1) | 559 (73.3) |  | 31 (67.4) | 559 (73.3) |  |
|  | 1 | 11 (17.2) | 101 (13.2) |  | 7 (15.2) | 101 (13.2) |  |
|  | 2 | 4 (6.2) | 30 (3.9) |  | 2 (4.3) | 30 (3.9) |  |
|  | 3 | 8 (12.5) | 73 (9.6) |  | 6 (13.0) | 73 (9.6) |  |
| FRUIT_UNITS_T7, mean (SD) | | 1.84 (1.48) | 2.10 (1.96) | 0.145 | 1.91 (1.50) | 2.10 (1.96) | 0.104 |
| RED_MEAT_T7, n (%) | |  |  | 0.427 |  |  | 0.358 |
|  | 1 | 9 (14.1) | 46 (6.0) |  | 6 (13.0) | 46 (6.0) |  |
|  | 2 | 15 (23.4) | 161 (21.1) |  | 12 (26.1) | 161 (21.1) |  |
|  | 3 | 33 (51.6) | 506 (66.3) |  | 27 (58.7) | 506 (66.3) |  |
|  | 4 | 7 (10.9) | 39 (5.1) |  | 1 (2.2) | 39 (5.1) |  |
|  | 5 | 0 (0.0) | 11 (1.4) |  | 0 (0.0) | 11 (1.4) |  |
| FRUITS_VEG_BERRY_T7, n (%) | |  |  | 0.204 |  |  | 0.233 |
|  | 1 | 0 (0.0) | 8 (1.0) |  | 0 (0.0) | 8 (1.0) |  |
|  | 2 | 4 (6.2) | 30 (3.9) |  | 2 (4.3) | 30 (3.9) |  |
|  | 3 | 13 (20.3) | 152 (19.9) |  | 12 (26.1) | 152 (19.9) |  |
|  | 4 | 15 (23.4) | 213 (27.9) |  | 10 (21.7) | 213 (27.9) |  |
|  | 5 | 32 (50.0) | 360 (47.2) |  | 22 (47.8) | 360 (47.2) |  |
| LEAN_FISH_T7, n (%) | |  |  | 0.444 |  |  | 0.224 |
|  | 1 | 8 (12.5) | 19 (2.5) |  | 1 (2.2) | 19 (2.5) |  |
|  | 2 | 8 (12.5) | 102 (13.4) |  | 6 (13.0) | 102 (13.4) |  |
|  | 3 | 43 (67.2) | 534 (70.0) |  | 35 (76.1) | 534 (70.0) |  |
|  | 4 | 5 (7.8) | 97 (12.7) |  | 4 (8.7) | 97 (12.7) |  |
|  | 5 | 0 (0.0) | 11 (1.4) |  | 0 (0.0) | 11 (1.4) |  |
| FAT_FISH_T7, n (%) | |  |  | 0.299 |  |  | 0.267 |
|  | 1 | 8 (12.5) | 86 (11.3) |  | 6 (13.0) | 86 (11.3) |  |
|  | 2 | 29 (45.3) | 261 (34.2) |  | 20 (43.5) | 261 (34.2) |  |
|  | 3 | 25 (39.1) | 368 (48.2) |  | 18 (39.1) | 368 (48.2) |  |
|  | 4 | 1 (1.6) | 38 (5.0) |  | 1 (2.2) | 38 (5.0) |  |
|  | 5 | 1 (1.6) | 10 (1.3) |  | 1 (2.2) | 10 (1.3) |  |
| PHYS_ACTIVITY_LEISURE_T7, n (%) | |  |  | 0.306 |  |  | 0.161 |
|  | 1 | 14 (21.9) | 178 (23.3) |  | 10 (21.7) | 178 (23.3) |  |
|  | 2 | 37 (57.8) | 401 (52.6) |  | 25 (54.3) | 401 (52.6) |  |
|  | 3 | 9 (14.1) | 170 (22.3) |  | 9 (19.6) | 170 (22.3) |  |
|  | 4 | 4 (6.2) | 14 (1.8) |  | 2 (4.3) | 14 (1.8) |  |
| EXERCISE_T7, n (%) | |  |  | 0.258 |  |  | 0.390 |
|  | 1 | 5 (7.8) | 93 (12.2) |  | 2 (4.3) | 93 (12.2) |  |
|  | 2 | 10 (15.6) | 91 (11.9) |  | 9 (19.6) | 91 (11.9) |  |
|  | 3 | 12 (18.8) | 119 (15.6) |  | 8 (17.4) | 119 (15.6) |  |
|  | 4 | 18 (28.1) | 275 (36.0) |  | 13 (28.3) | 275 (36.0) |  |
|  | 5 | 19 (29.7) | 185 (24.2) |  | 14 (30.4) | 185 (24.2) |  |
| **Dataset 10** | | | | | | | |
|  | | **Before matching** | | | **After matching** | | |
|  | | Not anti-hypertensive drug user | Anti-hypertensive drug user | SMD | Not anti-hypertensive drug user | Anti-hypertensive drug user | SMD |
| n | | 64 | 763 |  | 46 | 760 |  |
| AGE, mean (SD) | | 61.95 (11.84) | 69.82 (9.82) | 0.723 | 64.76 (11.33) | 69.87 (9.80) | 0.482 |
| SEX_T7, n (%) | |  |  | 0.013 |  |  | 0.017 |
|  | 1 | 43 (67.2) | 508 (66.6) |  | 31 (67.4) | 506 (66.6) |  |
| BMI, mean (SD) | | 28.99 (5.07) | 29.00 (4.54) | 0.001 | 28.49 (4.64) | 29.00 (4.54) | 0.110 |
| HEART_FAILURE_T7, n (%) | |  |  | 0.385 |  |  | 0.327 |
|  | 0 | 55 (85.9) | 565 (74.0) |  | 38 (82.6) | 562 (73.9) |  |
|  | 1 | 1 (1.6) | 73 (9.6) |  | 1 (2.2) | 73 (9.6) |  |
|  | 2 | 8 (12.5) | 125 (16.4) |  | 7 (15.2) | 125 (16.4) |  |
| ATRIAL_FIBRILLATION_T7, n (%) | |  |  | 0.197 |  |  | 0.206 |
|  | 0 | 40 (62.5) | 543 (71.2) |  | 29 (63.0) | 542 (71.3) |  |
|  | 1 | 12 (18.8) | 123 (16.1) |  | 8 (17.4) | 123 (16.2) |  |
|  | 2 | 12 (18.8) | 97 (12.7) |  | 9 (19.6) | 95 (12.5) |  |
| STROKE_T7, n (%) | |  |  | 0.135 |  |  | 0.055 |
|  | 2 | 8 (12.5) | 132 (17.3) |  | 7 (15.2) | 131 (17.2) |  |
| KIDNEY_DISEASE_T7, n (%) | |  |  | 0.144 |  |  | 0.085 |
|  | 0 | 53 (82.8) | 653 (85.6) |  | 39 (84.8) | 651 (85.7) |  |
|  | 1 | 5 (7.8) | 67 (8.8) |  | 5 (10.9) | 67 (8.8) |  |
|  | 2 | 6 (9.4) | 43 (5.6) |  | 2 (4.3) | 42 (5.5) |  |
| CANCER_T7, n (%) | |  |  | 0.176 |  |  | 0.114 |
|  | 0 | 56 (87.5) | 619 (81.1) |  | 39 (84.8) | 616 (81.1) |  |
|  | 1 | 3 (4.7) | 52 (6.8) |  | 3 (6.5) | 52 (6.8) |  |
|  | 2 | 5 (7.8) | 92 (12.1) |  | 4 (8.7) | 92 (12.1) |  |
| DIABETES_impu_new, n (%) | |  |  | 0.093 |  |  | 0.117 |
|  | 0 | 46 (71.9) | 574 (75.2) |  | 35 (76.1) | 572 (75.3) |  |
|  | 1 | 14 (21.9) | 155 (20.3) |  | 8 (17.4) | 155 (20.4) |  |
|  | 2 | 4 (6.2) | 34 (4.5) |  | 3 (6.5) | 33 (4.3) |  |
| Cons_GP_Times_impu, mean (SD) | | 3.89 (4.49) | 4.80 (5.31) | 0.185 | 4.22 (5.03) | 4.79 (5.32) | 0.111 |
| Cons_Emergency_Times_impu, mean (SD) | | 0.27 (0.54) | 0.33 (0.76) | 0.092 | 0.26 (0.53) | 0.33 (0.76) | 0.101 |
| Cons_Hospital_Times_impu, mean (SD) | | 0.47 (1.61) | 0.45 (0.98) | 0.013 | 0.54 (1.85) | 0.45 (0.99) | 0.062 |
| Cons_Specialist_Times_impu, mean (SD) | | 0.80 (2.74) | 0.56 (3.11) | 0.082 | 0.98 (3.19) | 0.53 (3.03) | 0.145 |
| Cons_Clinic_Times_impu, mean (SD) | | 0.66 (1.22) | 1.15 (3.47) | 0.188 | 0.78 (1.38) | 1.15 (3.47) | 0.138 |
| Alcohol_frequency_impu, n (%) | |  |  | 0.171 |  |  | 0.270 |
|  | 1 | 6 (9.4) | 79 (10.4) |  | 2 (4.3) | 79 (10.4) |  |
|  | 2 | 14 (21.9) | 160 (21.0) |  | 9 (19.6) | 159 (20.9) |  |
|  | 3 | 27 (42.2) | 285 (37.4) |  | 21 (45.7) | 283 (37.2) |  |
|  | 4 | 10 (15.6) | 166 (21.8) |  | 9 (19.6) | 166 (21.8) |  |
|  | 5 | 7 (10.9) | 73 (9.6) |  | 5 (10.9) | 73 (9.6) |  |
| Alcohol_units_impu, n (%) | |  |  | 0.162 |  |  | 0.305 |
|  | 0 | 7 (10.9) | 81 (10.6) |  | 2 (4.3) | 81 (10.7) |  |
|  | 1 | 28 (43.8) | 371 (48.6) |  | 21 (45.7) | 370 (48.7) |  |
|  | 2 | 24 (37.5) | 233 (30.5) |  | 19 (41.3) | 231 (30.4) |  |
|  | 3 | 5 (7.8) | 78 (10.2) |  | 4 (8.7) | 78 (10.3) |  |
| Alcohol_units_6_impu, n (%) | |  |  | 0.325 |  |  | 0.319 |
|  | 1 | 33 (51.6) | 449 (58.8) |  | 26 (56.5) | 448 (58.9) |  |
|  | 2 | 28 (43.8) | 235 (30.8) |  | 18 (39.1) | 233 (30.7) |  |
|  | 3 | 2 (3.1) | 55 (7.2) |  | 2 (4.3) | 55 (7.2) |  |
|  | 4 | 1 (1.6) | 20 (2.6) |  | 0 (0.0) | 20 (2.6) |  |
|  | 5 | 0 (0.0) | 4 (0.5) |  | 0 (0.0) | 4 (0.5) |  |
| Smoke_impu, n (%) | |  |  | 0.230 |  |  | 0.292 |
|  | 1 | 4 (6.2) | 86 (11.3) |  | 3 (6.5) | 86 (11.3) |  |
|  | 2 | 1 (1.6) | 12 (1.6) |  | 0 (0.0) | 12 (1.6) |  |
|  | 3 | 37 (57.8) | 465 (60.9) |  | 27 (58.7) | 464 (61.1) |  |
|  | 4 | 22 (34.4) | 200 (26.2) |  | 16 (34.8) | 198 (26.1) |  |
| Snuff_chewing_tobacco_impu, n (%) | |  |  | 0.093 |  |  | 0.090 |
|  | 1 | 5 (7.8) | 45 (5.9) |  | 2 (4.3) | 45 (5.9) |  |
|  | 2 | 0 (0.0) | 0 (0.0) |  | 0 (0.0) | 0 (0.0) |  |
|  | 3 | 4 (6.2) | 39 (5.1) |  | 3 (6.5) | 39 (5.1) |  |
|  | 4 | 55 (85.9) | 679 (89.0) |  | 41 (89.1) | 676 (88.9) |  |
| Cod_liver_oil_omega3_impu, n (%) | |  |  | 0.179 |  |  | 0.262 |
|  | 0 | 42 (65.6) | 559 (73.3) |  | 30 (65.2) | 557 (73.3) |  |
|  | 1 | 12 (18.8) | 99 (13.0) |  | 10 (21.7) | 98 (12.9) |  |
|  | 2 | 3 (4.7) | 31 (4.1) |  | 1 (2.2) | 31 (4.1) |  |
|  | 3 | 7 (10.9) | 74 (9.7) |  | 5 (10.9) | 74 (9.7) |  |
| FRUIT_UNITS_T7, mean (SD) | | 1.77 (1.38) | 2.06 (1.97) | 0.174 | 1.93 (1.50) | 2.05 (1.96) | 0.068 |
| RED_MEAT_T7, n (%) | |  |  | 0.440 |  |  | 0.281 |
|  | 1 | 10 (15.6) | 46 (6.0) |  | 6 (13.0) | 46 (6.1) |  |
|  | 2 | 14 (21.9) | 160 (21.0) |  | 11 (23.9) | 159 (20.9) |  |
|  | 3 | 32 (50.0) | 511 (67.0) |  | 26 (56.5) | 510 (67.1) |  |
|  | 4 | 7 (10.9) | 36 (4.7) |  | 2 (4.3) | 35 (4.6) |  |
|  | 5 | 1 (1.6) | 10 (1.3) |  | 1 (2.2) | 10 (1.3) |  |
| FRUITS_VEG_BERRY_T7, n (%) | |  |  | 0.257 |  |  | 0.241 |
|  | 1 | 0 (0.0) | 9 (1.2) |  | 0 (0.0) | 9 (1.2) |  |
|  | 2 | 5 (7.8) | 30 (3.9) |  | 3 (6.5) | 30 (3.9) |  |
|  | 3 | 11 (17.2) | 153 (20.1) |  | 9 (19.6) | 152 (20.0) |  |
|  | 4 | 15 (23.4) | 212 (27.8) |  | 10 (21.7) | 212 (27.9) |  |
|  | 5 | 33 (51.6) | 359 (47.1) |  | 24 (52.2) | 357 (47.0) |  |
| LEAN_FISH_T7, n (%) | |  |  | 0.452 |  |  | 0.275 |
|  | 1 | 8 (12.5) | 19 (2.5) |  | 3 (6.5) | 18 (2.4) |  |
|  | 2 | 9 (14.1) | 100 (13.1) |  | 6 (13.0) | 99 (13.0) |  |
|  | 3 | 42 (65.6) | 533 (69.9) |  | 32 (69.6) | 532 (70.0) |  |
|  | 4 | 5 (7.8) | 99 (13.0) |  | 5 (10.9) | 99 (13.0) |  |
|  | 5 | 0 (0.0) | 12 (1.6) |  | 0 (0.0) | 12 (1.6) |  |
| FAT_FISH_T7, n (%) | |  |  | 0.284 |  |  | 0.254 |
|  | 1 | 8 (12.5) | 88 (11.5) |  | 6 (13.0) | 87 (11.4) |  |
|  | 2 | 29 (45.3) | 263 (34.5) |  | 19 (41.3) | 261 (34.3) |  |
|  | 3 | 25 (39.1) | 367 (48.1) |  | 20 (43.5) | 367 (48.3) |  |
|  | 4 | 1 (1.6) | 35 (4.6) |  | 1 (2.2) | 35 (4.6) |  |
|  | 5 | 1 (1.6) | 10 (1.3) |  | 0 (0.0) | 10 (1.3) |  |
| PHYS_ACTIVITY_LEISURE_T7, n (%) | |  |  | 0.280 |  |  | 0.141 |
|  | 1 | 14 (21.9) | 166 (21.8) |  | 10 (21.7) | 165 (21.7) |  |
|  | 2 | 37 (57.8) | 405 (53.1) |  | 27 (58.7) | 404 (53.2) |  |
|  | 3 | 9 (14.1) | 173 (22.7) |  | 8 (17.4) | 172 (22.6) |  |
|  | 4 | 4 (6.2) | 19 (2.5) |  | 1 (2.2) | 19 (2.5) |  |
| EXERCISE_T7, n (%) | |  |  | 0.222 |  |  | 0.263 |
|  | 1 | 6 (9.4) | 93 (12.2) |  | 3 (6.5) | 93 (12.2) |  |
|  | 2 | 10 (15.6) | 88 (11.5) |  | 8 (17.4) | 87 (11.4) |  |
|  | 3 | 12 (18.8) | 120 (15.7) |  | 7 (15.2) | 120 (15.8) |  |
|  | 4 | 18 (28.1) | 274 (35.9) |  | 15 (32.6) | 273 (35.9) |  |
|  | 5 | 18 (28.1) | 188 (24.6) |  | 13 (28.3) | 187 (24.6) |  |
| SMD, standardized mean difference | | | | | | | |

Additional file: Table S7. Pooled results from the sensitivity analysis for the logistic regression analyses of the multiple imputed datasets, using propensity score matching without replacement*.

| Exposure variable | Outcome variable | Odds ratio | 95 % confidence interval |
| --- | --- | --- | --- |
| Use of lipid lowering drugs | Achievement of treatment goal for LDL-cholesterol | 17.3 | 5.3-56.4 |
| Use of antihypertensive drugs | Achievement of treatment goal for blood pressure | 1.5 | 0.6-3.6 |
| ^*^ Number of cases varied between datasets and can be found in Additional file: Tables S5 and S6 | | | |
